# Hepatitis C-like viruses are produced in cells from rabbit and hare DNA

**DOI:** 10.1038/srep14535

**Published:** 2015-09-29

**Authors:** Eliane Silva, Hugo Osório, Gertrude Thompson

**Affiliations:** 1 Department of Veterinary Clinics, Instituto de Ciências Biomédicas de Abel Salazar da Uiversidade do Porto, Rua de Jorge Viterbo Ferreira, 228, 4050-313 Porto, Portugal; 2 Centro de Investigação em Biodiversidade e Recursos Genéticos (CIBIO), Research Network in Biodiversity and Evolutionary Biology (InBio), Universidade do Porto, Rua Padre Armando Quintas, 7, 4485-661 Vairão, Portugal; 3 Instituto de Investigação e Inovação em Saúde, Universidade do Porto, Rua Alfredo Allen, s/n, 4200-135 Porto, Portugal; 4 Institute of Molecular Pathology and Immunology of the University of Porto (IPATIMUP), Rua Dr. Roberto Frias, S/N, 4200-465 Porto, Portugal; 5 Faculty of Medicine da University of Porto, Al. Prof. Hernâni Monteiro, 4200-319 Porto, Portugal

## Abstract

Hepatitis C virus (HCV), a major causative agent of acute and chronic liver disease, belongs to the *Flaviviridæ* family and contains a single-strand positive-sense RNA genome, which upon virus entry and uncoating, functions as mRNAs and thus can be directly translated into proteins by host cell machinery. To date the HCV origin remains unclear and HCV life cycle and pathogenesis are not enlightened processes due to the absence of HCV efficient cell cultures systems or animals models. Here we show that rabbit and hare HCV-like viruses, RHCV and HHCV respectively, are formed after the inoculation of genomic DNA in Madin-Darby bovine kidney cell line cultures. RHCV is closely related to the HCV-1a/HCV-1b genotypes and HHCV is more closely related to the HCV-1b genotype. These findings could contribute to the understanding of HCV origin as well as clarify the virus life cycle, pathogenesis, evolution and diversity.

Positive RNA viruses are characterized by a single-strand positive-sense RNA (ssRNA+) genome, which, upon virus entry and uncoating, functions as mRNAs and thus can be directly translated into proteins by host cell machinery^1^. Therefore, the genomic RNA could work as template for viral RNA replication. Following translation and processing of the viral polyprotein(s), viral nonstructural (NS) proteins, viral RNA, and host factors form membrane-associated replication complexes that carry out viral RNA synthesis^2^,^3^. The resultant progeny positive RNA strands can either initiate a new translation cycle or be packaged into virions that are subsequently released to infect naïve cells^2^. Moreover, host factors participate in almost all or probably in all steps of ssRNA+ virus infection, including entry, viral gene expression, virion assembly and release^2^. Furthermore, host factors are targeted by ssRNA+ viruses to modulate host gene expression and defenses^2^.

Studying the presence and behavior of endogenous viral elements (EVEs) having ancestries with ssRNA+ viruses in species genomes is another interesting challenge because all genetic processes involving them are poorly understood. Most RNA virus EVEs identified to date are derived from RNA viruses with negative-sense genomes or from RNA viruses that lack DNA intermediates^4^,^5^,^6^. EVEs derived from flavivirus-related RNA viruses were first described in insects in 2004^7^. This description involves a gene sequence of an RNA virus that replicate using an RNA-dependent RNA polymerase integrated into the Aedes spp. mosquitoes genome^7^. Recently, Katzourakis and Gifford described the presence of EVEs derived from RNA viruses, ssRNA+viruses included, in animal genomes and this has revolutionized the understanding of the processes and time-scale of viral evolution^8^. More recently, EVEs that share a sequence similarity to ssRNA+ viruses of plants integrated into the genomes of insects have also been reported^9^. Although, EVEs derived from ssRNA+ viruses appearing in extremely low copy numbers in the genomes; one genomic copy in the case of the *Reoviridae*, five in the case of the *Flaviviridae* and a small number in Potato virus Y were previously described^10^.

Hepatitis C virus (HCV), a major causative agent of acute and chronic liver disease, belongs to the *Flaviviridæ* family and contains an ssRNA+ genome of ~9,600 nucleotides that encodes a polyprotein precursor of ~3,000 amino acids^11^. Consequently and as described previously, probably the ssRNA+ genome could function as mRNAs that can be directly translated into proteins by host cell machinery and genomic RNA could function as template for viral RNA replication.

We recently described the presence of endogenous HCV homolog fragments in wild/domestic rabbits (*Oryctolagus cuniculus*) and hare (*Lepus europaeus*) genomes and the capacity of these small endogenous fragments to replicate in Mardin-Darby Bovine Kidney (MDBK) cell line cultures^12^. On one hand, we described a new cell culture system that probably could support the infection and replication of HCV viruses. It is well known that HCV investigation has been limited by the lack of an efficient cell culture system permissive for HCV infection and replication. On the other hand, understanding if these small fragments, small EVEs, would be able to produce entire viral particles with infectious proprieties in this cell line is required and this is the major goal of this study. For this purpose, because the small EVEs fragments are endogenous to rabbit and hare genomes, DNA extractions from livers homogenates of one domestic rabbit and one hare, samples used in ours previous work^12^, were performed and subjected to RNAse treatment and directly inoculated in naïve MDBK cell cultures. Next, the infectivity of DNA samples and formation of entire HCV-like virus particles in the inoculated naïve MDBK cells were evaluated by immunogold electron microscopy (IEM) with monoclonal antibodies for the NS5 and E2 HCV specific proteins, quantitative Real Time-RT-PCR (qRT-PCR), western blot analysis with monoclonal antibodies for the NS5 HCV specific protein and matrix-assisted laser desorption/ionization time-of-flight/time-of-flight (MALDI-TOF/TOF-MS/MS) mass spectrometry. Moreover, phylogenetic analysis of a final consensus constructed sequence for each tested sample revealed the presence of viruses genetically similar to HCV, rabbit and hare HCV-like viruses, tentatively named RHCV and HHCV respectively and that RHCV is closely related to the HCV-1a/HCV-1b genotypes and HHCV is more closely related to the HCV-1b genotype. Our results suggests the possibility that HCV may have been introduced into the human population after their contact with genetic material containing small EVEs homologous to HCV, reinforcing that HCV is not restricted to primates. HCV origin and understanding HCV life cycle are fields that could be better understood with the support on revealed presented data.

## Results

### IEM of rabbit and hare DNA samples

**Figure 1:**
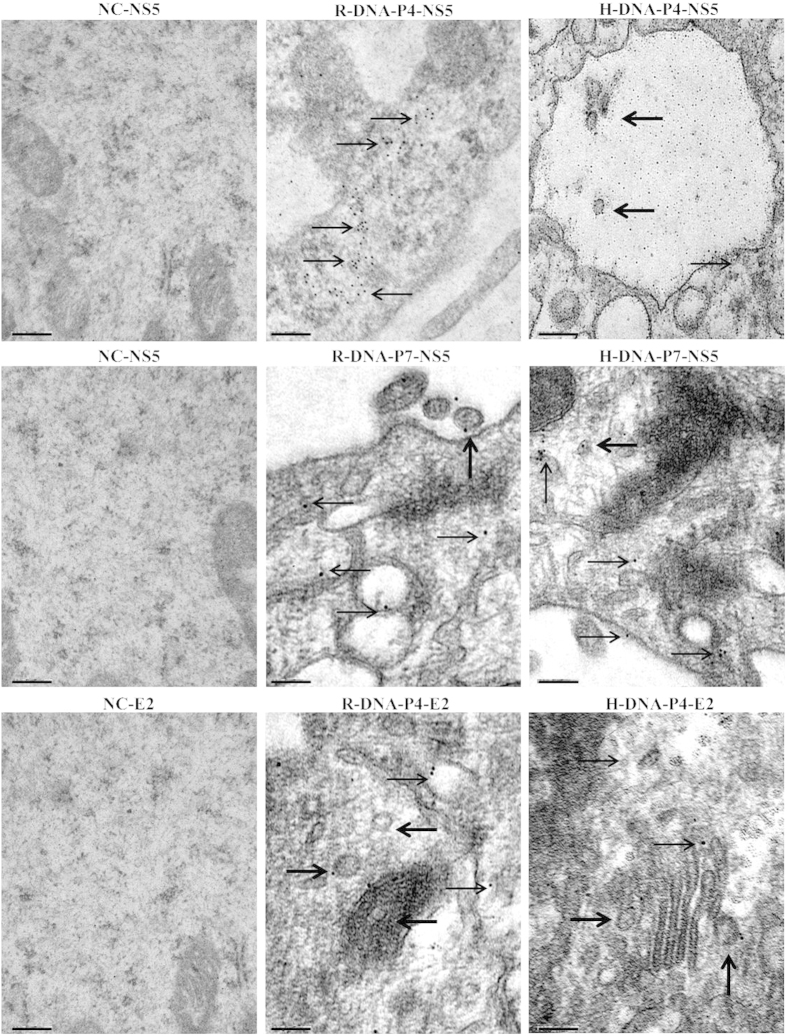
IEM detection of HCV E2 and NS5 proteins in MDBK cells. The cells inoculated with DNA extracted from liver homogenates of domestic rabbit and a hare were analyzed 28 (P4, E2 and NS5 proteins) and 49 (P7, NS5 protein) days post infection. These cells and the negative control (NC), uninoculated MDBK cells, were incubated with monoclonal antibodies specific for the HCV E2 and NS5 proteins and specific immunostaining was detected only in inoculated rabbit and hare samples. Visible HCV virus-like particles with specific immunostaining for the HCV E2 and NS5 proteins are indicated by arrows. The immunogold particles were 10 nm in diameter. Scale bars: 200 nm.

Cell suspensions of passages (P) P4 and P7 from each MDBK cell flask that were inoculated with DNA from the rabbit and hare liver samples, were evaluated with the use of mouse monoclonal antibodies anti-HCV NS5 protein, and specific immunostaining was demonstrated (Fig. 1). Furthermore, cells suspensions of passage P4 of the tested samples were also evaluated by IEM using mouse monoclonal antibodies anti-HCV E2 protein. Specific immunostaining could be observed including the visualization of HCV-like particles clearly marked by the immunogold particles attached to the HCV E2 protein (Fig. 1). Negative controls (uninoculated MDBK cells) were included and treated with the selected monoclonal antibodies and no reaction was detected (Fig. 1).

### qRT-PCR of rabbit and hare DNA samples

To evaluate the infectivity and replication of HCV-like particles, HCV RNA titers (in duplicate) were evaluated by qRT-PCR from extracted RNA of the inoculated MDBK cells with rabbit and hare DNA samples at passages P1 and P2. Virus RNA titers were detected at 4.39 log RNA copies ml^−1^ (P1) and 3.75 log RNA copies ml^−1^ (P2) from the rabbit DNA sample and 4.46 log RNA copies ml^−1^ (P1) and 4.61 log RNA copies ml^−1^ (P2) from the hare DNA sample.

### Western blot analysis of HCV NS5B protein

To further evaluate the infectivity of the HCV-like particles obtained in MDBK cells, new naïve MDBK cells were inoculated with supernatants of the inoculated MDBK cells (with rabbit and hare DNA samples) (P2). A band with a molecular mass of ~65 kDa corresponding to the HCV NS5B protein was detected by the western blot analysis of, both samples (Supplementary Fig. S1). Furthermore, the HCV NS5B protein was undetected in unionoculated MDBK cells (negative control) (Supplementary Fig. S1).

### MALDI-TOF/TOF-MS/MS analysis

To further confirm the infectivity and efficient HCV-like particles production in MDBK cells, the supernatants of the *de novo* naïve MDBK cells inoculated with rabbit and hare samples (P2) as described above were used. Extracted proteins recovered from the cells were separated by SDS-PAGE and stained with Coomassie Blue protein stain, 7 days post inoculation. The visible protein bands matching HCV specific proteins were selected on the basis of their molecular weight and excised from the gel followed by MALDI-TOF/TOF-MS/MS mass spectrometry analysis as described in methods section. For each digested sample MS/MS spectra were blasted in the MASCOT database search against HCV sequences deposited at UniProt database and a total of 2,338 peptide sequences, such as F, Core, E1, E2, p7, NS2, NS3, NS4A, NS4B, NS5A and NS5B HCV specific proteins, were identified with significant protein scores (P.S.) (P.S. > 64 are significant, p < 0.05), of which 1,694 and 644 peptide sequences were from the rabbit and hare DNA samples respectively (Table 1, Supplementary Table S2 and S3). Moreover, a MS/MS peptide sequences summary reported by Mascot version 2.4 for both tested samples presented 51 matches for F, 99 matches for Core, 59 matches for Core/E1, 83 matches for Core/E1/E2, 86 matches for E2, 96 matches for E2/p7/NS2/NS3, 74 matches for NS2, 221 matches for NS3, 99 matches for NS3-4A, 169 matches for NS4B, 338 matches for NS5A, 76 matches for NS5A/NS5B and 253 matches for NS5B HCV specific proteins for rabbit and 44 matches for F, 31 matches for Core, 40 matches for E1, 75 matches for E2, 83 matches for NS3, 79 matches for NS3-4A, 30 matches for NS4A/NS4B, 221 matches for NS5A and 41 matches for NS5B HCV specific proteins for hare samples with significant P.S. can be observed in Table 1. Complete MS/MS peptide sequences reported by Mascot version 2.4 including blast information, such as UniProt accession nos., HCV genotypes, calculated masses (Da), HCV genome positions (amino-acid), P.S., expected-values and peptide sequences for each sample can be observed in Supplementary Table S1 and S2 for tested rabbit and hare DNA samples inoculated in MDBK cells respectively. Furthermore, different HCV genotypes matched animal samples genetic sequences when they were blasted against HCV. Identified peptide sequences that matched with unidentified, 1a, 1b, 3a, 3b HCV genotypes and unidentified, 1a, 1b and 3 genotypes can be observed for rabbit and hare samples respectively (Supplementary Table S2 and S3). The spectra generated by MS/MS analysis of all identified peptide sequences for both tested animal samples can be observed in Supplementary Fig. S2. Proteins extracted from uninoculated MDBK cells (negative control) were also separated by SDS-PAGE procedures and stained with imperial protein stain and no bands with similar molecular mass to HCV proteins were observed.

When blastp of constructed rabbit HCV-like viruses amino acids consensus sequences for specific HCV proteins were conducted in the UniProt database (http://www.uniprot.org/blast/), similarities of 100%–81%; e-values of 0.0–1.0 × 10^−139^, including different HCV genotypes/subtypes, were shared for core, E1, E2, p7, NS2, NS3, NS4A, NS4B, NS5A and NS5B HCV specific proteins (Supplementary Table S5). The final RHCV amino-acid consensus sequence shared the genome polyprotein of HCV-1a and -1b genotypes with similarities of 78%–77% and e-values of 0.0–0.0 (Supplementary Table S5). Moreover, the constructed hare HCV-like viruses amino-acid consensus sequences for specific HCV proteins, shared similarities of 100%–78%; e-values of 0.0–8.0 × 10^−166^, including different HCV genotypes/subtypes, for core, E1, E2, p7, NS2, NS3, NS4A, NS4B, NS5A and NS5B HCV specific proteins. The final HHCV amino-acid consensus sequence, shared with genome polyprotein of HCV, HCV- 1a and -1b genotypes similarities of 72%–70% and e-values of 0.0–0.0 (Supplementary Table S6). This data supported the selection of the HCV genotypes/subtypes used in the phylogenetic analysis.

### Phylogenetic analysis of RHCV and HHCV

**Figure 2:**
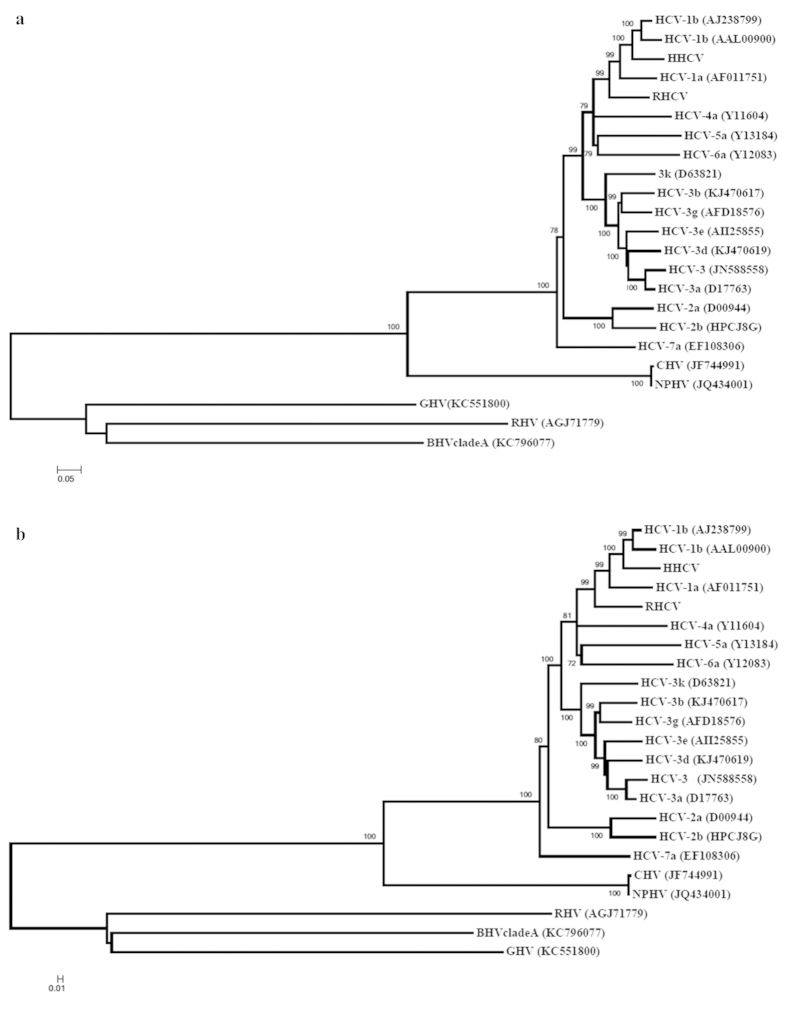
Phylogenetic analysis of RHCV and HHCV. Phylogenetic analysis using partial CDS sequences of reported HCV-like viruses recovered from *de novo* naïve MDBK cells inoculated with supernatants of the first rabbit and hare DNA samples inoculated in naive MDBK cells. (**a**) NJ and (**b**) ML methods were inferred. Bootstrap resampling was used to determine the robustness of branches; values of ≥ 72% (from 1,000 replicates) are shown. Reported HCV-like viruses are RHCV (rabbit) and HHCV (hare) respectively. A listing of host species, virus abbreviations and original accession numbers for sequences used in the phylogenetic trees are provided in Supplementary Table S3.

Phylogenetic relationships were inferred using the NJ and the ML methods as described in methods section. RHCV and HHCV were phylogenetically classified by determining the genetic relatedness to representative viruses of genus *Hepacivirus*. Notably, this phylogenetic analysis, using both methods, placed RHCV and HHCV as more closely related to the seven HCV genotypes, supported by strong bootstrap values of 99%–100% (of 1,000 replicates) (Fig. 2). CHV, NPHV, and RHV; GHV and BHVcladeA formed a second and third cluster respectively, both also supported by a strong (100%) bootstrap value of 1,000 replicates (Fig. 2). Furthermore, the identified RHCV is more closely related to HCV-1a genotype and the identified HHCV is more closely related to HCV-1b genotypes (Fig. 2). The phylogenetic analysis clearly demonstrates that these newly produced rabbit and hare HCV-like are more closely related to HCV than the described animal hepacivirus used in this analysis. In this study, the phylogenetic analysis of known hepaciviruses CHV, NPHV, RHV, GHV and BHVcladeA, showed clustering concordance with data previously described^13^,^14^,^15^. These results clearly suggest that entire HCV-like particles are produced after the inoculation of rabbit and hare DNA in MDBK cells, which is in accordance with the visualization by IEM of entire HCV-like particles.

## Discussion

In our previous study, the presence of endogenous HCV homolog fragments in wild/domestic rabbits (*Oryctolagus cuniculus*) and hare (*Lepus europaeus*) genomes and their capacity to replicate in MDBK cell cultures were detected by PCR and RT-PCR and MALDI-TOF/TOF-MS/MS analysis^12^. We have demonstrated that several homolog fragments to different HCV specific proteins are integrated in the rabbit and hare genomes^12^; (see Table 1 and 2 from this reference). Furthermore, we showed by blastn between HCV-1b (ID - D90208, HCV database; individual proteins and polyprotein) and *O. cuniculus* and by blastn between HCV specific primers (used for PCRs procedures) and *O. cuniculus*, that the sequences matched with high degree of homology (100%–91%) with several distinct scaffold or chromosomes in the rabbit genome. Some of the homolog base pair sequences found within the same fragment matched sequences that appeared repeatedly in several distinct scaffold or chromosomes^12^, (see Table S4 and S6 from this reference). Moreover, the results obtained have showed that all these small endogenous fragments homologous to HCV-like proteins are integrated in the rabbit and hare genomes and were detected in high copy numbers in the studied samples^12^. These findings lead us to hypothesise that the rabbit and the hare have small EVEs homologous to all HCV genetic specific regions that, when exposed to a suitable biological substrate, in these case the MDBK cell line, are able to internalize the cells and initiate its own replication using the host cell machinery to produce proteins and polyproteins and likely entire HCV-like particles.

EVEs derived from a flavivirus-related RNA involving a gene sequence of the virus RNA that replicates using an RNA-dependent RNA polymerase integrated into the Aedes spp. mosquitoes genome was previously described^7^. Based in these findings and in the functional pathways of ssRNA+ viruses genomes, such as, the virion (genomic) RNA is the same sense as mRNA and therefore can function as mRNA that can be immediately translated by host cell machinery upon infection, working the genomic RNA as template for viral RNA replication^1^,^2^,^3^, we decided to look at the functional and biological significance of these genetic elements, smalls EVEs HCV homolog’s, present in the rabbits and hares genomes.

The evidence that HCV cell entry requires essential receptors or coreceptors like human cluster of differentiation 81, scavenger receptor class B type I, claudin 1 and occluding were previously described^16^,^17^,^18^,^19^,^20^. The expression of claudin 1 in bovine kidneys, a sequence of the bovine scavenger receptor class B type I with close identity to human sequences, occludin, that have been identified in high concentrations in tight junctions of the MDBK cell line, and that cluster of differentiation 81 is expressed in several bovine tissues including the liver and Kidney were also previously reported^21^,^22^,^23^,^24^,^25^ reinforcing our present research.

In this study, we showed that RHCV and HHCV are produced upon the inoculation of MDBK cells with rabbit/hare genomic DNA, and that the resulting RHCV is closely related to HCV-1a/HCV-1b genotypes and HHCV is more closely related to HCV-1b genotype. Our conclusions are supported by the following observations. First, we demonstrated that entire rabbit and hare HCV-like particles are formed in MDBK cells upon the inoculation of naïve MDBK cells with DNA extracted from domestic rabbit and from a hare. HCV-like particles were demonstrated by IEM and with the use of specific immunostaining that detected the NS5 and E2 HCV proteins. Immunogold particles were detected in several cellular organelles of infected cells such as vacuoles, lipid droplets, mitochondria and Golgi apparatus in agreement with previous observations^12^,^26^,^27^. Secondly, we detected HCV RNA titers of 4.39 log copies ml^−1^ (P1), 3.75 log copies ml^−1^ (P2) for the rabbit DNA passages in cells; 4.46 log copies ml^−1^ (P1), 4.61 log copies ml^−1^ (P2) for the hare DNA cell passages when targeting the HCV 5′ untranslated region by qRT-PCR. Thus, the obtained HCV RNA titers indicates the production of entire HCV-like particles in the inoculated cells which is in accordance to data previously described, when the production of infectious HCV-1a genotype was evaluated in cultured human hepatoma cells^28^. Thirdly, we were able to detect NS5B HCV specific protein in *de novo* inoculated naïve MDBK cells by western blot analysis. These results demonstrate that the generated HCV-like viruses have the capacity to infect the *de novo* inoculated naïve MDBK cells, using both types of samples. Finally, the identification of several peptide sequences that matched with all specific structural and nonstructural HCV proteins by MALDI-TOF/TOF-MS/MS analysis of each tested sample, in the *de novo* inoculated naïve MDBK cells, allowed its genetic relationship with HCV. The final consensus of the amino-acid sequence construction resulted in rabbit RHCV and hare HHCV amino-acid sequences, consisting of almost all HCV polyproteins. Herewith, RHCV and HHCV sequences were identified and classified as genetically related to HCV, supported by the obtained blastp results of the identified individual and final consensus sequences of HCV proteins in the UniProt database and by phylogenetic analysis. Moreover, RHCV was more closely related to HCV-1a/HCV-1b genotypes and HHCV was more closely related to HCV-1b genotype. Furthermore, the newly formed and identified HCV-like viruses are the most closely related to HCV genotypes described to date, when compared with others HCV-like viruses detected in the dog, horse, rodents or bats^13^,^14^,^15^.

For the best of our knowledge, this is the first report on the generation of HCV-like viruses in a cell culture system. We clearly demonstrate that entire HCV-like particles are produced in MDBK cell line, suggesting that the small EVEs present in the genomic DNA of the rabbit and hare can internalize the MDBK cells and together with the cell machinery initiate replication and the generation of novel HCV-like virus, and that probably HCV could also be generated likewise. However, the detailed mechanisms of this process and a comprehensive knowledge of the biological and medical significance of our findings should be further investigated. This could be a breakthrough in clarifying the theories of the HCV origin, evolution, the improvement of the HCV life cycle understanding and the opening of new horizons to predict/evaluate new therapeutic approaches for this dangerous pathogen.

## Methods

### Samples

Total DNA extracted from liver homogenates of one domestic rabbit *(Oryctolagus cuniculus*) and one hare *(Lepus europaeus),* samples used in our previous study^12^. All legal requirements and permits by the Instituto da Conservação da Natureza e da Biodiversidade (ICNB) (the national authority for nature conservation and wildlife protection) for rabbit and hare specimens collection and use were followed. The ICNB permit is issued under the EU Habitats Directive, considering safety measures to avoid spread of any pathology, namely the disinfection of equipment with 1% hypochlorite solution of dead animal species. All experimental protocol were carried out in accordance with the approved guidelines by the Portuguese Veterinary Authority of the Ministry for Agriculture, Sea, Environment and Spatial Planning (Decree law n.° 113/2013 of 7^th^ August 2013) were the current European Communities Council Directive of September 2010 (2010/63/UE) is present. The methods were carried out in accordance with the approved guidelines.

### Cell cultures

MDBK cell line (American Type Culture Collection) obtained from the Friedrich-Loeffler-Institute (Insel Riems, Germany) were maintained as previously described^12^.

### DNA extraction from liver homogenates samples

Total DNA was extracted from 10% (w/v) liver homogenates samples treated with RNAse A using the QIAamp^®^ DNA blood kit (Qiagen, Hilden, Germany) according to the manufacturer instructions. DNA, RNA and protein concentrations were measured before sample inoculation in MDBK cells in a Qubit^®^ Fluorometer (Invitrogen, Carlsbad, USA) according to the manufacturer instructions (Supplementary Table S1).

### Rabbit and hare liver DNA inoculation in naïve MDBK cells

Samples inoculations were processed according to procedures previously described^12^. Briefly, cell culture medium of a T25 cell culture flask with MDBK cells (80% in confluence) was discarded and then inoculated with extracted DNA (200 μl; 2.5 ng ml^−1^) of rabbit liver homogenate and incubated at 37 °C and 5% CO_2_ for 4 h (adsorption). After, the cell monolayer was washed twice with 1X phosphate buffered saline (PBS), replaced with new cell culture medium and maintained at 37 °C and 5% CO_2_ for 7 days. Then, 7 passages were performed subsequently, with 7days incubation period between each passage. For the hare DNA (200 μl; 640 ng ml^−1^) sample the same procedure as for rabbit was undertaken. Cells were evaluated for HCV detection at indicated passages post inoculation by IEM and qRT-PCR analysis. Uninoculated MDBK cells were maintained and included as negative controls in all procedures.

### MDBK cells *de novo* inoculated with supernatants recovered from the first MDBK cells inoculation with DNA samples (liver)

To further evaluate the infectivity and efficient production of HCV-like particles, supernatants of previously inoculated MDBK cells with liver DNA samples were used as inoculums in *de novo* naïve MDBK cells. For this, 2 ml of supernatant filtered through a 0.2 μm membrane obtained from passage P2 was adsorbed onto T150 tissue culture flasks with fresh naïve MDBK cells, 80% in confluence, and incubated at 37 °C and 5% CO_2_ for 4 h. After, the monolayers were washed twice with 1X PBS, the culture medium was replaced and flasks were maintained at 37 °C and 5% CO_2_ for 7 days. The supernatants of each flask were then evaluated at 7 days post inoculation by MALDI TOF/TOF-MS/MS analysis. Uninoculated MDBK cells (negative controls) were maintained and equally performed as samples.

### Immuno electron microscopy

For IEM, the cell suspensions of passages P4 and P7 of each MDBK cell flask inoculated with liver DNA of rabbit and hare together with the negative controls (uninoculated cells) were processed according to procedures previously described^12^ with one modification. After the blocking process with BSA (Sigma-Aldrich, Steinheim, Germany) in Tris Buffered Saline, the grids were incubated for 60 min on a drop of specific monoclonal antibodies for the NS5 (P4 and P7) and E2 (P4) HCV specific proteins.

### RNA extraction and HCV RNA quantification by qRT-PCR

Cell lysates from passages P1 and P2 were of the inoculated cells with rabbit and hare DNA, were collected and subjected to three freeze/thaw cycles at −80 °C / room temperature. Total RNA extractions were performed using the QIAamp Viral RNA Kit (Qiagen, Hilden, Germany) following the manufacturer instructions with some modifications. Briefly, 100 μL of treated supernatants and cells plus 5 μL of internal control (provided in HCV Real-TM Quant kit) were extracted and RNAs were eluted with 50 μL of buffer AVE. After, the HCV RNA titers (in duplicate) from extracted RNA were determined by qRT-PCR targeting the HCV 5′ untranslated region, HCV Real-TM Quant kit (Sacace Biotechnologies Srl, Como, Italy), according to the manufacturer’s instructions in a StepOne™ Real-Time PCR System (Applied Biosystems, Foster, California). Uninoculated MDBK cells maintained as negative controls were treated as samples.

### Western blot of HCV NSB Protein

Western blot procedures were performed as previously described^12^,^29^ with some modifications. In brief, total protein extracts from supernatants at 7 days post inoculation recovered from new naïve MDBK cells inoculated with rabbit and hare samples and an uninoculated naïve MDBK cells (negative control) were mixed with sodium dodecyl sulfate reducing buffer, denatured for 5 minutes (boiling), and loaded onto a 12% precast sodium dodecyl sulfate-polyacrylamide gel (Bio-Rad, Hercules, USA). After electrophoresis, proteins were transferred to a PVDF membrane (Bio-Rad, Hercules, USA) for 1 hour at 100 V. The membrane was blocked for 1 hour with 5% nonfat dry milk (Molico-Nestlé, Vevey, Switzerland) in tris buffered saline with 0.05% Tween 20 (Merck, Darmstadt, Germany) at room temperature. The membrane was incubated overnight with mouse monoclonal antibodies anti-HCV NS5 (Santa Cruz Biotechnology, Inc, Heidelberg, Germany) (1:100) and subsequently incubated with goat anti-mouse IgG-alkaline phosphatase (Sigma-Aldrich, Saint Louis, USA) (1:200) antibody for 60 minutes. Finally, proteins were visualized by chemiluminescence using an ECF substrate (GE Healthcare Amersham, Freiburg, Germany).

### MALDI-TOF/TOF-MS/MS mass spectrometry in *de novo* inoculated MDBK cells

The supernatants (7 days) recovered from the new naïve MDBK cells inoculated with rabbit and hare samples, as described above, were harvested and filtered through a 0.2 μm membrane, for protein extraction and MALDI TOF/TOF-MS/MS analysis as previously described^12^,^30^ with one modification. The mass spectrometry approaches were performed in a 4800 Plus MALDI TOF/TOF Analyzer (AB SCIEX, Framingham, MA). MS data was further treated to obtain a consensus sequence from the rabbit and hare DNA samples as described below. Uninoculated MDBK cells (negative control) were also included for protein extraction and separated by SDS-PAGE and stained with imperial protein stain. The mass spectrometry proteomics data have been deposited to the ProteomeXchange Consortium via the PRIDE (http://www.ebi.ac.uk/pride/archive/) partner repository with the dataset identifier PXD001992.

### Drawing Consensus Sequences

Two final consensus sequences of the almost complete HCV-like viruses polyprotein, rabbit (RHCV) and hare (HHCV) HCV-like viruses, were constructed using recovered peptide sequences from *de novo* inoculated naïve MDBK cells determined by MALDI TOF/TOF-MS/MS analysis. Thus, these final consensus sequences for RHCV and HHCV were obtained by complete sequences and by consensus sequences construction of the significant peptide sequences identified for the different HCV-like structural and non-structural proteins that matched with several UniProt accession nos. Therefore, core protein (F protein included) consensus sequence was constructed using recovered peptide sequences that matched with B3TKW7, F4YQ96 B8QB25 and F1A6I7, E1 protein using peptide sequences that matched with Q98V90, E2 protein using peptide sequences that matched with D1KSI0, p7 protein using peptide sequences that matched with D3W7L4, NS2 protein using peptide sequences that matched with F4YQP9, NS3 protein using peptide sequences that matched with F8SI75, F6L9J4, A3EZJ3 and K7Y470, NS4A protein using peptide sequences that matched with K7Y470, NS4B protein using peptide sequences that matched with C9WV93 and Q68586, NS5A protein using peptide sequences that matched with Q6TZ17, Q1HFC6, A9JKP2, A9JKN5 and M9UX90 and NS5B protein using peptide sequences that matched with M9UX90, C7SCB7, G8CSB7, E7BK75 and Q81598 to obtained the RHCV final consensus sequence. Moreover, to obtain the HHCV final consensus sequence, core protein (F protein included) consensus sequence was constructed using recovered peptide sequences that matched with B3TL57 and C0SUM8, E1 protein using peptide sequences that matched with B6USQ0, E2 protein using peptide sequences that matched with E9LLA5, p7 protein using peptide sequences that matched with E9LLA5, NS2 protein using peptide sequences that matched with E9LLA5, NS3 protein using peptide sequences that matched with D2JVF5, J7HHX1 and K7XN61, NS4A protein using peptide sequences that matched with Q81592, NS4B protein using peptide sequences that matched with Q81592, NS5A protein using peptide sequences that matched with Q1HFF4, Q1HFF3, A4UXV5 and Q1HFG0 and NS5B protein using peptide sequences that matched with I0J2K8. For more detailed information consult Table 1, Supplementary Table S2 and S3. The consensus sequences constructed for each specific HCV-like particle proteins relatively for each animal and the final constructed consensus sequence of HCV-like viruses obtained for each animal were used to perform blastp in the UniProt database (http://www.uniprot.org/blast/). The final constructed amino-acid sequences of RHCV and HHCV have been deposited in the ProteomeXchange Consortium via the PRIDE (http://www.ebi.ac.uk/pride/archive/) partner repository with the dataset identifier PXD001992.

### Phylogenetic analysis

The amino acids sequences of draw almost complete RHCV and HHCV polyproteins obtained from complete sequences and consensus sequences construction of all peptide sequences recovered from *de novo* inoculated naïve MDBK cells by MALDI TOF/TOF-MS/MS procedures with significant protein scores (P.S.) to HCV specific proteins, were aligned using Clustal W through MEGA version 5.05^31^,^32^ for phylogenetic inference. An evolutionary history was inferred using the Neighbor-Joining (NJ) method^33^. The percentage of replicate trees in which the associated taxa clustered together in the bootstrap test (1,000 replicates) are shown next to the branches^34^. The tree is drawn to scale, with branch lengths in the same units as those of the evolutionary distances used to infer the phylogenetic tree. The evolutionary distances were computed using the JTT matrix-based method^35^ and are in the units of the number of amino-acid substitutions per site. The rate variation among sites was modeled with a gamma distribution (shape parameter = 5). The analysis involved 23 amino-acid sequences. All positions containing gaps and missing data were eliminated. There were a total of 1,554 positions in the final dataset and the evolutionary analyses were conducted in MEGA version 5.05^32^. An evolutionary history using the Maximum Likelihood (ML) method was also inferred. The percentage of replicate trees in which the associated taxa clustered together in the bootstrap test (1,000 replicates) is shown next to the branches^34^. The tree is drawn to scale, with branch lengths in the same units as those of the evolutionary distances used to infer the phylogenetic tree. The evolutionary distances were computed using the Whelan and Goldman method^36^ and are in the units of the number of amino-acid substitutions per site. The analysis involved 23 amino-acid sequences. All positions containing gaps and missing data were eliminated. There were a total of 1,554 positions in the final dataset and evolutionary analyses were conducted in MEGA version 5.05^32^. For a precise classification of the first rabbit and first hare HCV-like viruses, the complete coding sequences (CDS) of 16 HCV genotypes/subtypes sequences deposited at GenBank, NCBI (http://www.ncbi.nlm.nih.gov/) database, as summarized in Supplementary Table S4, were included in the phylogenetic analysis. In addition, CDS sequences of hepaciviruses (HCV-like viruses) achieved in animal species (dog, horse, *Peromyscus maniculatus* (rodent), *Hipposideros vittatus* (bat) and black-and-white *Colobus* were included (Supplementary Table S4).

## Additional Information

**How to cite this article**: Silva, E. *et al.* Hepatitis C-like viruses are produced in cells from rabbit and hare DNA. *Sci. Rep.* **5**, 14535; doi: 10.1038/srep14535 (2015).

## Supplementary Material

Supplementary Information

## Figures and Tables

**Table 1:** MALDI-TOF/TOF-MS/MS mass spectrometry.

**Animal**	**UniProt accession no.**	**HCV Protein (genotype)**	**Mass (Da)**	**Protein score**	**Expect-value**	**N° of peptide sequence matches**
DR	B3TKW7	F (1a)	17,015	85	0.00039	51
DR	F4YQ96	Core	21,004	78	0.0019	61
DR	B8QB25	Core	15,078	65	0.13	38
DR	F1A6I7	Core/E1 (1b)	20,996	95	4.4e-005	59
DR	Q98V90	Core/E1/E2	36,052	90	0.00012	83
DR	D1KSI0	E2 (3a)	41,959	86	0.00032	86
DR	D3W7L4	E2/p7/NS2/NS3 (1a)	54,879	82	0.00078	96
DR	F4YQP9	NS2	24,245	69	0.015	74
DR	F8SI75	NS3	19,174	112	8e-007	60
DR	F6L9J4	NS3	17,754	87	0.00027	57
DR	A3EZJ3	NS3	68,216	81	0.001	94
DR	K7Y470	NS3-4A	75,184	94	4.6e-005	99
DR	C9WV93	NS4B	27,686	97	2.5e-005	91
DR	Q68586	NS4B	20,132	82	0.00077	78
DR	Q6TZ17	NS5A	27,433	77	0.0026	78
DR	Q1HFC6	NS5A	49,560	73	0.0061	87
DR	A9JKP2	NS5A	50,120	72	0.008	85
DR	A9JKN5	NS5A	50,064	68	0.02	88
DR	M9UX90	NS5A/NS5B (3b)	116,298	68	0.019	76
DR	C7SCB7	NS5B	163,51	86	0.00031	72
DR	G8CSB7	NS5B	23,529	86	0.00028	75
DR	E7BK75	NS5B (3b)	32,573	69	0.016	53
DR	Q81598	NS5B	40,858	65	0.07	53
H	B3TL57	F (1a)	16,955	74	0.0053	44
H	C0SUM8	Core (1b)	46,934	77	0.0027	31
H	B6USQ0	E1	21,278	70	0.011	40
H	E9LLA5	E2/p7/NS2-3	63,556	65	0.084	75
H	D2JVF5	NS3	21,837	76	0.003	51
H	J7HHX1	NS3 (3)	15,091	70	0.012	32
H	K7XN61	NS3-4A	75,514	83	0.0007	79
H	Q81592	NS4A/NS4B	13,899	65	0.14	30
H	Q1HFF4	NS5A	48,917	68	0.019	56
H	Q1HFF3	NS5A	48,940	67	0.025	52
H	A4UXV5	NS5A (1b)	49,355	65	0.052	56
H	Q1HFG0	NS5A	49,009	65	0.047	57
H	I0J2K8	NS5B	12,668	76	0.0032	41

MS/MS peptide sequences summary reported by Mascot version 2.4 of *de novo* inoculated new naïve MDBK cells using supernatant of first inoculated naïve MDBK cells with rabbit and hare DNA samples, 7 days post inoculation. Protein score > 64 are significant (p < 0.05).

DR - Domestic rabbit (Oryctolagus cuniculus), H - Hare (Lepus europaeus).
