# Supplementary material for: Hepatitis C-like viruses are produced in cells from rabbit and hare DNA
Source: Sci Rep. 2015 Sep 29;5:14535. doi: 10.1038/srep14535 (PMC4586723; doi:10.1038/srep14535)
Supplement: Supplementary Information [file srep14535-s1.doc]

**Hepatitis C virus-like are produced in cells from rabbit and hare DNA**

Eliane Silva^1,2^, Hugo Osório^3,4,5^, Gertrude Thompson^1,2^*

^1^Department of Veterinary Clinics, Instituto de Ciências Biomédicas de Abel Salazar da Uiversidade do Porto, Rua de Jorge Viterbo Ferreira, 228, 4050-313 Porto, Portugal. ^2^Centro de Investigação em Biodiversidade e Recursos Genéticos (CIBIO), Research Network in Biodiversity and Evolutionary Biology (InBio), Universidade do Porto, Rua Padre Armando Quintas, 7, 4485-661 Vairão, Portugal. ^3^Instituto de Investigação e Inovação em Saúde, Universidade do Porto, Rua Alfredo Allen, s/n, 4200-135 Porto, Portuga. ^4^Institute of Molecular Pathology and Immunology of the University of Porto (IPATIMUP), Rua Dr. Roberto Frias, S/N, 4200-465 Porto, Portugal. ^5^Faculty of Medicine da University of Porto, Al. Prof. Hernâni Monteiro, 4200-319 Porto, Portugal.


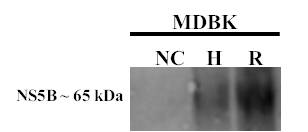


**Supplementary Figure S1.** **Western blot analysis of HCV NS5B protein expression in *de novo* inoculated MDBK cell line**. The HCV NS5B protein (~ 65 kDa) was detected in MDBK cells harbouring the rabbit and hare samples. (H) *De novo* inoculated new naïve MDBK cells using supernatant of first inoculated naïve MDBK cells with hare DNA sample, (R) *De novo* inoculated new naïve MDBK cells using supernatant of first cultures of rabbit DNA in naïve MDBK cells and (NC) Uninoculated MDBK cells (negative control).

**a**

**b**

**c**

**d**

**e**

**f**

**g**

**h**

**Supplementary Figure S2.** **MS/MS spectrum**. **MS/MS spectra of the peptide sequences identified in** recovered supernatant of *de novo* inoculated new naïve MDBK cells using supernatant of first cultures of rabbit and hare DNA samples in naïve MDBK cells,7 days post inoculation. P.S. > 64 are significant (p<0.05). The A, B, C and D MS spectra in reflector mode relatively to the identified peptide sequences that matched with B3TKW7, F4YQ96, B8QB25, F1A6I7, Q98V90, D1KSI0, D3W7L4, F4YQP9, A3EZJ3, F8SI75, F6L9J4, K7Y470, Q68586, C9WV93, A9JKN5, A9JKP2, Q6TZ17, Q1HFC6, M9UX90, G8CSB7, Q81598, C7SCB7, E7BK75 (UniProt accession nos.), from the rabbit sample. The E, F, G, and H MS spectra in reflector mode relatively to the identified peptide sequences that matched with **B3TL57, C0SUM8,** B6USQ0, E9LLA5, J7HHX1, D2JVF5, K7XN61, Q81592, Q1HFF3, Q1HFF4, Q1HFG0, A4UXV5, I0J2K8 (UniProt accession nos.) from the hare sample. Consult Supplementary Table S1 and S2 for more detailed information.

**Supplementary Table S1. DNA, RNA and protein concentrations**. DNA, RNA and protein concentrations were measured after samples DNA extraction.

| **Animal** | **Concentration after RNAse A treatment** | | |
| --- | --- | --- | --- |
|  | **DNA (ng ml^-1^)** | **RNA (ng ml^-1^)** | **Potein (µg ml^-1^)** |
| DR | 2,500 | < 20 | ˃ 26 |
| H | 640 | < 20 | < 1 |

DR - Domestic rabbit *(Oryctolagus cuniculus)*, H - Hare *(Lepus europaeus)*.

**Supplementary Table S2.** **Rabbit MS/MS peptides sequence report.** MS/MS peptide sequences reported by Mascot version 2.4 from *de novo* inoculated fresh naïve MDBK cells using supernatant of first inoculated naïve MDBK cells with rabbit DNA sample, 7 days post inoculation. P.S. > 64 are significant (p<0.05).

| **UniProt accession no.. (genotype)** | **HCV fragment (genotype)** | **Calculated mass (Da)** | **Genome position (amino-acid)** | **Protein score** | **Expect-value** | **Peptide sequence** |
| --- | --- | --- | --- | --- | --- | --- |
| B3TKW7 | F protein (1a) | 1073,5288 | 1 – 9 | 85 | 0.00039 | MSTNPKPQR** |
| B3TKW7 | F protein (1a) | 841,4770 | 7 - 13 | 85 | 0.00039 | PQRKTNV |
| B3TKW7 | F protein (1a) | 1296,7262 | 16 - 27 | 85 | 0.00039 | TVAHRTLSSRVA |
| B3TKW7 | F protein (1a) | 2210,1048 | 23 - 42 | 85 | 0.00039 | SSRVAARSLVEFTCCRAGAL |
| B3TKW7 | F protein (1a) | 2325,1318 | 23 - 43 | 85 | 0.00039 | SSRVAARSLVEFTCCRAGALD |
| B3TKW7 | F protein (1a) | 2582,2417 | 29 - 49 | 85 | 0.00039 | RSLVEFTCCRAGALDWVCARR |
| B3TKW7 | F protein (1a) | 2565,2264 | 34 - 55 | 85 | 0.00039 | FTCCRAGALDWVCARRGRLPSG |
| B3TKW7 | F protein (1a) | 1046,4968 | 37 - 45 | 85 | 0.00039 | CRAGALDWV |
| B3TKW7 | F protein (1a) | 1802,8893 | 37 - 51 | 85 | 0.00039 | CRAGALDWVCARRGR |
| B3TKW7 | F protein (1a) | 1170,6404 | 45 - 54 | 85 | 0.00039 | VCARRGRLPS |
| B3TKW7 | F protein (1a) | 1040,5727 | 51 - 59 | 85 | 0.00039 | RLPSGRNLE |
| B3TKW7 | F protein (1a) | 1197,6353 | 52 - 62 | 85 | 0.00039 | LPSGRNLEVDV |
| B3TKW7 | F protein (1a) | 844,4178 | 59 - 66 | 85 | 0.00039 | EVDVSLSP |
| B3TKW7 | F protein (1a) | 1871,0490 | 62 - 80 | 85 | 0.00039 | VSLSPRRVGPRVGPGPSPG |
| B3TKW7 | F protein (1a) | 714,4137 | 63 - 68 | 85 | 0.00039 | SLSPRR |
| B3TKW7 | F protein (1a) | 2208,1256 | 68 - 89 | 85 | 0.00039 | RVGPRVGPGPSPGTLGPSMAMR** |
| B3TKW7 | F protein (1a) | 2107,0667 | 69 - 90 | 85 | 0.00039 | VGPRVGPGPSPGTLGPSMAMRA** |
| B3TKW7 | F protein (1a) | 1952,9561 | 70 - 89 | 85 | 0.00039 | GPRVGPGPSPGTLGPSMAMR** |
| B3TKW7 | F protein (1a) | 2383,1485 | 72 - 96 | 85 | 0.00039 | RVGPGPSPGTLGPSMAMRAAGGRDG** |
| B3TKW7 | F protein (1a) | 1614,7494 | 74 - 90 | 85 | 0.00039 | GPGPSPGTLGPSMAMRA** |
| B3TKW7 | F protein (1a) | 2157,9895 | 75 - 97 | 85 | 0.00039 | PGPSPGTLGPSMAMRAAGGRDGS** |
| B3TKW7 | F protein (1a) | 1515,7174 | 77 - 92 | 85 | 0.00039 | PSPGTLGPSMAMRAAG** |
| B3TKW7 | F protein (1a) | 2234,0606 | 81 - 102 | 85 | 0.00039 | TLGPSMAMRAAGGRDGSCLPVV** |
| B3TKW7 | F protein (1a) | 1273,6383 | 82 - 94 | 85 | 0.00039 | LGPSMAMRAAGGR |
| B3TKW7 | F protein (1a) | 1789,8022 | 83 - 100 | 85 | 0.00039 | GPSMAMRAAGGRDGSCLP |
| B3TKW7 | F protein (1a) | 2214,1071 | 84 - 105 | 85 | 0.00039 | PSMAMRAAGGRDGSCLPVVLGL |
| B3TKW7 | F protein (1a) | 1978,9387 | 85 - 103 | 85 | 0.00039 | SMAMRAAGGRDGSCLPVVL** |
| B3TKW7 | F protein (1a) | 1516,7239 | 87 - 101 | 85 | 0.00039 | AMRAAGGRDGSCLPV |
| B3TKW7 | F protein (1a) | 1540,8032 | 91 - 106 | 85 | 0.00039 | AGGRDGSCLPVVLGLA |
| B3TKW7 | F protein (1a) | 2093,1303 | 91 - 112 | 85 | 0.00039 | AGGRDGSCLPVVLGLAGALLTP |
| B3TKW7 | F protein (1a) | 1114,5805 | 94 - 103 | 85 | 0.00039 | RDGSCLPVVL |
| B3TKW7 | F protein (1a) | 1958,9962 | 117 - 134 | 85 | 0.00039 | ATWVRSSIPSRAASPTSW |
| B3TKW7 | F protein (1a) | 1031,5400 | 118 - 126 | 85 | 0.00039 | TWVRSSIPS |
| B3TKW7 | F protein (1a) | 1129,6203 | 120 - 130 | 85 | 0.00039 | VRSSIPSRAAS |
| B3TKW7 | F protein (1a) | 1226,6731 | 120 - 131 | 85 | 0.00039 | VRSSIPSRAASP |
| B3TKW7 | F protein (1a) | 1501,7637 | 121 - 134 | 85 | 0.00039 | RSSIPSRAASPTSW |
| B3TKW7 | F protein (1a) | 2115,0709 | 123 - 143 | 85 | 0.00039 | SIPSRAASPTSWGTSRSSAPL |
| B3TKW7 | F protein (1a) | 1216,5836 | 125 - 136 | 85 | 0.00039 | PSRAASPTSWGT |
| B3TKW7 | F protein (1a) | 876,3978 | 128 - 136 | 85 | 0.00039 | AASPTSWGT |
| B3TKW7 | F protein (1a) | 2084,0538 | 129 - 149 | 85 | 0.00039 | ASPTSWGTSRSSAPLLEALPG |
| B3TKW7 | F protein (1a) | 2367,1859 | 129 - 151 | 85 | 0.00039 | ASPTSWGTSRSSAPLLEALPGPW |
| B3TKW7 | F protein (1a) | 1232,5786 | 131 - 142 | 85 | 0.00039 | PTSWGTSRSSAP |
| B3TKW7 | F protein (1a) | 2583,2904 | 131 - 154 | 85 | 0.00039 | PTSWGTSRSSAPLLEALPGPWRMA** |
| B3TKW7 | F protein (1a) | 1147,5622 | 133 - 143 | 85 | 0.00039 | SWGTSRSSAPL |
| B3TKW7 | F protein (1a) | 2282,1630 | 134 - 154 | 85 | 0.00039 | WGTSRSSAPLLEALPGPWRMA |
| B3TKW7 | F protein (1a) | 2126,0942 | 136 - 155 | 85 | 0.00039 | TSRSSAPLLEALPGPWRMAS |
| B3TKW7 | F protein (1a) | 1249,6707 | 140 - 151 | 85 | 0.00039 | SAPLLEALPGPW |
| B3TKW7 | F protein (1a) | 1961,9822 | 144 - 160 | 85 | 0.00039 | LEALPGPWRMASGFWKT** |
| B3TKW7 | F protein (1a) | 1142,5542 | 145 - 154 | 85 | 0.00039 | EALPGPWRMA** |
| B3TKW7 | F protein (1a) | 1832,9032 | 145 - 160 | 85 | 0.00039 | EALPGPWRMASGFWKT |
| B3TKW7 | F protein (1a) | 1098,5280 | 152 - 160 | 85 | 0.00039 | RMASGFWKT** |
| F4YQ96 | Core protein | 912,5002 | 13 - 19 | 78 | 0.0019 | RNTNRRP |
| F4YQ96 | Core protein | 1098,5530 | 14 - 22 | 78 | 0.0019 | NTNRRPQDV |
| F4YQ96 | Core protein | 1226,6480 | 14 - 23 | 78 | 0.0019 | NTNRRPQDVK |
| F4YQ96 | Core protein | 784,3940 | 16 - 21 | 78 | 0.0019 | NRRPQD |
| F4YQ96 | Core protein | 1044,5829 | 17 - 24 | 78 | 0.0019 | RRPQDVKF |
| F4YQ96 | Core protein | 829,4334 | 19 - 25 | 78 | 0.0019 | PQDVKFP |
| F4YQ96 | Core protein | 1454,7518 | 19 - 33 | 78 | 0.0019 | PQDVKFPGGGQIVGG |
| F4YQ96 | Core protein | 1031,5036 | 20 - 29 | 78 | 0.0019 | QDVKFPGGGQ |
| F4YQ96 | Core protein | 1277,6768 | 23 - 35 | 78 | 0.0019 | KFPGGGQIVGGVY |
| F4YQ96 | Core protein | 1183,6826 | 32 - 42 | 78 | 0.0019 | GGVYLLPRRGP |
| F4YQ96 | Core protein | 899,5665 | 44 - 51 | 78 | 0.0019 | LGVRATRK |
| F4YQ96 | Core protein | 1952,9758 | 69 - 84 | 78 | 0.0019 | RRPEGRTWAQPGYPWP |
| F4YQ96 | Core protein | 1131,5461 | 73 - 82 | 78 | 0.0019 | GRTWAQPGYP |
| F4YQ96 | Core protein | 1607,7079 | 78 - 91 | 78 | 0.0019 | QPGYPWPLYGNEGM |
| F4YQ96 | Core protein | 1832,8345 | 82 - 97 | 78 | 0.0019 | PWPLYGNEGMGWAGWL |
| F4YQ96 | Core protein | 748,3544 | 83 - 88 | 78 | 0.0019 | WPLYGN |
| F4YQ96 | Core protein | 877,3970 | 83 - 89 | 78 | 0.0019 | WPLYGNE |
| F4YQ96 | Core protein | 2503,2067 | 84 - 106 | 78 | 0.0019 | PLYGNEGMGWAGWLLSPRGSRPS** |
| F4YQ96 | Core protein | 1096,4648 | 85 - 94 | 78 | 0.0019 | LYGNEGMGWA |
| F4YQ96 | Core protein | 2049,9731 | 85 - 103 | 78 | 0.0019 | LYGNEGMGWAGWLLSPRGS |
| F4YQ96 | Core protein | 1789,8206 | 87 - 103 | 78 | 0.0019 | GNEGMGWAGWLLSPRGS** |
| F4YQ96 | Core protein | 708,2537 | 88 - 93 | 78 | 0.0019 | NEGMGW** |
| F4YQ96 | Core protein | 1232,5648 | 88 - 98 | 78 | 0.0019 | NEGMGWAGWLL |
| F4YQ96 | Core protein | 1515,7293 | 89 - 102 | 78 | 0.0019 | EGMGWAGWLLSPRG |
| F4YQ96 | Core protein | 1774,8573 | 89 - 104 | 78 | 0.0019 | EGMGWAGWLLSPRGSR** |
| F4YQ96 | Core protein | 2499,1754 | 89 - 111 | 78 | 0.0019 | EGMGWAGWLLSPRGSRPSWGPTD |
| F4YQ96 | Core protein | 2214,0793 | 91 - 110 | 78 | 0.0019 | MGWAGWLLSPRGSRPSWGPT** |
| F4YQ96 | Core protein | 1594,8368 | 96 - 109 | 78 | 0.0019 | WLLSPRGSRPSWGP |
| F4YQ96 | Core protein | 844,4304 | 101 - 107 | 78 | 0.0019 | RGSRPSW |
| F4YQ96 | Core protein | 1467,7331 | 101 - 113 | 78 | 0.0019 | RGSRPSWGPTDPR |
| F4YQ96 | Core protein | 2863,5390 | 101 - 125 | 78 | 0.0019 | RGSRPSWGPTDPRRRSRNLGKVIDT |
| F4YQ96 | Core protein | 2820,5219 | 102 - 126 | 78 | 0.0019 | GSRPSWGPTDPRRRSRNLGKVIDTL |
| F4YQ96 | Core protein | 3024,5788 | 103 - 128 | 78 | 0.0019 | SRPSWGPTDPRRRSRNLGKVIDTLTC |
| F4YQ96 | Core protein | 1011,4774 | 104 - 112 | 78 | 0.0019 | RPSWGPTDP |
| F4YQ96 | Core protein | 1226,6268 | 107 - 116 | 78 | 0.0019 | WGPTDPRRRS |
| F4YQ96 | Core protein | 953,5155 | 108 - 115 | 78 | 0.0019 | GPTDPRRR |
| F4YQ96 | Core protein | 843,4899 | 113 - 118 | 78 | 0.0019 | RRRSRN |
| F4YQ96 | Core protein | 2142,0377 | 123 - 142 | 78 | 0.0019 | IDTLTCGFADLMGYIPLVGA** |
| F4YQ96 | Core protein | 807,3837 | 132 - 138 | 78 | 0.0019 | DLMGYIP |
| F4YQ96 | Core protein | 1244,6475 | 132 - 143 | 78 | 0.0019 | DLMGYIPLVGAP |
| F4YQ96 | Core protein | 1357,7316 | 132 - 144 | 78 | 0.0019 | DLMGYIPLVGAPL |
| F4YQ96 | Core protein | 1129,6205 | 133 - 143 | 78 | 0.0019 | LMGYIPLVGAP |
| F4YQ96 | Core protein | 1469,7952 | 133 - 147 | 78 | 0.0019 | LMGYIPLVGAPLGGP** |
| F4YQ96 | Core protein | 807,4201 | 134 - 140 | 78 | 0.0019 | MGYIPLV** |
| F4YQ96 | Core protein | 919,4837 | 134 - 142 | 78 | 0.0019 | MGYIPLVGA |
| F4YQ96 | Core protein | 1870,9975 | 134 - 152 | 78 | 0.0019 | MGYIPLVGAPLGGEARALA** |
| F4YQ96 | Core protein | 1040,5801 | 139 - 149 | 78 | 0.0019 | LVGAPLGGMAR |
| F4YQ96 | Core protein | 1518,8089 | 139 - 154 | 78 | 0.0019 | LVGAPLGGCARALAHG |
| F4YQ96 | Core protein | 933,5145 | 140 - 149 | 78 | 0.0019 | VGAPLGGHAR |
| F4YQ96 | Core protein | 1597,9165 | 140 - 156 | 78 | 0.0019 | VGAPLGGPARALAHGVR |
| F4YQ96 | Core protein | 2281,1927 | 148 - 169 | 78 | 0.0019 | ARALAHGVRVLEDGVNYATGNV |
| F4YQ96 | Core protein | 1498,7528 | 152 - 165 | 78 | 0.0019 | AHGVRVLEDGVNYA |
| F4YQ96 | Core protein | 2214,0488 | 153 - 173 | 78 | 0.0019 | HGVRVLEDGVNYATGNIPGCS |
| F4YQ96 | Core protein | 1756,7727 | 160 - 176 | 78 | 0.0019 | DGVNYATGNVPGCSFSI |
| F4YQ96 | Core protein | 1976,9376 | 164 - 181 | 78 | 0.0019 | YATGNMPGCSFSIFLLAL** |
| F4YQ96 | Core protein | 1540,7232 | 166 - 179 | 78 | 0.0019 | TGNEPGCSFSIFLL |
| F4YQ96 | Core protein | 1310,6329 | 168 - 179 | 78 | 0.0019 | NGPGCSFSIFLL |
| F4YQ96 | Core protein | 1565,8276 | 168 - 181 | 78 | 0.0019 | NKPGCSFSIFLLAL |
| F4YQ96 | Core protein | 1035,6368 | 174 - 182 | 78 | 0.0019 | FSIFLLALL |
| F4YQ96 | Core protein | 1349,7628 | 175 - 186 | 78 | 0.0019 | SIFLLALLSCLT |
| F4YQ96 | Core protein | 889,4943 | 180 - 187 | 78 | 0.0019 | ALLSCLTI |
| B8QB25 | Core protein | 2124,1810 | 6 - 22 | 65 | 0.13 | KPQRKTKRNTNRRPMDV |
| B8QB25 | Core protein | 2030,0592 | 11 - 28 | 65 | 0.13 | TKRNTNRRPMDVKFPGGG |
| B8QB25 | Core protein | 787,4413 | 12 - 17 | 65 | 0.13 | KRNTNR |
| B8QB25 | Core protein | 912,5002 | 13 - 19 | 65 | 0.13 | RNTNRRP |
| B8QB25 | Core protein | 2214,1440 | 13 - 32 | 65 | 0.13 | RNTNRRPMDVKFPGGGQIVG** |
| B8QB25 | Core protein | 2214,1328 | 14 - 34 | 65 | 0.13 | NTNRRPMDVKFPGGGQIVGGV** |
| B8QB25 | Core protein | 2644,3544 | 14 - 38 | 65 | 0.13 | NTNRRPMDVKFPGGGQIVGGVYLGP** |
| B8QB25 | Core protein | 1604,8563 | 21 - 36 | 65 | 0.13 | DVKFPGGGQIVGGVYL |
| B8QB25 | Core protein | 1376,7453 | 22 - 35 | 65 | 0.13 | VKFPGGGQIVGGVY |
| B8QB25 | Core protein | 1115,5975 | 25 - 36 | 65 | 0.13 | PGGGQIVGGVYL |
| B8QB25 | Core protein | 1147,6026 | 27 - 37 | 65 | 0.13 | GGQIVGGVYLW |
| B8QB25 | Core protein | 1272,7303 | 30 - 41 | 65 | 0.13 | IVGGVYLSPRRG |
| B8QB25 | Core protein | 916,5131 | 31 - 39 | 65 | 0.13 | VGGVYLGPR |
| B8QB25 | Core protein | 1608,9576 | 31 - 45 | 65 | 0.13 | VGGVYLIPRRGPRLG |
| B8QB25 | Core protein | 1226,6884 | 31 - 42 | 65 | 0.13 | VGGVYLGPRRGP |
| B8QB25 | Core protein | 1466,8906 | 40 - 52 | 65 | 0.13 | RGPRLGVRATRKT |
| B8QB25 | Core protein | 1514,8277 | 46 - 58 | 65 | 0.13 | VRATRKTSERSQP |
| B8QB25 | Core protein | 855,4787 | 55 - 61 | 65 | 0.13 | RSQPRGR |
| B8QB25 | Core protein | 2015,1249 | 56 - 73 | 65 | 0.13 | SQPRGRRQPIPKARPSQG |
| B8QB25 | Core protein | 1402,8269 | 57 - 68 | 65 | 0.13 | QPRGRRQPIPKA |
| B8QB25 | Core protein | 1517,9015 | 59 - 71 | 65 | 0.13 | RGRRQPIPKARPS |
| B8QB25 | Core protein | 1969,0983 | 61 - 76 | 65 | 0.13 | RRQPIPKARPSQGRHW |
| B8QB25 | Core protein | 1061,6458 | 62 - 70 | 65 | 0.13 | RQPIPKARP |
| B8QB25 | Core protein | 1148,6778 | 62 - 71 | 65 | 0.13 | RQPIPKARPS |
| B8QB25 | Core protein | 1276,7364 | 62 - 72 | 65 | 0.13 | RQPIPKARPSQ |
| B8QB25 | Core protein | 1998,0772 | 62 - 78 | 65 | 0.13 | RQPIPKARPSQGRHWGQ |
| B8QB25 | Core protein | 1096,5203 | 74 - 82 | 65 | 0.13 | RHWGQPGYP |
| B8QB25 | Core protein | 1976,8734 | 87 - 104 | 65 | 0.13 | GNEGCGWAGWLMSPRGSR |
| B8QB25 | Core protein | 2290,2016 | 101 - 119 | 65 | 0.13 | RGSRPNWGPNDPRRRSRNL |
| B8QB25 | Core protein | 2191,1219 | 102 - 120 | 65 | 0.13 | GSRPNWGPNDPRRRSRNLG |
| B8QB25 | Core protein | 1110,5319 | 106 - 114 | 65 | 0.13 | NWGPNDPRR |
| B8QB25 | Core protein | 1834,0398 | 108 - 123 | 65 | 0.13 | GPNDPRRRSRNLGKVI |
| B8QB25 | Core protein | 843,4899 | 113 - 118 | 65 | 0.13 | RRRSRN |
| B8QB25 | Core protein | 1197,7418 | 114 - 123 | 65 | 0.13 | RRSRNLGKVI |
| B8QB25 | Core protein | 829,4882 | 115 - 121 | 65 | 0.13 | RSRNLGK |
| B8QB25 | Core protein | 1471,8471 | 115 - 127 | 65 | 0.13 | RSRNLGKVIDTLT |
| B8QB25 | Core protein | 877,4215 | 122 - 129 | 65 | 0.13 | VIDTLTCG |
| B8QB25 | Core protein | 1061,5427 | 122 - 131 | 65 | 0.13 | VIDTLTCGLA |
| F1A6I7 | Genome polyprotein – Core protein (1b) | 1724,9535 | 1 – 18 | 95 | 4.4e-005 | LMGYIPLVGAPLGGVPVG** |
| F1A6I7 | Genome polyprotein – Core protein (1b) | 848,4466 | 2 – 9 | 95 | 4.4e-005 | MGYIPLVG |
| F1A6I7 | Genome polyprotein - Core protein (1b) | 1091,6339 | 8 – 19 | 95 | 4.4e-005 | VGAPLGGVPVNL |
| F1A6I7 | Genome polyprotein - Core protein (1b) | 1402,7391 | 8 – 22 | 95 | 4.4e-005 | VGAPLGGVPVCLAHG |
| F1A6I7 | Genome polyprotein - Core protein (1b) | 3112,6306 | 8 – 39 | 95 | 4.4e-005 | VGAPLGGVPVDLAHGVRVLEDGVNYATGNLPG |
| F1A6I7 | Genome polyprotein - Core protein (1b) | 855,4524 | 10 - 18 | 95 | 4.4e-005 | APLGGVPVM** |
| F1A6I7 | Genome polyprotein - Core protein (1b) | 1775,0094 | 11 - 27 | 95 | 4.4e-005 | PLGGVPVYLAHGVRVLE |
| F1A6I7 | Genome polyprotein - Core protein (1b) | 2120,1379 | 12 - 32 | 95 | 4.4e-005 | LGGVPVGLAHGVRVLEDGVNY |
| F1A6I7 | Genome polyprotein - Core protein (1b) | 849,4709 | 13 - 21 | 95 | 4.4e-005 | GGVPVTLAH |
| F1A6I7 | Genome polyprotein - Core protein (1b) | 2478,2867 | 14 - 37 | 95 | 4.4e-005 | GVPVELAHGVRVLEDGVNYATGNL |
| F1A6I7 | Genome polyprotein - Core protein (1b) | 1971,0214 | 16 – 33 | 95 | 4.4e-005 | PVYLAHGVRVLEDGVNYA |
| F1A6I7 | Genome polyprotein - Core protein (1b) | 2658,3224 | 18 – 42 | 95 | 4.4e-005 | ILAHGVRVLEDGVNYATGNLPGCSF |
| F1A6I7 | Genome polyprotein - Core protein (1b) | 1107,6037 | 19 – 28 | 95 | 4.4e-005 | LAHGVRVLED |
| F1A6I7 | Genome polyprotein - Core protein (1b) | 1377,7365 | 19 – 31 | 95 | 4.4e-005 | LAHGVRVLEDGVN |
| F1A6I7 | Genome polyprotein - Core protein (1b) | 2311,1379 | 19 – 40 | 95 | 4.4e-005 | LAHGVRVLEDGVNYATGNLPGC |
| F1A6I7 | Genome polyprotein - Core protein (1b) | 2214,0488 | 21 – 41 | 95 | 4.4e-005 | HGVRVLEDGVNYATGNLPGCS |
| F1A6I7 | Genome polyprotein - Core protein (1b) | 843,4450 | 22 – 29 | 95 | 4.4e-005 | GVRVLEDG |
| F1A6I7 | Genome polyprotein - Core protein (1b) | 2111,9834 | 25 – 44 | 95 | 4.4e-005 | VLEDGVNYATGNLPGCSFSI |
| F1A6I7 | Genome polyprotein - Core protein (1b) | 717,3116 | 34 – 40 | 95 | 4.4e-005 | TGNLPGC |
| F1A6I7 | Genome polyprotein - Core protein (1b) | 1226,6369 | 39 – 49 | 95 | 4.4e-005 | GCSFSIFLLAL |
| F1A6I7 | Genome polyprotein - Core protein (1b) | 1800,9154 | 39 – 54 | 95 | 4.4e-005 | GCSFSIFLLALLSCLT |
| F1A6I7 | Genome polyprotein - Core protein (1b) | 2945,5685 | 46 – 72 | 95 | 4.4e-005 | LLALLSCLTIPASAYEVRNVSGVYHVT |
| F1A6I7 | Genome polyprotein - Core protein (1b) | 831,4160 | 52 – 59 | 95 | 4.4e-005 | CLTIPASA |
| F1A6I7 | Genome polyprotein - E1 protein (1b) | 1575,8257 | 53 – 67 | 95 | 4.4e-005 | LTIPASAYEVRNVSG |
| F1A6I7 | Genome polyprotein - E1 protein (1b) | 1248,6098 | 56 – 67 | 95 | 4.4e-005 | PASAYEVRNVSG |
| F1A6I7 | Genome polyprotein - E1 protein (1b) | 1847,9166 | 56 – 72 | 95 | 4.4e-005 | PASAYEVRNVSGVYHVT |
| F1A6I7 | Genome polyprotein - E1 protein (1b) | 1244,6262 | 63 – 73 | 95 | 4.4e-005 | RNVSGVYHVTN |
| F1A6I7 | Genome polyprotein - E1 protein (1b) | 2187,9379 | 66 – 85 | 95 | 4.4e-005 | SGVYHVTNDCSNSSIVYEAS |
| F1A6I7 | Genome polyprotein - E1 protein (1b) | 2788,1779 | 68 – 91 | 95 | 4.4e-005 | VYHVTNDCSNSSIVYEASDMIMHT** |
| F1A6I7 | Genome polyprotein - E1 protein (1b) | 2451,0029 | 69 – 89 | 95 | 4.4e-005 | YHVTNDCSNSSIVYEASDMIM** |
| F1A6I7 | Genome polyprotein - E1 protein (1b) | 1414,6286 | 76 – 88 | 95 | 4.4e-005 | SNSSIVYEASDMI |
| F1A6I7 | Genome polyprotein - E1 protein (1b) | 1490,6269 | 77 – 89 | 95 | 4.4e-005 | NSSIVYEASDMIM** |
| F1A6I7 | Genome polyprotein - E1 protein (1b) | 2141,9068 | 77 – 95 | 95 | 4.4e-005 | NSSIVYEASDMIMHTPGCV** |
| F1A6I7 | Genome polyprotein - E1 protein (1b) | 975,4266 | 94 - 101 | 95 | 4.4e-005 | CVPCVREG |
| F1A6I7 | Genome polyprotein - E1 protein (1b) | 1789,8352 | 96 - 110 | 95 | 4.4e-005 | PCVREGNSSRCWVAL |
| F1A6I7 | Genome polyprotein - E1 protein (1b) | 2247,0889 | 97 - 116 | 95 | 4.4e-005 | CVREGNSSRCWVALTPTLAA |
| F1A6I7 | Genome polyprotein - E1 protein (1b) | 1475,6827 | 100 - 112 | 95 | 4.4e-005 | EGNSSRCWVALTP |
| F1A6I7 | Genome polyprotein - E1 protein (1b) | 1197,6870 | 107 - 117 | 95 | 4.4e-005 | WVALTPTLAAR |
| F1A6I7 | Genome polyprotein - E1 protein (1b) | 841,5021 | 110 - 117 | 95 | 4.4e-005 | LTPTLAAR |
| F1A6I7 | Genome polyprotein - E1 protein (1b) | 832,4290 | 118 - 125 | 95 | 4.4e-005 | NSSVPTKT |
| F1A6I7 | Genome polyprotein - E1 protein (1b) | 1277,7568 | 125 - 135 | 95 | 4.4e-005 | TIRRHVDLLVG |
| F1A6I7 | Genome polyprotein - E1 protein (1b) | 2036,0084 | 127 - 144 | 95 | 4.4e-005 | RRHVDLLVGAAAFCSAMY |
| F1A6I7 | Genome polyprotein - E1 protein (1b) | 1879,9073 | 128 - 144 | 95 | 4.4e-005 | RHVDLLVGAAAFCSAMY |
| F1A6I7 | Genome polyprotein - E1 protein (1b) | 1423,6840 | 130 - 143 | 95 | 4.4e-005 | VDLLVGAAAFCSAM |
| F1A6I7 | Genome polyprotein - E1 protein (1b) | 1544,7367 | 132 - 146 | 95 | 4.4e-005 | LLVGAAAFCSAMYVG** |
| F1A6I7 | Genome polyprotein - E1 protein (1b) | 1545,7208 | 143 - 156 | 95 | 4.4e-005 | MYVGDLCGSVFLVS |
| F1A6I7 | Genome polyprotein - E1 protein (1b) | 1540,7596 | 146 - 159 | 95 | 4.4e-005 | GDLCGSVFLVSQLF |
| F1A6I7 | Genome polyprotein - E1 protein (1b) | 1731,8542 | 147 - 161 | 95 | 4.4e-005 | DLCGSVFLVSQLFTF |
| F1A6I7 | Genome polyprotein - E1 protein (1b) | 1108,5587 | 149 - 158 | 95 | 4.4e-005 | CGSVFLVSQL |
| F1A6I7 | Genome polyprotein - E1 protein (1b) | 2137,0891 | 149 - 166 | 95 | 4.4e-005 | CGSVFLVSQLFTFSPRRH |
| F1A6I7 | Genome polyprotein - E1 protein (1b) | 1916,9857 | 156 - 171 | 95 | 4.4e-005 | SQLFTFSPRRHVTVQD |
| F1A6I7 | Genome polyprotein - E1 protein (1b) | 1714,9267 | 157 - 170 | 95 | 4.4e-005 | QLFTFSPRRHVTVQ |
| F1A6I7 | Genome polyprotein - E1 protein (1b) | 1614,7685 | 161 - 173 | 95 | 4.4e-005 | FSPRRHVTVQDCN |
| F1A6I7 | Genome polyprotein - E1 protein (1b) | 851,4726 | 162 - 168 | 95 | 4.4e-005 | SPRRHVT |
| F1A6I7 | Genome polyprotein - E1 protein (1b) | 1540,6987 | 163 - 174 | 95 | 4.4e-005 | PRRHVTVQDCNC |
| F1A6I7 | Genome polyprotein - E1 protein (1b) | 2765,2809 | 164 - 186 | 95 | 4.4e-005 | RRHVTVQDCNCSLYPGHVSGHRM |
| F1A6I7 | Genome polyprotein - E1 protein (1b) | 2609,1798 | 165 - 186 | 95 | 4.4e-005 | RHVTVQDCNCSLYPGHVSGHRM |
| F1A6I7 | Genome polyprotein - E1 protein (1b) | 1121,5730 | 176 - 185 | 95 | 4.4e-005 | LYPGHVSGHR |
| F1A6I7 | Genome polyprotein - E1 protein (1b) | 1047,5032 | 178 - 187 | 95 | 4.4e-005 | PGHVSGHRMA |
| Q98V90 | Genome polyprotein - Core/E1 proteins | 2036,0361 | 4 – 22 | 90 | 0.00012 | ALLSCLTVPASAYQVRNSS |
| Q98V90 | Genome polyprotein - Core/E1 proteins | 1108,5223 | 8 – 17 | 90 | 0.00012 | CLTVPASAYQ |
| Q98V90 | Genome polyprotein - Core/E1 proteins | 1047,5601 | 9 – 18 | 90 | 0.00012 | LTVPASAYQV |
| Q98V90 | Genome polyprotein - Core/E1 proteins | 1548,7896 | 10 - 24 | 90 | 0.00012 | TVPASAYQVRNSSGI |
| Q98V90 | Genome polyprotein - Core/E1 proteins | 1414,6841 | 13 - 25 | 90 | 0.00012 | ASAYQVRNSSGIY |
| Q98V90 | Genome polyprotein - Core/E1 proteins | 1650,8114 | 13 - 27 | 90 | 0.00012 | ASAYQVRNSSGIYHV |
| Q98V90 | Genome polyprotein - E1 protein | 1185,5778 | 16 - 25 | 90 | 0.00012 | YQVRNSSGIY |
| Q98V90 | Genome polyprotein - E1 protein | 2247,0338 | 17 - 36 | 90 | 0.00012 | QVRNSSGIYHVTNDCPNSSI |
| Q98V90 | Genome polyprotein - E1 protein | 705,2388 | 29 - 34 | 90 | 0.00012 | NDCPNS |
| Q98V90 | Genome polyprotein - E1 protein | 1544,7722 | 35 - 48 | 90 | 0.00012 | SIVYETADTILHSP |
| Q98V90 | Genome polyprotein - E1 protein | 1870,9135 | 36 - 52 | 90 | 0.00012 | IVYETADTILHSPGCVP |
| Q98V90 | Genome polyprotein - E1 protein | 2229,1035 | 51 - 70 | 90 | 0.00012 | VPCVREGNTSKCWVAVAPTV |
| Q98V90 | Genome polyprotein - E1 protein | 1575,7828 | 54 - 67 | 90 | 0.00012 | VREGNTSKCWVAVA |
| Q98V90 | Genome polyprotein - E1 protein | 1789,8669 | 56 - 72 | 90 | 0.00012 | EGNTSKCWVAVAPTVAT |
| Q98V90 | Genome polyprotein - E1 protein | 2658,3258 | 56 - 80 | 90 | 0.00012 | EGNTSKCWVAVAPTVATRDGKLPAM |
| Q98V90 | Genome polyprotein - E1 protein | 2117,0688 | 57 - 76 | 90 | 0.00012 | GNTSKCWVAVAPTVATRDGK |
| Q98V90 | Genome polyprotein - E1 protein | 848,4215 | 60 - 66 | 90 | 0.00012 | LPAMQLR |
| Q98V90 | Genome polyprotein - E1 protein | 841,4698 | 63 - 70 | 90 | 0.00012 | WVAVAPTV |
| Q98V90 | Genome polyprotein - E1 protein | 1953,0717 | 64 - 82 | 90 | 0.00012 | VAVAPTVATRDGKLPAMQL** |
| Q98V90 | Genome polyprotein - E1 protein | 1724,9243 | 65 - 81 | 90 | 0.00012 | AVAPTVATRDGKLPAMQ |
| Q98V90 | Genome polyprotein - E1 protein | 1402,7238 | 69 - 81 | 90 | 0.00012 | TVATRDGKLPAMQ** |
| Q98V90 | Genome polyprotein - E1 protein | 1157,6227 | 70 - 80 | 90 | 0.00012 | VATRDGKLPAM |
| Q98V90 | Genome polyprotein - E1 protein | 1244,6547 | 72 - 82 | 90 | 0.00012 | TRDGKLPAMQL** |
| Q98V90 | Genome polyprotein - E1 protein | 1030,5229 | 73 - 81 | 90 | 0.00012 | RDGKLPAMQ** |
| Q98V90 | Genome polyprotein - E1 protein | 1804,9842 | 73 - 87 | 90 | 0.00012 | RDGKLPAMQLRRHID |
| Q98V90 | Genome polyprotein - E1 protein | 843,4636 | 77 - 83 | 90 | 0.00012 | LPAMQLR** |
| Q98V90 | Genome polyprotein - E1 protein | 2137,1830 | 77 - 95 | 90 | 0.00012 | LPAMQLRRHIDLFVGSATL |
| Q98V90 | Genome polyprotein - E1 protein | 714,3847 | 78 - 83 | 90 | 0.00012 | PAMQLR |
| Q98V90 | Genome polyprotein - E1 protein | 1594,8766 | 78 - 90 | 90 | 0.00012 | PAMQLRRHIDLFV |
| Q98V90 | Genome polyprotein - E1 protein | 855,4497 | 80 - 85 | 90 | 0.00012 | MQLRRH** |
| Q98V90 | Genome polyprotein - E1 protein | 1083,5607 | 80 - 87 | 90 | 0.00012 | MQLRRHID** |
| Q98V90 | Genome polyprotein - E1 protein | 1614,7964 | 87 - 101 | 90 | 0.00012 | DLFVGSATLCSALYV |
| Q98V90 | Genome polyprotein - E1 protein | 864,4011 | 90 - 98 | 90 | 0.00012 | VGSATLCSA |
| Q98V90 | Genome polyprotein - E1 protein | 2188,0545 | 92 - 112 | 90 | 0.00012 | SATLCSALYVGDLCGSVFLVG |
| Q98V90 | Genome polyprotein - E1 protein | 2134,0809 | 99 - 117 | 90 | 0.00012 | LYVGDLCGSVFLVGQLFTF |
| Q98V90 | Genome polyprotein - E1 protein | 1107,5965 | 110 - 119 | 90 | 0.00012 | LVGQLFTFSP |
| Q98V90 | Genome polyprotein - E1 protein | 2111,9054 | 115 - 130 | 90 | 0.00012 | FTFSPRRHWTTQDCNC |
| Q98V90 | Genome polyprotein - E1 protein | 1098,5319 | 120 - 127 | 90 | 0.00012 | RRHWTTQD |
| Q98V90 | Genome polyprotein - E1 protein | 2641,1373 | 122 - 143 | 90 | 0.00012 | HWTTQDCNCSIYPGHITGHRMA |
| Q98V90 | Genome polyprotein - E1 protein | 2451,0228 | 135 - 155 | 90 | 0.00012 | GHITGHRMAWDMMMSWSPTTA** |
| Q98V90 | Genome polyprotein - E1 protein | 2945,4424 | 137 - 162 | 90 | 0.00012 | ITGHRMAWDMMMSWSPTTALVVAQLL |
| Q98V90 | Genome polyprotein - E1 protein | 2609,1899 | 140 - 161 | 90 | 0.00012 | HRMAWDMMMSWSPTTALVVAQL** |
| Q98V90 | Genome polyprotein - E1 protein | 2111,9665 | 141 - 158 | 90 | 0.00012 | RMAWDMMMSWSPTTALVV |
| Q98V90 | Genome polyprotein - E1 protein | 2311,0622 | 141 - 160 | 90 | 0.00012 | RMAWDMMMSWSPTTALVVAQ |
| Q98V90 | Genome polyprotein - E1 protein | 2197,0581 | 145 - 163 | 90 | 0.00012 | DMMMSWSPTTALVVAQLLR** |
| Q98V90 | Genome polyprotein - E1 protein | 2050,0414 | 146 - 163 | 90 | 0.00012 | MMMSWSPTTALVVAQLLR** |
| Q98V90 | Genome polyprotein - E1 protein | 1665,8106 | 147 - 161 | 90 | 0.00012 | MMSWSPTTALVVAQL** |
| Q98V90 | Genome polyprotein - E1 protein | 1091,5321 | 148 - 157 | 90 | 0.00012 | MSWSPTTALV |
| Q98V90 | Genome polyprotein - E1 protein | 1206,5955 | 148 - 158 | 90 | 0.00012 | MSWSPTTALVV** |
| Q98V90 | Genome polyprotein - E1 protein | 1565,8963 | 158 - 171 | 90 | 0.00012 | VAQLLRVPQAILDM |
| Q98V90 | Genome polyprotein - E1 protein | 2428,3413 | 160 - 182 | 90 | 0.00012 | QLLRVPQAILDMIAGAHWGVLAG |
| Q98V90 | Genome polyprotein - E1 protein | 1467,8483 | 161 - 173 | 90 | 0.00012 | LLRVPQAILDMIA** |
| Q98V90 | Genome polyprotein - E1 protein | 1197,6427 | 164 - 175 | 90 | 0.00012 | VPQAILDMIAGA |
| Q98V90 | Genome polyprotein - E1 protein | 2428,2613 | 164 - 186 | 90 | 0.00012 | VPQAILDMIAGAHWGVLAGIAYF** |
| Q98V90 | Genome polyprotein - E1 protein | 2478,2440 | 167 - 190 | 90 | 0.00012 | AILDMIAGAHWGVLAGIAYFSMVG** |
| Q98V90 | Genome polyprotein - E1 protein | 2120,0765 | 168 - 187 | 90 | 0.00012 | ILDMIAGAHWGVLAGIAYF** |
| Q98V90 | Genome polyprotein - E1 protein | 851,4654 | 176 - 183 | 90 | 0.00012 | HWGVLAGI |
| Q98V90 | Genome polyprotein - E1 protein | 1565,7701 | 176 - 189 | 90 | 0.00012 | HWGVLAGIAYFSMV** |
| Q98V90 | Genome polyprotein - E1 protein | 1226,6369 | 178 - 189 | 90 | 0.00012 | GVLAGIAYFSMV |
| Q98V90 | Genome polyprotein - E1 protein | 2209,1969 | 178 - 198 | 90 | 0.00012 | GVLAGIAYFSMVGNWAKVLVV** |
| Q98V90 | Genome polyprotein - E1 protein | 1143,5634 | 180 - 190 | 90 | 0.00012 | LAGIAYFSMVG** |
| Q98V90 | Genome polyprotein - E1 protein | 1698,8804 | 184 - 198 | 90 | 0.00012 | AYFSMVGNWAKVLVV** |
| Q98V90 | Genome polyprotein - E1/E2 proteins | 3112,6155 | 187 - 216 | 90 | 0.00012 | SMVGNWAKVLVVLLLFAGVDAETYTTGGSV** |
| Q98V90 | Genome polyprotein - E1/E2 proteins | 1147,6125 | 199 - 209 | 90 | 0.00012 | LLLFAGVDAET |
| Q98V90 | Genome polyprotein - E2 protein | 1121,5829 | 212 - 223 | 90 | 0.00012 | TGGSVARAAYGL |
| Q98V90 | Genome polyprotein - E2 protein | 1064,5614 | 214 - 224 | 90 | 0.00012 | GSVARAAYGLT |
| Q98V90 | Genome polyprotein - E2 protein | 2945,5359 | 214 - 242 | 90 | 0.00012 | GSVARAAYGLTGLFEPGAKQNIQLINSNG |
| Q98V90 | Genome polyprotein - E2 protein | 1847,9781 | 221 - 237 | 90 | 0.00012 | YGLTGLFEPGAKQNIQL |
| Q98V90 | Genome polyprotein - E2 protein | 832,4331 | 223 - 230 | 90 | 0.00012 | LTGLFEPG |
| Q98V90 | Genome polyprotein - E2 protein | 1490,6824 | 250 - 263 | 90 | 0.00012 | ALNCNASLDTGWVA |
| Q98V90 | Genome polyprotein - E2 protein | 1475,7079 | 253 - 266 | 90 | 0.00012 | CNASLDTGWVAGLI |
| Q98V90 | Genome polyprotein - E2 protein | 2214,0746 | 256 - 275 | 90 | 0.00012 | SLDTGWVAGLIYYHKFNSSG |
| Q98V90 | Genome polyprotein - E2 protein | 1248,6655 | 261 - 270 | 90 | 0.00012 | WVAGLIYYHK |
| Q98V90 | Genome polyprotein - E2 protein | 764,3857 | 264 - 269 | 90 | 0.00012 | GLIYYH |
| Q98V90 | Genome polyprotein - E2 protein | 1423,6588 | 278 - 289 | 90 | 0.00012 | ERMASCKPLADF |
| Q98V90 | Genome polyprotein - E2 protein | 849,4055 | 283 - 289 | 90 | 0.00012 | CKPLADF |
| Q98V90 | Genome polyprotein - E2 protein | 1879,8927 | 283 - 299 | 90 | 0.00012 | CKPLADFAQGWGPISYA |
| Q98V90 | Genome polyprotein - E2 protein | 1762,8315 | 285 - 301 | 90 | 0.00012 | PLADFAQGWGPISYANG |
| Q98V90 | Genome polyprotein - E2 protein | 2765,2081 | 288 - 311 | 90 | 0.00012 | DFAQGWGPISYANGTGPEHRPYCW |
| Q98V90 | Genome polyprotein - E2 protein | 975,4662 | 295 - 304 | 90 | 0.00012 | PISYANGTGP |
| Q98V90 | Genome polyprotein - E2 protein | 1540,6670 | 304 - 314 | 90 | 0.00012 | PEHRPYCWHYP |
| Q98V90 | Genome polyprotein - E2 protein | 1540,6670 | 305 - 315 | 90 | 0.00012 | EHRPYCWHYPP |
| Q98V90 | Genome polyprotein - E2 protein | 1553,8388 | 313 - 326 | 90 | 0.00012 | YPPKPCGIVPARTV |
| D1KSI0 | E2 protein (3a) | 1143,5632 | 1 - 12 | 86 | 0.00032 | STHTIGGSAARS |
| D1KSI0 | E2 protein (3a) | 2209,1240 | 2 - 23 | 86 | 0.00032 | THTIGGSAARSAYGITSLFSRG |
| D1KSI0 | E2 protein (3a) | 1971,0174 | 4 - 23 | 86 | 0.00032 | TIGGSAARSAYGITSLFSRG |
| D1KSI0 | E2 protein (3a) | 1108,5876 | 9 - 19 | 86 | 0.00032 | AARSAYGITSL |
| D1KSI0 | E2 protein (3a) | 2478,3455 | 9 - 31 | 86 | 0.00032 | AARSAYGITSLFSRGARQNLQLV |
| D1KSI0 | E2 protein (3a) | 1044,5128 | 12 - 21 | 86 | 0.00032 | SAYGITSLFS |
| D1KSI0 | E2 protein (3a) | 1917,0180 | 18 - 34 | 86 | 0.00032 | SLFSRGARQNLQLVNTN |
| D1KSI0 | E2 protein (3a) | 1047,5574 | 19 - 27 | 86 | 0.00032 | LFSRGARQN |
| D1KSI0 | E2 protein (3a) | 841,4770 | 24 - 30 | 86 | 0.00032 | ARQNLQL |
| D1KSI0 | E2 protein (3a) | 1665,8335 | 25 - 38 | 86 | 0.00032 | RQNLQLVNTNGSWH |
| D1KSI0 | E2 protein (3a) | 1244,6149 | 27 - 37 | 86 | 0.00032 | NLQLVNTNGSW |
| D1KSI0 | E2 protein (3a) | 2187,9603 | 34 - 53 | 86 | 0.00032 | NGSWHINSTALNCNESINTG |
| D1KSI0 | E2 protein (3a) | 1121,5353 | 47 - 57 | 86 | 0.00032 | NESINTGFIAG |
| D1KSI0 | E2 protein (3a) | 1544,7511 | 47 - 60 | 86 | 0.00032 | NESINTGFIAGLFY |
| D1KSI0 | E2 protein (3a) | 2120,0367 | 47 - 64 | 86 | 0.00032 | NESINTGFIAGLFYYHKF |
| D1KSI0 | E2 protein (3a) | 1377,6969 | 50 - 61 | 86 | 0.00032 | INTGFIAGLFYY |
| D1KSI0 | E2 protein (3a) | 1789,9192 | 50 - 64 | 86 | 0.00032 | INTGFIAGLFYYHKF |
| D1KSI0 | E2 protein (3a) | 1820,8886 | 53 - 68 | 86 | 0.00032 | GFIAGLFYYHKFNSTG |
| D1KSI0 | E2 protein (3a) | 717,3228 | 67 - 72 | 86 | 0.00032 | TGCPQR |
| D1KSI0 | E2 protein (3a) | 1098,5604 | 70 - 78 | 86 | 0.00032 | PQRLNSCKP |
| D1KSI0 | E2 protein (3a) | 1268,6336 | 74 - 83 | 86 | 0.00032 | NSCKPITFFR |
| D1KSI0 | E2 protein (3a) | 764,3891 | 76 - 81 | 86 | 0.00032 | CKPITF |
| D1KSI0 | E2 protein (3a) | 1423,6885 | 80 - 91 | 86 | 0.00032 | TFFRQGWGPLTD |
| D1KSI0 | E2 protein (3a) | 2049,9909 | 80 - 98 | 86 | 0.00032 | TFFRQGWGPLTDANISGPS |
| D1KSI0 | E2 protein (3a) | 1226,5932 | 86 - 97 | 86 | 0.00032 | WGPLTDANISGP |
| D1KSI0 | E2 protein (3a) | 2658,2493 | 112 - 136 | 86 | 0.00032 | CKTVSASSVCGPVYCFTPSPVVVGT |
| D1KSI0 | E2 protein (3a) | 2247,1060 | 128 - 149 | 86 | 0.00032 | TPSPVVVGTTDGKGVPTYTWGE |
| D1KSI0 | E2 protein (3a) | 855,4702 | 129 - 137 | 86 | 0.00032 | PSPVVVGTT |
| D1KSI0 | E2 protein (3a) | 1575,8145 | 130 - 145 | 86 | 0.00032 | SPVVVGTTDGKGVPTY |
| D1KSI0 | E2 protein (3a) | 843,4338 | 131 - 139 | 86 | 0.00032 | PVVVGTTDG |
| D1KSI0 | E2 protein (3a) | 832,4291 | 134 - 142 | 86 | 0.00032 | VGTTDGKGV |
| D1KSI0 | E2 protein (3a) | 1030,5295 | 134 - 144 | 86 | 0.00032 | VGTTDGKGVPT |
| D1KSI0 | E2 protein (3a) | 2111,9284 | 134 - 153 | 86 | 0.00032 | VGTTDGKGVPTYTWGENESD |
| D1KSI0 | E2 protein (3a) | 2141,9793 | 141 - 159 | 86 | 0.00032 | GVPTYTWGENESDVFLLES |
| D1KSI0 | E2 protein (3a) | 1800,9370 | 151 - 166 | 86 | 0.00032 | ESDVFLLESLRPPSGR |
| D1KSI0 | E2 protein (3a) | 1157,6444 | 155 - 164 | 86 | 0.00032 | FLLESLRPPS |
| D1KSI0 | E2 protein (3a) | 2451,0559 | 163 - 183 | 86 | 0.00032 | PSGRWFGCTWMNSTGFVKTCG** |
| D1KSI0 | E2 protein (3a) | 2641,1537 | 179 - 203 | 86 | 0.00032 | VKTCGAPPCNIYGGGGNSQNESDLF |
| D1KSI0 | E2 protein (3a) | 2765,2261 | 204 - 226 | 86 | 0.00032 | CPTDCFRKHPEATYSRCGAGPWL |
| D1KSI0 | E2 protein (3a) | 2609,1904 | 206 - 227 | 86 | 0.00032 | TDCFRKHPEATYSRCGAGPWLT |
| D1KSI0 | E2 protein (3a) | 1147,5774 | 209 - 217 | 86 | 0.00032 | FRKHPEATY |
| D1KSI0 | E2 protein (3a) | 844,4079 | 211 - 217 | 86 | 0.00032 | KHPEATY |
| D1KSI0 | E2 protein (3a) | 2111,9339 | 214 - 231 | 86 | 0.00032 | EATYSRCGAGPWLTPRCM |
| D1KSI0 | E2 protein (3a) | 1762,8066 | 218 - 232 | 86 | 0.00032 | SRCGAGPWLTPRCMV** |
| D1KSI0 | E2 protein (3a) | 2311,0700 | 220 - 238 | 86 | 0.00032 | CGAGPWLTPRCMVDYPYRL |
| D1KSI0 | E2 protein (3a) | 2788,2713 | 228 - 248 | 86 | 0.00032 | PRCMVDYPYRLWHYPCTINFT |
| D1KSI0 | E2 protein (3a) | 975,4273 | 239 - 245 | 86 | 0.00032 | WHYPCTI |
| D1KSI0 | E2 protein (3a) | 2229,1227 | 240 - 257 | 86 | 0.00032 | HYPCTINFTLFKVRMFVG |
| D1KSI0 | E2 protein (3a) | 1402,6955 | 241 - 251 | 86 | 0.00032 | YPCTINFTLFK |
| D1KSI0 | E2 protein (3a) | 1804,9004 | 241 - 254 | 86 | 0.00032 | YPCTINFTLFKVRM** |
| D1KSI0 | E2 protein (3a) | 1774,9263 | 243 - 256 | 86 | 0.00032 | CTINFTLFKVRMFV |
| D1KSI0 | E2 protein (3a) | 1847,9427 | 243 - 257 | 86 | 0.00032 | CTINFTLFKVRMFVG** |
| D1KSI0 | E2 protein (3a) | 2036,0376 | 243 - 259 | 86 | 0.00032 | CTINFTLFKVRMFVGGF |
| D1KSI0 | E2 protein (3a) | 1774,9593 | 245 - 259 | 86 | 0.00032 | INFTLFKVRMFVGGF |
| D1KSI0 | E2 protein (3a) | 2197,1619 | 245 - 262 | 86 | 0.00032 | INFTLFKVRMFVGGFEHR |
| D1KSI0 | E2 protein (3a) | 2117,1033 | 247 - 263 | 86 | 0.00032 | FTLFKVRMFVGGFEHRF |
| D1KSI0 | E2 protein (3a) | 1565,8177 | 249 - 261 | 86 | 0.00032 | LFKVRMFVGGFEH |
| D1KSI0 | E2 protein (3a) | 1870,9301 | 250 - 264 | 86 | 0.00032 | FKVRMFVGGFEHRFD |
| D1KSI0 | E2 protein (3a) | 1650,8651 | 287 - 301 | 86 | 0.00032 | PLLHSTTELAILPCS |
| D1KSI0 | E2 protein (3a) | 1553,8123 | 288 - 301 | 86 | 0.00032 | LLHSTTELAILPCS |
| D1KSI0 | E2 protein (3a) | 2214,1065 | 289 - 308 | 86 | 0.00032 | LHSTTELAILPCSFTPMPAL** |
| D1KSI0 | E2 protein (3a) | 1490,7149 | 294 - 306 | 86 | 0.00032 | ELAILPCSFTPMP** |
| D1KSI0 | E2 protein (3a) | 1545,7571 | 294 - 307 | 86 | 0.00032 | ELAILPCSFTPMPA |
| D1KSI0 | E2 protein (3a) | 1064,5035 | 297 - 305 | 86 | 0.00032 | ILPCSFTPM |
| D1KSI0 | E2 protein (3a) | 3112,6056 | 301 - 328 | 86 | 0.00032 | SFTPMPALSTGLIHLHQNIVDVQYLYGV |
| D1KSI0 | E2 protein (3a) | 1248,6900 | 304 - 315 | 86 | 0.00032 | PMPALSTGLIHL |
| D1KSI0 | E2 protein (3a) | 849,4232 | 318 - 324 | 86 | 0.00032 | NIVDVQY |
| D1KSI0 | E2 protein (3a) | 848,4644 | 319 - 325 | 86 | 0.00032 | IVDVQYL |
| D1KSI0 | E2 protein (3a) | 1614,7964 | 319 - 333 | 86 | 0.00032 | IVDVQYLYGVGSGMV** |
| D1KSI0 | E2 protein (3a) | 1698,8440 | 322 - 337 | 86 | 0.00032 | VQYLYGVGSGMVGWAL |
| D1KSI0 | E2 protein (3a) | 1826,9389 | 322 - 338 | 86 | 0.00032 | VQYLYGVGSGMVGWALK |
| D1KSI0 | E2 protein (3a) | 864,3800 | 327 - 335 | 86 | 0.00032 | GVGSGMVGW** |
| D1KSI0 | E2 protein (3a) | 1475,7231 | 327 - 340 | 86 | 0.00032 | GVGSGMVGWALKWE |
| D1KSI0 | E2 protein (3a) | 2137,1071 | 329 - 347 | 86 | 0.00032 | GSGMVGWALKWEFVVLVFP** |
| D1KSI0 | E2 protein (3a) | 1880,0059 | 331 - 346 | 86 | 0.00032 | GMVGWALKWEFVVLVF |
| D1KSI0 | E2 protein (3a) | 2428,3671 | 333 - 353 | 86 | 0.00032 | VGWALKWEFVVLVFPLLADAR |
| D1KSI0 | E2 protein (3a) | 2428,3671 | 334 - 354 | 86 | 0.00032 | GWALKWEFVVLVFPLLADARV |
| D1KSI0 | E2 protein (3a) | 831,4279 | 335 - 340 | 86 | 0.00032 | WALKWE |
| D1KSI0 | E2 protein (3a) | 851,4793 | 340 - 346 | 86 | 0.00032 | EFVVLVF |
| D1KSI0 | E2 protein (3a) | 2945,5945 | 342 - 367 | 86 | 0.00032 | VVLVFPLLADARVCVALWLMLMVSQA** |
| D1KSI0 | E2 protein (3a) | 2134,1141 | 346 - 363 | 86 | 0.00032 | FPLLADARVCVALWLMLM** |
| D1KSI0 | E2 protein (3a) | 1971,0508 | 347 - 363 | 86 | 0.00032 | PLLADARVCVALWLMLM |
| D1KSI0 | E2 protein (3a) | 1548,7867 | 354 - 366 | 86 | 0.00032 | VCVALWLMLMVSQ |
| D1KSI0 | E2 protein (3a) | 1277,6512 | 357 - 367 | 86 | 0.00032 | ALWLMLMVSQA** |
| D1KSI0 | E2 protein (3a) | 1206,6141 | 358 - 367 | 86 | 0.00032 | LWLMLMVSQA** |
| D1KSI0 | E2 protein (3a) | 1091,5355 | 360 - 369 | 86 | 0.00032 | LMLMVSQAEA |
| D3W7L4 | Polyprotein - E2 protein (1a) | 1724,7875 | 11 - 24 | 82 | 0.00078 | LHCPTDCFRKHPSA |
| D3W7L4 | Polyprotein - E2 protein (1a) | 2111,9088 | 13 - 29 | 82 | 0.00078 | CPTDCFRKHPSASYARC |
| D3W7L4 | Polyprotein - E2 protein (1a) | 1091,5512 | 18 - 26 | 82 | 0.00078 | FRKHPSASY |
| D3W7L4 | Polyprotein - E2 protein (1a) | 1402,6486 | 29 - 40 | 82 | 0.00078 | CGSGPWITPRCL |
| D3W7L4 | Polyprotein - E2 protein (1a) | 1098,5644 | 32 - 40 | 82 | 0.00078 | GPWITPRCL |
| D3W7L4 | Polyprotein - E2 protein (1a) | 1157,6015 | 34 - 42 | 82 | 0.00078 | WITPRCLVN |
| D3W7L4 | Polyprotein - E2 protein (1a) | 2036,0454 | 34 - 48 | 82 | 0.00078 | WITPRCLVNYPYRLW |
| D3W7L4 | Polyprotein - E2 protein (1a) | 2134,0571 | 36 - 51 | 82 | 0.00078 | TPRCLVNYPYRLWHYP |
| D3W7L4 | Polyprotein - E2 protein (1a) | 764,3527 | 39 - 44 | 82 | 0.00078 | CLVNYP |
| D3W7L4 | Polyprotein - E2 protein (1a) | 1244,6124 | 46 - 54 | 82 | 0.00078 | RLWHYPCTI |
| D3W7L4 | Polyprotein - E2 protein (1a) | 1826,8814 | 48 - 61 | 82 | 0.00078 | WHYPCTINYTVFKV |
| D3W7L4 | Polyprotein - E2 protein (1a) | 2247,0969 | 49 - 66 | 82 | 0.00078 | HYPCTINYTVFKVRMYVG |
| D3W7L4 | Polyprotein - E2 protein (1a) | 1083,5965 | 53 - 61 | 82 | 0.00078 | TINYTVFKV |
| D3W7L4 | Polyprotein - E2 protein (1a) | 3112,5375 | 53 - 78 | 82 | 0.00078 | TINYTVFKVRMYVGGVEHRLEAACNW |
| D3W7L4 | Polyprotein - E2 protein (1a) | 1548,7759 | 55 - 67 | 82 | 0.00078 | NYTVFKVRMYVGG** |
| D3W7L4 | Polyprotein - E2 protein (1a) | 851,4687 | 60 - 66 | 82 | 0.00078 | KVRMYVG |
| D3W7L4 | Polyprotein - E2 protein (1a) | 1614,8300 | 61 - 74 | 82 | 0.00078 | VRMYVGGVEHRLEA |
| D3W7L4 | Polyprotein - E2 protein (1a) | 1762,8243 | 62 - 76 | 82 | 0.00078 | RMYVGGVEHRLEAAC** |
| D3W7L4 | Polyprotein - E2 protein (1a) | 2765,2221 | 63 - 85 | 82 | 0.00078 | MYVGGVEHRLEAACNWTRGERCD |
| D3W7L4 | Polyprotein - E2 protein (1a) | 2658,1775 | 70 - 90 | 82 | 0.00078 | HRLEAACNWTRGERCDLEDRD |
| D3W7L4 | Polyprotein - E2 protein (1a) | 1185,6394 | 99 - 108 | 82 | 0.00078 | LTTTQWQVLP |
| D3W7L4 | Polyprotein - E2 protein (1a) | 1030,4906 | 102 - 109 | 82 | 0.00078 | TQWQVLPC |
| D3W7L4 | Polyprotein - E2 protein (1a) | 849,4055 | 105 - 111 | 82 | 0.00078 | QVLPCSF |
| D3W7L4 | Polyprotein - E2 protein (1a) | 1206,6496 | 110 - 121 | 82 | 0.00078 | SFTTLPALSTGL |
| D3W7L4 | Polyprotein - E2 protein (1a) | 1544,8311 | 118 - 131 | 82 | 0.00078 | STGLIHLHQNIVDV |
| D3W7L4 | Polyprotein - E2 protein (1a) | 2137,1320 | 120 - 138 | 82 | 0.00078 | GLIHLHQNIVDVQYLYGVG |
| D3W7L4 | Polyprotein - E2 protein (1a) | 2311,1961 | 120 - 140 | 82 | 0.00078 | GLIHLHQNIVDVQYLYGVGSS |
| D3W7L4 | Polyprotein - E2 protein (1a) | 844,3967 | 133 - 140 | 82 | 0.00078 | YLYGVGSS |
| D3W7L4 | Polyprotein - E2 protein (1a) | 1147,6026 | 139 - 148 | 82 | 0.00078 | SSIASWAIKW |
| D3W7L4 | Polyprotein - E2 protein (1a) | 2478,3708 | 145 - 165 | 82 | 0.00078 | AIKWEYVVLLFLLLADARVCS |
| D3W7L4 | Polyprotein - E2 protein (1a) | 1423,6952 | 155 - 166 | 82 | 0.00078 | FLLLADARVCSC |
| D3W7L4 | Polyprotein - E2 protein (1a) | 864,3946 | 161 - 167 | 82 | 0.00078 | ARVCSCL |
| D3W7L4 | Polyprotein - E2 protein (1a) | 1540,6910 | 164 - 175 | 82 | 0.00078 | CSCLWMMLLISQ |
| D3W7L4 | Polyprotein - E2/p7 proteins (1a) | 1467,7933 | 175 - 188 | 82 | 0.00078 | QAEAALENLVLLNA |
| D3W7L4 | Polyprotein - E2/p7 proteins (1a) | 1197,6605 | 177 - 187 | 82 | 0.00078 | EAALENLVLLN |
| D3W7L4 | Polyprotein - E2/p7 proteins (1a) | 841,4909 | 178 - 185 | 82 | 0.00078 | AALENLVL |
| D3W7L4 | Polyprotein - p7 protein (1a) | 1226,6870 | 179 - 190 | 82 | 0.00078 | ALENLVLLNAAS |
| D3W7L4 | Polyprotein - p7 protein (1a) | 2209,2106 | 179 - 200 | 82 | 0.00078 | ALENLVLLNAASLAGTHGLVSF |
| D3W7L4 | Polyprotein - p7 protein (1a) | 2945,5455 | 191 - 215 | 82 | 0.00078 | LAGTHGLVSFLVFFCFAWYLKGKWV |
| D3W7L4 | Polyprotein - p7 protein (1a) | 2197,1223 | 198 - 214 | 82 | 0.00078 | VSFLVFFCFAWYLKGKW |
| D3W7L4 | Polyprotein - p7 protein (1a) | 2117,0961 | 201 - 217 | 82 | 0.00078 | LVFFCFAWYLKGKWVPG |
| D3W7L4 | Polyprotein - p7 protein (1a) | 975,4313 | 202 - 208 | 82 | 0.00078 | VFFCFAW |
| D3W7L4 | Polyprotein - p7 protein (1a) | 1847,9222 | 203 - 216 | 82 | 0.00078 | FFCFAWYLKGKWVP |
| D3W7L4 | Polyprotein - p7 protein (1a) | 1565,8395 | 208 - 220 | 82 | 0.00078 | WYLKGKWVPGAVY |
| D3W7L4 | Polyprotein - p7 protein (1a) | 1540,7748 | 212 - 225 | 82 | 0.00078 | GKWVPGAVYTIYGM |
| D3W7L4 | Polyprotein - p7 protein (1a) | 1879,9695 | 213 - 228 | 82 | 0.00078 | KWVPGAVYTIYGMWPL |
| D3W7L4 | Polyprotein - p7 protein (1a) | 1565,7952 | 215 - 228 | 82 | 0.00078 | VPGAVYTIYGMWPL |
| D3W7L4 | Polyprotein - p7 protein (1a) | 1953,0474 | 216 - 232 | 82 | 0.00078 | PGAVYTIYGMWPLFLLL |
| D3W7L4 | Polyprotein - p7 protein (1a) | 1475,7159 | 218 - 229 | 82 | 0.00078 | AVYTIYGMWPLF** |
| D3W7L4 | Polyprotein - p7 protein (1a) | 1594,8833 | 221 - 233 | 82 | 0.00078 | TIYGMWPLFLLLL** |
| D3W7L4 | Polyprotein - p7 protein (1a) | 1775,0096 | 222 - 236 | 82 | 0.00078 | IYGMWPLFLLLLALP** |
| D3W7L4 | Polyprotein - p7/protease NS2-3 (1a) | 1047,5827 | 225 - 232 | 82 | 0.00078 | MWPLFLLL** |
| D3W7L4 | Polyprotein - p7/protease NS2-3 (1a) | 1490,7365 | 236 - 249 | 82 | 0.00078 | PQRAYALDTEVAAS |
| D3W7L4 | Polyprotein - p7/protease NS2-3 (1a) | 1650,7672 | 236 - 250 | 82 | 0.00078 | PQRAYALDTEVAASC |
| D3W7L4 | Polyprotein - p7/protease NS2-3 (1a) | 2120,0936 | 238 - 258 | 82 | 0.00078 | RAYALDTEVAASCGGVVLVGL |
| D3W7L4 | Polyprotein - protease NS2-3 (1a) | 1789,8954 | 242 - 259 | 82 | 0.00078 | LDTEVAASCGGVVLVGLM |
| D3W7L4 | Polyprotein - protease NS2-3 (1a) | 1545,7709 | 243 - 258 | 82 | 0.00078 | DTEVAASCGGVVLVGL |
| D3W7L4 | Polyprotein - protease NS2-3 (1a) | 1248,6207 | 247 - 259 | 82 | 0.00078 | AASCGGVVLVGLM** |
| D3W7L4 | Polyprotein - protease NS2-3 (1a) | 1575,8365 | 249 - 264 | 82 | 0.00078 | SCGGVVLVGLMALTLS |
| D3W7L4 | Polyprotein - protease NS2-3 (1a) | 832,4728 | 256 - 263 | 82 | 0.00078 | VGLMALTL** |
| D3W7L4 | Polyprotein - protease NS2-3 (1a) | 1064,5576 | 257 - 266 | 82 | 0.00078 | GLMALTLSPY |
| D3W7L4 | Polyprotein - protease NS2-3 (1a) | 1268,6594 | 275 - 283 | 82 | 0.00078 | LWWLQYFLT |
| D3W7L4 | Polyprotein - protease NS2-3 (1a) | 1871,0417 | 282 - 297 | 82 | 0.00078 | LTRVEAQLHVWVPPLN |
| D3W7L4 | Polyprotein - protease NS2-3 (1a) | 714,4024 | 284 - 289 | 82 | 0.00078 | RVEAQL |
| D3W7L4 | Polyprotein - protease NS2-3 (1a) | 2229,2238 | 295 - 314 | 82 | 0.00078 | PLNVRGGRDAVILLMCIVHP |
| D3W7L4 | Polyprotein - protease NS2-3 (1a) | 1800,9702 | 297 - 312 | 82 | 0.00078 | NVRGGRDAVILLMCIV** |
| D3W7L4 | Polyprotein - protease NS2-3 (1a) | 1665,8695 | 300 - 314 | 82 | 0.00078 | GGRDAVILLMCIVHP** |
| D3W7L4 | Polyprotein - protease NS2-3 (1a) | 855,5178 | 301 - 308 | 82 | 0.00078 | GRDAVILL |
| D3W7L4 | Polyprotein - protease NS2-3 (1a) | 1377,7340 | 344 - 354 | 82 | 0.00078 | FVRVQGPLRFC |
| D3W7L4 | Polyprotein - protease NS2-3 (1a) | 1917,0553 | 345 - 360 | 82 | 0.00078 | VRVQGPLRFCALARKM** |
| D3W7L4 | Polyprotein - protease NS2-3 (1a) | 2428,3096 | 345 - 365 | 82 | 0.00078 | VRVQGPLRFCALARKMIGGHY |
| D3W7L4 | Polyprotein - protease NS2-3 (1a) | 1108,5885 | 353 - 361 | 82 | 0.00078 | FCALARKMI |
| D3W7L4 | Polyprotein - protease NS2-3 (1a) | 848,4360 | 354 - 360 | 82 | 0.00078 | CALARKM |
| D3W7L4 | Polyprotein - protease NS2-3 (1a) | 1774,9366 | 374 - 389 | 82 | 0.00078 | GALTGTYVYNHLTPLR |
| D3W7L4 | Polyprotein - protease NS2-3 (1a) | 1107,5825 | 385 - 393 | 82 | 0.00078 | LTPLRDWAH |
| D3W7L4 | Polyprotein - protease NS2-3 (1a) | 1731,9169 | 387 - 401 | 82 | 0.00078 | PLRDWAHNGLRDLAV |
| D3W7L4 | Polyprotein - protease NS2-3 (1a) | 1804,9696 | 388 - 403 | 82 | 0.00078 | LRDWAHNGLRDLAVAV |
| D3W7L4 | Polyprotein - protease NS2-3 (1a) | 1820,9282 | 389 - 404 | 82 | 0.00078 | RDWAHNGLRDLAVAVE |
| D3W7L4 | Polyprotein - protease NS2-3 (1a) | 1698,9305 | 395 - 410 | 82 | 0.00078 | GLRDLAVAVEPVVFSQ |
| D3W7L4 | Polyprotein - protease NS2-3 (1a) | 2050,0445 | 403 - 420 | 82 | 0.00078 | VEPVVFSQMETKLITWGA** |
| D3W7L4 | Polyprotein - protease NS2-3 (1a) | 2641,2040 | 404 - 427 | 82 | 0.00078 | EPVVFSQMETKLITWGADTAACGD** |
| D3W7L4 | Polyprotein - protease NS2-3 (1a) | 1277,6690 | 405 - 415 | 82 | 0.00078 | PVVFSQMETKL |
| D3W7L4 | Polyprotein - protease NS2-3 (1a) | 2609,2506 | 405 - 428 | 82 | 0.00078 | PVVFSQMETKLITWGADTAACGDI |
| D3W7L4 | Polyprotein - protease NS2-3 (1a) | 2451,1410 | 409 - 431 | 82 | 0.00078 | SQMETKLITWGADTAACGDIING |
| D3W7L4 | Polyprotein - protease NS2-3 (1a) | 2214,0991 | 415 - 436 | 82 | 0.00078 | LITWGADTAACGDIINGLPVSA |
| D3W7L4 | Polyprotein - protease NS2-3 (1a) | 1414,6762 | 421 - 434 | 82 | 0.00078 | DTAACGDIINGLPV |
| D3W7L4 | Polyprotein - protease NS2-3 (1a) | 1143,5594 | 425 - 435 | 82 | 0.00078 | CGDIINGLPVS |
| D3W7L4 | Polyprotein - protease NS2-3 (1a) | 1971,0683 | 435 - 452 | 82 | 0.00078 | SARRGREILLGPADKMVS** |
| D3W7L4 | Polyprotein - protease NS2-3 (1a) | 1553,8712 | 438 - 451 | 82 | 0.00078 | RGREILLGPADKMV |
| D3W7L4 | Polyprotein - protease NS2-3 (1a) | 1971,0724 | 440 - 456 | 82 | 0.00078 | REILLGPADKMVSKGWR** |
| D3W7L4 | Polyprotein - protease NS2-3 (1a) | 843,4524 | 443 - 450 | 82 | 0.00078 | LLGPADKM |
| D3W7L4 | Polyprotein - protease NS2-3 (1a) | 1044,5638 | 444 - 453 | 82 | 0.00078 | LGPADKMVSK |
| D3W7L4 | Polyprotein - protease NS2-3 (1a) | 1121,5652 | 448 - 456 | 82 | 0.00078 | DKMVSKGWR** |
| D3W7L4 | Polyprotein -protease NS2-3/NS3 (1a) | 2428,3525 | 455 - 475 | 82 | 0.00078 | WRLLAPITAYARQTRGLLGCI |
| D3W7L4 | Polyprotein - NS3 protein (1a) | 1714,9512 | 467 - 482 | 82 | 0.00078 | QTRGLLGCIIASLTGR |
| D3W7L4 | Polyprotein - NS3 protein (1a) | 831,4450 | 476 - 483 | 82 | 0.00078 | IASLTGRD |
| F4YQP9 | NS2 protein | 1213,5108 | 4 - 15 | 69 | 0.015 | EMAASCGGAVFV** |
| F4YQP9 | NS2 protein | 2958,4727 | 4 - 31 | 69 | 0.015 | EMAASCGGAVFVGLALLTLSPLYKMCLA** |
| F4YQP9 | NS2 protein | 723,2680 | 5 - 12 | 69 | 0.015 | MAASCGGA |
| F4YQP9 | NS2 protein | 1505,7912 | 6 - 21 | 69 | 0.015 | AASCGGAVFVGLALLT |
| F4YQP9 | NS2 protein | 1594,8463 | 16 - 29 | 69 | 0.015 | GLALLTLSPLYKMC** |
| F4YQP9 | NS2 protein | 1880,0052 | 21 - 35 | 69 | 0.015 | TLSPLYKMCLARLIW** |
| F4YQP9 | NS2 protein | 729,3190 | 26 - 30 | 69 | 0.015 | YKMCL** |
| F4YQP9 | NS2 protein | 784,3611 | 26 - 31 | 69 | 0.015 | YKMCLA |
| F4YQP9 | NS2 protein | 891,4670 | 28 - 34 | 69 | 0.015 | MCLARLI** |
| F4YQP9 | NS2 protein | 1061,5514 | 28 - 35 | 69 | 0.015 | MCLARLIW |
| F4YQP9 | NS2 protein | 843,4755 | 31 - 36 | 69 | 0.015 | ARLIWW |
| F4YQP9 | NS2 protein | 1774,9155 | 36 - 49 | 69 | 0.015 | WLQYFITRAEAHLQ |
| F4YQP9 | NS2 protein | 2820,5300 | 38 - 61 | 69 | 0.015 | QYFITRAEAHLQVWIPPLNVRGGR |
| F4YQP9 | NS2 protein | 1108,5778 | 43 - 51 | 69 | 0.015 | RAEAHLQVW |
| F4YQP9 | NS2 protein | 794,4439 | 47 - 52 | 69 | 0.015 | HLQVWI |
| F4YQP9 | NS2 protein | 851,4905 | 49 - 55 | 69 | 0.015 | QVWIPPL |
| F4YQP9 | NS2 protein | 2231,2031 | 53 - 72 | 69 | 0.015 | PPLNVRGGRDAIILLMCAIH** |
| F4YQP9 | NS2 protein | 770,4511 | 55 - 61 | 69 | 0.015 | LNVRGGR |
| F4YQP9 | NS2 protein | 919,4871 | 63 - 70 | 69 | 0.015 | AIILLMCA** |
| F4YQP9 | NS2 protein | 1153,6351 | 63 - 72 | 69 | 0.015 | AIILLMCAIH |
| F4YQP9 | NS2 protein | 777,4129 | 64 - 69 | 69 | 0.015 | IILLMC** |
| F4YQP9 | NS2 protein | 832,4550 | 65 - 71 | 69 | 0.015 | ILLMCAI |
| F4YQP9 | NS2 protein | 953,4827 | 66 - 73 | 69 | 0.015 | LLMCAIHP |
| F4YQP9 | NS2 protein | 1197,6791 | 85 - 96 | 69 | 0.015 | AILGPLMVLQAG** |
| F4YQP9 | NS2 protein | 869,5045 | 87 - 94 | 69 | 0.015 | LGPLMVLQ |
| F4YQP9 | NS2 protein | 3024,7106 | 94 - 119 | 69 | 0.015 | QAGITRVPYFVRAHGLIRACLLMRKV |
| F4YQP9 | NS2 protein | 1277,7244 | 96 - 106 | 69 | 0.015 | GITRVPYFVRA |
| F4YQP9 | NS2 protein | 1044,5505 | 100 - 108 | 69 | 0.015 | VPYFVRAHG |
| F4YQP9 | NS2 protein | 1157,6346 | 100 - 109 | 69 | 0.015 | VPYFVRAHGL |
| F4YQP9 | NS2 protein | 3173,6460 | 105 - 131 | 69 | 0.015 | RAHGLIRACLLMRKVAGGHYVQMAFMR** |
| F4YQP9 | NS2 protein | 1031,5732 | 110 - 117 | 69 | 0.015 | IRACLLMR |
| F4YQP9 | NS2 protein | 2499,2371 | 110 - 130 | 69 | 0.015 | IRACLLMRKVAGGHYVQMAFM** |
| F4YQP9 | NS2 protein | 1273,7111 | 111 - 121 | 69 | 0.015 | RACLLMRKVAG |
| F4YQP9 | NS2 protein | 1467,7915 | 111 - 123 | 69 | 0.015 | RACLLMRKVAGGH |
| F4YQP9 | NS2 protein | 2214,0570 | 112 - 130 | 69 | 0.015 | ACLLMRKVAGGHYVQMAFM** |
| F4YQP9 | NS2 protein | 1518,7799 | 113 - 125 | 69 | 0.015 | CLLMRKVAGGHYV** |
| F4YQP9 | NS2 protein | 1357,7289 | 115 - 126 | 69 | 0.015 | LMRKVAGGHYVQ |
| F4YQP9 | NS2 protein | 2210,1275 | 115 - 133 | 69 | 0.015 | LMRKVAGGHYVQMAFMRLA** |
| F4YQP9 | NS2 protein | 1756,8211 | 116 - 130 | 69 | 0.015 | MRKVAGGHYVQMAFM** |
| F4YQP9 | NS2 protein | 2281,1646 | 116 - 135 | 69 | 0.015 | MRKVAGGHYVQMAFMRLAAL** |
| F4YQP9 | NS2 protein | 829,4446 | 118 - 125 | 69 | 0.015 | KVAGGHYV |
| F4YQP9 | NS2 protein | 829,4083 | 119 - 126 | 69 | 0.015 | VAGGHYVQ |
| F4YQP9 | NS2 protein | 1310,6264 | 123 - 132 | 69 | 0.015 | HYVQMAFMRL** |
| F4YQP9 | NS2 protein | 1244,6046 | 124 - 133 | 69 | 0.015 | YVQMAFMRLA** |
| F4YQP9 | NS2 protein | 1540,7742 | 125 - 138 | 69 | 0.015 | VQMAFMRLAALTGT** |
| F4YQP9 | NS2 protein | 2050,0557 | 130 - 147 | 69 | 0.015 | MRLAALTGTYVYDHLTPL** |
| F4YQP9 | NS2 protein | 1226,6659 | 131 - 141 | 69 | 0.015 | RLAALTGTYVY |
| F4YQP9 | NS2 protein | 1423,6983 | 133 - 145 | 69 | 0.015 | AALTGTYVYDHLT |
| F4YQP9 | NS2 protein | 1789,9363 | 133 - 148 | 69 | 0.015 | AALTGTYVYDHLTPLR |
| F4YQP9 | NS2 protein | 1762,8890 | 135 - 149 | 69 | 0.015 | LTGTYVYDHLTPLRD |
| F4YQP9 | NS2 protein | 899,4865 | 144 - 150 | 69 | 0.015 | LTPLRDW |
| F4YQP9 | NS2 protein | 1607,8168 | 148 - 161 | 69 | 0.015 | RDWAHEGLRDLAVA |
| F4YQP9 | NS2 protein | 2214,0627 | 152 - 171 | 69 | 0.015 | HEGLRDLAVAVEPVTFSDME |
| F4YQP9 | NS2 protein | 2863,4314 | 153 - 177 | 69 | 0.015 | EGLRDLAVAVEPVEFSDMETKIITW** |
| F4YQP9 | NS2 protein | 1848,8928 | 156 - 172 | 69 | 0.015 | RDLAVAVEPVAFSDMET |
| F4YQP9 | NS2 protein | 1040,5390 | 157 - 166 | 69 | 0.015 | DLAVAVEPVE |
| F4YQP9 | NS2 protein | 1976,9765 | 157 - 174 | 69 | 0.015 | DLAVAVEPVNFSDMETKI |
| F4YQP9 | NS2 protein | 933,5284 | 158 - 166 | 69 | 0.015 | LAVAVEPVH |
| F4YQP9 | NS2 protein | 1129,5656 | 159 - 169 | 69 | 0.015 | AVAVEPVPFSD |
| F4YQP9 | NS2 protein | 1565,7396 | 159 - 172 | 69 | 0.015 | AVAVEPVRFSDMET** |
| F4YQP9 | NS2 protein | 1469,6749 | 160 - 172 | 69 | 0.015 | VAVEPVFFSDMET |
| F4YQP9 | NS2 protein | 1232,4907 | 163 - 172 | 69 | 0.015 | EPVYFSDMET** |
| F4YQP9 | NS2 protein | 807,3837 | 164 - 170 | 69 | 0.015 | PVIFSDM |
| F4YQP9 | NS2 protein | 1296,5908 | 164 - 174 | 69 | 0.015 | PVDFSDMETKI** |
| F4YQP9 | NS2 protein | 1098,5267 | 167 - 175 | 69 | 0.015 | FSDMETKII** |
| F4YQP9 | NS2 protein | 889,4909 | 171 - 177 | 69 | 0.015 | ETKIITW |
| F4YQP9 | NS2 protein | 1832,8979 | 174 - 191 | 69 | 0.015 | IITWGADTAACGDIISGL |
| F4YQP9 | NS2 protein | 1515,7239 | 178 - 193 | 69 | 0.015 | GADTAACGDIISGLPV |
| F4YQP9 | NS2 protein | 975,4695 | 182 - 191 | 69 | 0.015 | AACGDIISGL |
| F4YQP9 | NS2 protein | 1183,6561 | 185 - 196 | 69 | 0.015 | GDIISGLPVSAR |
| F4YQP9 | NS2 protein | 1953,0068 | 198 - 215 | 69 | 0.015 | GREILLGPADSLEGQGWR |
| F4YQP9 | NS2 protein | 1454,7405 | 201 - 214 | 69 | 0.015 | ILLGPADSLEGQGW |
| F4YQP9 | NS2 protein | 1046,4781 | 207 - 215 | 69 | 0.015 | DSLEGQGWR |
| F4YQP9 | NS2 protein | 844,4191 | 210 - 216 | 69 | 0.015 | EGQGWRL |
| A3EZJ3 | NS3 protein | 1107,5673 | 3 - 12 | 81 | 0.001 | ITAYAQQTRG |
| A3EZJ3 | NS3 protein | 1714,9512 | 9 - 24 | 81 | 0.001 | QTRGLLGCIITGLTGR |
| A3EZJ3 | NS3 protein | 2428,2856 | 9 - 30 | 81 | 0.001 | QTRGLLGCIITGLTGRDKNQVE |
| A3EZJ3 | NS3 protein | 1971,0684 | 11 - 28 | 81 | 0.001 | RGLLGCIITGLTGRDKNQ |
| A3EZJ3 | NS3 protein | 831,4450 | 18 - 25 | 81 | 0.001 | ITGLTGRD |
| A3EZJ3 | NS3 protein | 2945,5458 | 18 - 45 | 81 | 0.001 | ITGLTGRDKNQVEGEVQIVSTAAQTFLA |
| A3EZJ3 | NS3 protein | 1544,7682 | 25 - 38 | 81 | 0.001 | DKNQVEGEVQIVST |
| A3EZJ3 | NS3 protein | 2247,1383 | 25 - 45 | 81 | 0.001 | DKNQVEGEVQIVSTAAQTFLA |
| A3EZJ3 | NS3 protein | 1064,5502 | 34 - 43 | 81 | 0.001 | QIVSTAAQTF |
| A3EZJ3 | NS3 protein | 1665,8396 | 35 - 50 | 81 | 0.001 | IVSTAAQTFLATCING |
| A3EZJ3 | NS3 protein | 2311,1341 | 35 - 55 | 81 | 0.001 | IVSTAAQTFLATCINGVCWTV |
| A3EZJ3 | NS3 protein | 1108,5764 | 36 - 46 | 81 | 0.001 | VSTAAQTFLAT |
| A3EZJ3 | NS3 protein | 1820,8338 | 44 - 59 | 81 | 0.001 | LATCINGVCWTVYHGA |
| A3EZJ3 | NS3 protein | 832,4403 | 58 - 66 | 81 | 0.001 | GAGTRTIAS |
| A3EZJ3 | NS3 protein | 1047,5673 | 58 - 68 | 81 | 0.001 | GAGTRTIASSK |
| A3EZJ3 | NS3 protein | 1650,8763 | 61 - 75 | 81 | 0.001 | TRTIASSKGPVIQMY |
| A3EZJ3 | NS3 protein | 1565,8235 | 62 - 75 | 81 | 0.001 | RTIASSKGPVIQMY** |
| A3EZJ3 | NS3 protein | 2120,0651 | 77 - 96 | 81 | 0.001 | NVDQDLVGWPAPQGARSLTP |
| A3EZJ3 | NS3 protein | 2141,9987 | 81 - 100 | 81 | 0.001 | DLVGWPAPQGARSLTPCTCG |
| A3EZJ3 | NS3 protein | 1540,7457 | 85 - 98 | 81 | 0.001 | WPAPQGARSLTPCT |
| A3EZJ3 | NS3 protein | 2478,1744 | 89 - 110 | 81 | 0.001 | QGARSLTPCTCGSSDLYLVTRH |
| A3EZJ3 | NS3 protein | 2945,6536 | 105 - 130 | 81 | 0.001 | YLVTRHADVIPVRRRGDGRGSLLSPR |
| A3EZJ3 | NS3 protein | 1917,0518 | 108 - 124 | 81 | 0.001 | TRHADVIPVRRRGDGRG |
| A3EZJ3 | NS3 protein | 1774,9577 | 124 - 141 | 81 | 0.001 | GSLLSPRPISYLKGSSGG |
| A3EZJ3 | NS3 protein | 1800,9556 | 130 - 146 | 81 | 0.001 | RPISYLKGSSGGPLLCP |
| A3EZJ3 | NS3 protein | 2137,1102 | 130 - 150 | 81 | 0.001 | RPISYLKGSSGGPLLCPAGHA |
| A3EZJ3 | NS3 protein | 864,4341 | 134 - 142 | 81 | 0.001 | YLKGSSGGP |
| A3EZJ3 | NS3 protein | 1475,7442 | 134 - 148 | 81 | 0.001 | YLKGSSGGPLLCPAG |
| A3EZJ3 | NS3 protein | 1953,0142 | 134 - 153 | 81 | 0.001 | YLKGSSGGPLLCPAGHAVGI |
| A3EZJ3 | NS3 protein | 1548,7719 | 137 - 153 | 81 | 0.001 | GSSGGPLLCPAGHAVGI |
| A3EZJ3 | NS3 protein | 1804,9771 | 141 - 158 | 81 | 0.001 | GPLLCPAGHAVGIFRAAV |
| A3EZJ3 | NS3 protein | 2197,1361 | 145 - 165 | 81 | 0.001 | CPAGHAVGIFRAAVCTRGVAK |
| A3EZJ3 | NS3 protein | 1248,6761 | 151 - 161 | 81 | 0.001 | VGIFRAAVCTR |
| A3EZJ3 | NS3 protein | 844,4178 | 171 - 178 | 81 | 0.001 | PVEGLETT |
| A3EZJ3 | NS3 protein | 975,4583 | 171 - 179 | 81 | 0.001 | PVEGLETTM |
| A3EZJ3 | NS3 protein | 2641,2694 | 171 - 195 | 81 | 0.001 | PVEGLETTMRSPVFSDNSSPPAVPQ |
| A3EZJ3 | NS3 protein | 1121,5387 | 172 - 181 | 81 | 0.001 | VEGLETTMRS |
| A3EZJ3 | NS3 protein | 1490,6824 | 179 - 192 | 81 | 0.001 | MRSPVFSDNSSPPA |
| A3EZJ3 | NS3 protein | 764,3341 | 182 - 188 | 81 | 0.001 | PVFSDNS |
| A3EZJ3 | NS3 protein | 1098,5458 | 192 - 201 | 81 | 0.001 | AVPQSYQVAH |
| A3EZJ3 | NS3 protein | 1423,6844 | 196 - 209 | 81 | 0.001 | SYQVAHLHAPTGSG |
| A3EZJ3 | NS3 protein | 1377,7252 | 208 - 221 | 81 | 0.001 | SGKSTKVPAAYAAQ |
| A3EZJ3 | NS3 protein | 2229,2521 | 213 - 234 | 81 | 0.001 | KVPAAYAAQGYKVLVLNPSVAA |
| A3EZJ3 | NS3 protein | 2658,3476 | 226 - 251 | 81 | 0.001 | LVLNPSVAATLGFGAYMSKAHGIDPN** |
| A3EZJ3 | NS3 protein | 1244,6765 | 227 - 239 | 81 | 0.001 | VLNPSVAATLGFG |
| A3EZJ3 | NS3 protein | 3112,6240 | 228 - 257 | 81 | 0.001 | LNPSVAATLGFGAYMSKAHGIDPNIRTGVR |
| A3EZJ3 | NS3 protein | 714,3912 | 230 - 237 | 81 | 0.001 | PSVAATLG |
| A3EZJ3 | NS3 protein | 1724,8192 | 233 - 249 | 81 | 0.001 | AATLGFGAYMSKAHGID** |
| A3EZJ3 | NS3 protein | 1789,8821 | 236 - 252 | 81 | 0.001 | LGFGAYMSKAHGIDPNI |
| A3EZJ3 | NS3 protein | 1594,8250 | 242 - 256 | 81 | 0.001 | MSKAHGIDPNIRTGV |
| A3EZJ3 | NS3 protein | 1277,6840 | 244 - 255 | 81 | 0.001 | KAHGIDPNIRTG |
| A3EZJ3 | NS3 protein | 1147,6310 | 253 - 263 | 81 | 0.001 | RTGVRTITTGS |
| A3EZJ3 | NS3 protein | 2788,2538 | 260 - 286 | 81 | 0.001 | TTGSPITYSTYGKFLADGGCSGSAYDI |
| A3EZJ3 | NS3 protein | 717,3333 | 267 - 272 | 81 | 0.001 | YSTYGK |
| A3EZJ3 | NS3 protein | 1402,6398 | 291 - 303 | 81 | 0.001 | ECHSTDATSILGI |
| A3EZJ3 | NS3 protein | 1614,8465 | 299 - 315 | 81 | 0.001 | SILGIGTVLDQAETAGA |
| A3EZJ3 | NS3 protein | 1553,8777 | 315 - 330 | 81 | 0.001 | ARLTVLATATPPGSVT |
| A3EZJ3 | NS3 protein | 849,4345 | 328 - 335 | 81 | 0.001 | SVTVPHPN |
| A3EZJ3 | NS3 protein | 1091,5611 | 328 - 337 | 81 | 0.001 | SVTVPHPNIE |
| A3EZJ3 | NS3 protein | 848,4127 | 337 - 344 | 81 | 0.001 | EEVALSTT |
| A3EZJ3 | NS3 protein | 2134,1198 | 338 - 357 | 81 | 0.001 | EVALSTTGEIPFYGKAIPLE |
| A3EZJ3 | NS3 protein | 1206,6002 | 366 - 374 | 81 | 0.001 | IFCHSKKKC |
| A3EZJ3 | NS3 protein | 2117,1125 | 367 - 384 | 81 | 0.001 | FCHSKKKCDELAAKLVAL |
| A3EZJ3 | NS3 protein | 1143,5706 | 369 - 377 | 81 | 0.001 | HSKKKCDEL |
| A3EZJ3 | NS3 protein | 1197,6969 | 375 - 386 | 81 | 0.001 | DELAAKLVALGV |
| A3EZJ3 | NS3 protein | 2050,0735 | 388 - 407 | 81 | 0.001 | AVAYYRGLDVSVIPASGDVV |
| A3EZJ3 | NS3 protein | 1467,8185 | 395 - 409 | 81 | 0.001 | LDVSVIPASGDVVVV |
| A3EZJ3 | NS3 protein | 843,4338 | 398 - 406 | 81 | 0.001 | SVIPASGDV |
| A3EZJ3 | NS3 protein | 855,4702 | 399 - 407 | 81 | 0.001 | VIPASGDVV |
| A3EZJ3 | NS3 protein | 2036,0137 | 400 - 420 | 81 | 0.001 | IPASGDVVVVATDALMTGFTG** |
| A3EZJ3 | NS3 protein | 2451,0393 | 413 - 434 | 81 | 0.001 | ALMTGFTGDFDSVIDCNTCVTQ |
| A3EZJ3 | NS3 protein | 2187,9301 | 423 - 441 | 81 | 0.001 | DSVIDCNTCVTQTVDFSLD |
| A3EZJ3 | NS3 protein | 2765,2049 | 427 - 450 | 81 | 0.001 | DCNTCVTQTVDFSLDPTFTIETTT |
| A3EZJ3 | NS3 protein | 1545,7635 | 447 - 460 | 81 | 0.001 | ETTTLPQDAVSRTQ |
| A3EZJ3 | NS3 protein | 1575,8522 | 468 - 481 | 81 | 0.001 | GKPGIYRFVTPGER |
| A3EZJ3 | NS3 protein | 2112,0099 | 470 - 488 | 81 | 0.001 | PGIYRFVTPGERPSGMFDS |
| A3EZJ3 | NS3 protein | 1774,8971 | 512 - 526 | 81 | 0.001 | RLRAYMNTPGLPVCQ |
| A3EZJ3 | NS3 protein | 2609,1719 | 515 - 536 | 81 | 0.001 | AYMNTPGLPVCQDHLEFWEGVF |
| A3EZJ3 | NS3 protein | 1826,8298 | 519 - 533 | 81 | 0.001 | TPGLPVCQDHLEFWE |
| A3EZJ3 | NS3 protein | 2214,0382 | 526 - 544 | 81 | 0.001 | QDHLEFWEGVFTGLTHIDA |
| A3EZJ3 | NS3 protein | 1762,8567 | 529 - 543 | 81 | 0.001 | LEFWEGVFTGLTHID |
| A3EZJ3 | NS3 protein | 1030,5084 | 542 - 550 | 81 | 0.001 | IDAHFLSQT |
| A3EZJ3 | NS3 protein | 1879,9680 | 551 - 567 | 81 | 0.001 | KQSGENLPYLVAYQATV |
| A3EZJ3 | NS3 protein | 1698,8512 | 561 - 576 | 81 | 0.001 | VAYQATVCARAQAPPP |
| A3EZJ3 | NS3 protein | 1268,6295 | 564 - 575 | 81 | 0.001 | QATVCARAQAPP |
| A3EZJ3 | NS3 protein | 1540,7133 | 571 - 583 | 81 | 0.001 | AQAPPPSWDQMWK |
| A3EZJ3 | NS3 protein | 1731,8589 | 576 - 588 | 81 | 0.001 | PSWDQMWKCLIRL |
| A3EZJ3 | NS3 protein | 2209,1500 | 579 - 596 | 81 | 0.001 | DQMWKCLIRLKPTLHGPT** |
| A3EZJ3 | NS3 protein | 1157,6292 | 602 - 612 | 81 | 0.001 | LGAVQNEITLT |
| A3EZJ3 | NS3 protein | 1044,5451 | 603 - 612 | 81 | 0.001 | GAVQNEITLT |
| A3EZJ3 | NS3 protein | 1185,6758 | 609 - 618 | 81 | 0.001 | ITLTHPITKY |
| A3EZJ3 | NS3 protein | 1414,6658 | 614 - 625 | 81 | 0.001 | PITKYIMTCMSA |
| A3EZJ3 | NS3 protein | 1870,8879 | 614 - 629 | 81 | 0.001 | PITKYIMTCMSADLEV |
| A3EZJ3 | NS3 protein | 832,4178 | 624 - 631 | 81 | 0.001 | SADLEVVT |
| F8SI75 | NS3 protein | 1971,1153 | 1 - 19 | 112 | 8e-007 | APITAYAQQTRGLLGTIVV |
| F8SI75 | NS3 protein | 2134,1456 | 2 - 21 | 112 | 8e-007 | PITAYAQQTRGLLGTIVVSM** |
| F8SI75 | NS3 protein | 1107,5673 | 3 - 12 | 112 | 8e-007 | ITAYAQQTRG |
| F8SI75 | NS3 protein | 1277,6728 | 5 - 16 | 112 | 8e-007 | AYAQQTRGLLGT |
| F8SI75 | NS3 protein | 2137,1314 | 5 - 24 | 112 | 8e-007 | AYAQQTRGLLGTIVVSMTGR** |
| F8SI75 | NS3 protein | 2945,5241 | 9 - 35 | 112 | 8e-007 | QTRGLLGTIVVSMTGRDKTQQDGEIQV** |
| F8SI75 | NS3 protein | 2247,1642 | 11 - 31 | 112 | 8e-007 | RGLLGTIVVSMTGRDKTQQDG** |
| F8SI75 | NS3 protein | 1774,9611 | 12 - 28 | 112 | 8e-007 | GLLGTIVVSMTGRDKTQ |
| F8SI75 | NS3 protein | 1847,9411 | 14 - 30 | 112 | 8e-007 | LGTIVVSMTGRDKTQQD |
| F8SI75 | NS3 protein | 764,3851 | 18 - 24 | 112 | 8e-007 | VVSMTGR** |
| F8SI75 | NS3 protein | 1762,8520 | 18 - 33 | 112 | 8e-007 | VVSMTGRDKTQQDGEI |
| F8SI75 | NS3 protein | 1121,5499 | 19 - 28 | 112 | 8e-007 | VSMTGRDKTQ |
| F8SI75 | NS3 protein | 831,4199 | 23 - 29 | 112 | 8e-007 | GRDKTQQ |
| F8SI75 | NS3 protein | 1916,9803 | 24 - 40 | 112 | 8e-007 | RDKTQQDGEIQVLSTVT |
| F8SI75 | NS3 protein | 1879,9164 | 27 - 43 | 112 | 8e-007 | TQQDGEIQVLSTVTQSF |
| F8SI75 | NS3 protein | 1544,7682 | 28 - 41 | 112 | 8e-007 | QQDGEIQVLSTVTQ |
| F8SI75 | NS3 protein | 1108,5765 | 34 - 43 | 112 | 8e-007 | QVLSTVTQSF |
| F8SI75 | NS3 protein | 1553,8494 | 43 - 56 | 112 | 8e-007 | FLGTTISGILWTVF |
| F8SI75 | NS3 protein | 1971,0578 | 44 - 62 | 112 | 8e-007 | LGTTISGILWTVFHGAGNK |
| F8SI75 | NS3 protein | 1047,5601 | 45 - 54 | 112 | 8e-007 | GTTISGILWT |
| F8SI75 | NS3 protein | 1800,9523 | 47 - 63 | 112 | 8e-007 | TISGILWTVFHGAGNKT |
| F8SI75 | NS3 protein | 1030,5196 | 54 - 63 | 112 | 8e-007 | TVFHGAGNKT |
| F8SI75 | NS3 protein | 2117,0477 | 56 - 75 | 112 | 8e-007 | FHGAGNKTLAGPRGPVTQMY** |
| F8SI75 | NS3 protein | 1226,6442 | 63 - 74 | 112 | 8e-007 | TLAGPRGPVTQM |
| F8SI75 | NS3 protein | 1650,8036 | 63 - 78 | 112 | 8e-007 | TLAGPRGPVTQMYSSA** |
| F8SI75 | NS3 protein | 1820,8727 | 63 - 80 | 112 | 8e-007 | TLAGPRGPVTQMYSSAEG |
| F8SI75 | NS3 protein | 2120,0208 | 64 - 84 | 112 | 8e-007 | LAGPRGPVTQMYSSAEGDLVG** |
| F8SI75 | NS3 protein | 2658,2133 | 65 - 90 | 112 | 8e-007 | AGPRGPVTQMYSSAEGDLVGWSSPHG** |
| F8SI75 | NS3 protein | 2141,9688 | 68 - 87 | 112 | 8e-007 | RGPVTQMYSSAEGDLVGWSS** |
| F8SI75 | NS3 protein | 3112,3907 | 70 - 97 | 112 | 8e-007 | PVTQMYSSAEGDLVGWSSPWGTRSLEPC** |
| F8SI75 | NS3 protein | 1414,6286 | 71 - 83 | 112 | 8e-007 | VTQMYSSAEGDLV** |
| F8SI75 | NS3 protein | 1157,6193 | 82 - 92 | 112 | 8e-007 | LVGWSSPVGTR |
| F8SI75 | NS3 protein | 717,3082 | 84 - 90 | 112 | 8e-007 | GWSSPQG |
| F8SI75 | NS3 protein | 1185,6102 | 86 - 96 | 112 | 8e-007 | SSPRGTRSLEP |
| F8SI75 | NS3 protein | 855,4450 | 88 - 95 | 112 | 8e-007 | PPGTRSLE |
| F8SI75 | NS3 protein | 1244,6183 | 88 - 98 | 112 | 8e-007 | PKGTRSLEPCT |
| F8SI75 | NS3 protein | 2609,2578 | 91 - 113 | 112 | 8e-007 | TRSLEPCTCGAVDLYLVTRNADV |
| F8SI75 | NS3 protein | 1826,8291 | 97 - 112 | 112 | 8e-007 | CTCGAVDLYLVTRNAD |
| F8SI75 | NS3 protein | 848,4644 | 100 - 107 | 112 | 8e-007 | GAVDLYLV |
| F8SI75 | NS3 protein | 1091,5975 | 102 - 110 | 112 | 8e-007 | VDLYLVTRN |
| F8SI75 | NS3 protein | 1714,9366 | 103 - 117 | 112 | 8e-007 | DLYLVTRNADVIPAR |
| F8SI75 | NS3 protein | 1871,0377 | 103 - 118 | 112 | 8e-007 | DLYLVTRNADVIPARR |
| F8SI75 | NS3 protein | 2036,1100 | 107 - 124 | 112 | 8e-007 | VTRNADVIPARRQGDRRG |
| F8SI75 | NS3 protein | 1565,8499 | 111 - 124 | 112 | 8e-007 | ADVIPARRQGDRRG |
| F8SI75 | NS3 protein | 1565,8499 | 112 - 125 | 112 | 8e-007 | DVIPARRQGDRRGA |
| F8SI75 | NS3 protein | 1402,7429 | 119 - 131 | 112 | 8e-007 | QGDRRGALLSPHP |
| F8SI75 | NS3 protein | 843,4636 | 123 - 130 | 112 | 8e-007 | RGALLSPM |
| F8SI75 | NS3 protein | 1147,5907 | 132 - 143 | 112 | 8e-007 | LSMLKGSSGGPV** |
| F8SI75 | NS3 protein | 1467,7756 | 131 - 145 | 112 | 8e-007 | PLSPLKGSSGGPVLC |
| F8SI75 | NS3 protein | 1724,9243 | 131 - 147 | 112 | 8e-007 | PLSTLKGSSGGPVLCPR |
| F8SI75 | NS3 protein | 2478,3278 | 132 - 155 | 112 | 8e-007 | LSNLKGSSGGPVLCPRGHAVGIFR |
| F8SI75 | NS3 protein | 832,4113 | 134 - 142 | 112 | 8e-007 | MLKGSSGGP |
| F8SI75 | NS3 protein | 1206,5928 | 139 - 150 | 112 | 8e-007 | SGGPVLCPRGHA |
| F8SI75 | NS3 protein | 1475,7667 | 139 - 153 | 112 | 8e-007 | SGGPVLCPRGHAVGI |
| F8SI75 | NS3 protein | 2050,0717 | 141 - 159 | 112 | 8e-007 | GPVLCPRGHAVGIFRAAIC |
| F8SI75 | NS3 protein | 1064,5549 | 143 - 152 | 112 | 8e-007 | VLCPRGHAVG |
| F8SI75 | NS3 protein | 1268,6561 | 145 - 155 | 112 | 8e-007 | CPRGHAVGIFR |
| F8SI75 | NS3 protein | 1548,8558 | 154 - 167 | 112 | 8e-007 | FRAAICTRGVAKSI |
| F8SI75 | NS3 protein | 1575,8443 | 158 - 171 | 112 | 8e-007 | ICTRGVAKSIDFIP |
| F8SI75 | NS3 protein | 1789,9462 | 166 - 181 | 112 | 8e-007 | SIDFIPVESLDVITRS |
| F6L9J4 | NS3 protein | 1731,9302 | 2 - 17 | 87 | 0.00027 | TRGLLGCIITSLTGRD |
| F6L9J4 | NS3 protein | 2229,1899 | 3 - 22 | 87 | 0.00027 | RGLLGCIITSLTGRDKNQVE |
| F6L9J4 | NS3 protein | 1789,8992 | 8 - 23 | 87 | 0.00027 | CIITSLTGRDKNQVEG |
| F6L9J4 | NS3 protein | 2609,2643 | 17 - 40 | 87 | 0.00027 | DKNQVEGEVQVVSTATQSFLATCV |
| F6L9J4 | NS3 protein | 2134,0906 | 18 - 37 | 87 | 0.00027 | KNQVEGEVQVVSTATQSFLA |
| F6L9J4 | NS3 protein | 1244,6249 | 20 - 31 | 87 | 0.00027 | QVEGEVQVVSTA |
| F6L9J4 | NS3 protein | 2641,2153 | 22 - 45 | 87 | 0.00027 | EGEVQVVSTATQSFLATCVNGVCW |
| F6L9J4 | NS3 protein | 831,4338 | 24 - 31 | 87 | 0.00027 | EVQVVSTA |
| F6L9J4 | NS3 protein | 1098,5016 | 30 - 39 | 87 | 0.00027 | TATQSFLATC |
| F6L9J4 | NS3 protein | 1540,6803 | 33 - 45 | 87 | 0.00027 | QSFLATCVNGVCW |
| F6L9J4 | NS3 protein | 2311,0878 | 35 - 55 | 87 | 0.00027 | FLATCVNGVCWTVFHGAGSKT |
| F6L9J4 | NS3 protein | 1044,5352 | 48 - 58 | 87 | 0.00027 | FHGAGSKTLAG |
| F6L9J4 | NS3 protein | 2036,0248 | 53 - 71 | 87 | 0.00027 | SKTLAGPKGPITQMYTNVD** |
| F6L9J4 | NS3 protein | 1804,9029 | 55 - 71 | 87 | 0.00027 | TLAGPKGPITQMYTNVD |
| F6L9J4 | NS3 protein | 1847,9088 | 56 - 72 | 87 | 0.00027 | LAGPKGPITQMYTNVDQ** |
| F6L9J4 | NS3 protein | 1475,7442 | 57 - 70 | 87 | 0.00027 | AGPKGPITQMYTNV |
| F6L9J4 | NS3 protein | 1762,8196 | 58 - 73 | 87 | 0.00027 | GPKGPITQMYTNVDQD |
| F6L9J4 | NS3 protein | 1423,6290 | 62 - 73 | 87 | 0.00027 | PITQMYTNVDQD |
| F6L9J4 | NS3 protein | 2117,0000 | 66 - 84 | 87 | 0.00027 | MYTNVDQDLVGWQAPPGAR |
| F6L9J4 | NS3 protein | 1565,7474 | 69 - 83 | 87 | 0.00027 | NVDQDLVGWQAPPGA |
| F6L9J4 | NS3 protein | 2120,0651 | 69 - 88 | 87 | 0.00027 | NVDQDLVGWQAPPGARSLTP |
| F6L9J4 | NS3 protein | 1226,5932 | 70 - 80 | 87 | 0.00027 | VDQDLVGWQAP |
| F6L9J4 | NS3 protein | 2658,2167 | 70 - 94 | 87 | 0.00027 | VDQDLVGWQAPPGARSLTPCTCGSS |
| F6L9J4 | NS3 protein | 1030,4720 | 71 - 79 | 87 | 0.00027 | DQDLVGWQA |
| F6L9J4 | NS3 protein | 844,4079 | 72 - 78 | 87 | 0.00027 | QDLVGWQ |
| F6L9J4 | NS3 protein | 2141,9987 | 73 - 92 | 87 | 0.00027 | DLVGWQAPPGARSLTPCTCG |
| F6L9J4 | NS3 protein | 1548,8413 | 74 - 88 | 87 | 0.00027 | LVGWQAPPGARSLTP |
| F6L9J4 | NS3 protein | 2788,3636 | 83 - 107 | 87 | 0.00027 | ARSLTPCTCGSSDLYLVTRHADVIP |
| F6L9J4 | NS3 protein | 2049,9612 | 86 - 103 | 87 | 0.00027 | LTPCTCGSSDLYLVTRHA |
| F6L9J4 | NS3 protein | 2945,6172 | 101 - 126 | 87 | 0.00027 | RHADVIPVRRRGDSRGSLLSPRPVSY |
| F6L9J4 | NS3 protein | 1953,0868 | 103 - 120 | 87 | 0.00027 | ADVIPVRRRGDSRGSLLS |
| F6L9J4 | NS3 protein | 1268,6949 | 104 - 114 | 87 | 0.00027 | DVIPVRRRGDS |
| F6L9J4 | NS3 protein | 1917,0432 | 115 - 133 | 87 | 0.00027 | RGSLLSPRPVSYLKGSSGG |
| F6L9J4 | NS3 protein | 1800,9734 | 117 - 134 | 87 | 0.00027 | SLLSPRPVSYLKGSSGGP |
| F6L9J4 | NS3 protein | 2428,2784 | 117 - 140 | 87 | 0.00027 | SLLSPRPVSYLKGSSGGPSLCPLG |
| F6L9J4 | NS3 protein | 2478,2689 | 119 - 142 | 87 | 0.00027 | LSPRPVSYLKGSSGGPELCPLGHA |
| F6L9J4 | NS3 protein | 1971,0248 | 121 - 139 | 87 | 0.00027 | PRPVSYLKGSSGGPSLCPL |
| F6L9J4 | NS3 protein | 849,4596 | 123 - 130 | 87 | 0.00027 | PVSYLKGS |
| F6L9J4 | NS3 protein | 1774,8924 | 123 - 140 | 87 | 0.00027 | PVSYLKGSSGGPSLCPLG |
| F6L9J4 | NS3 protein | 3112,6427 | 126 - 155 | 87 | 0.00027 | YLKGSSGGPILCPLGHAVGIFRAAVCTRGV |
| F6L9J4 | NS3 protein | 832,4113 | 127 - 135 | 87 | 0.00027 | LKGSSGGPM |
| F6L9J4 | NS3 protein | 1121,5717 | 124 - 135 | 87 | 0.00027 | VSYLKGSSGGPA |
| F6L9J4 | NS3 protein | 1107,5673 | 125 - 135 | 87 | 0.00027 | SYLKGSSGGPR |
| F6L9J4 | NS3 protein | 717,3293 | 128 - 135 | 87 | 0.00027 | KGSSGGPE |
| F6L9J4 | NS3 protein | 1185,5812 | 128 - 140 | 87 | 0.00027 | KGSSGGPGLCPLG |
| F6L9J4 | NS3 protein | 1402,6776 | 128 - 141 | 87 | 0.00027 | KGSSGGPHLCPLGH |
| F6L9J4 | NS3 protein | 1870,9182 | 130 - 147 | 87 | 0.00027 | SSGGPMLCPLGHAVGIFR** |
| F6L9J4 | NS3 protein | 1650,8665 | 132 - 147 | 87 | 0.00027 | GGPTLCPLGHAVGIFR |
| F6L9J4 | NS3 protein | 1724,8855 | 134 - 149 | 87 | 0.00027 | PMLCPLGHAVGIFRAA** |
| F6L9J4 | NS3 protein | 1565,8613 | 135 - 148 | 87 | 0.00027 | RLCPLGHAVGIFRA |
| F6L9J4 | NS3 protein | 855,4715 | 140 - 147 | 87 | 0.00027 | GHAVGIFR |
| F6L9J4 | NS3 protein | 1248,6761 | 143 - 153 | 87 | 0.00027 | VGIFRAAVCTR |
| F6L9J4 | NS3 protein | 1575,8668 | 144 - 158 | 87 | 0.00027 | GIFRAAVCTRGVAKA |
| F6L9J4 | NS3 protein | 1880,0091 | 145 - 161 | 87 | 0.00027 | IFRAAVCTRGVAKAVDF |
| F6L9J4 | NS3 protein | 1206,6292 | 146 - 156 | 87 | 0.00027 | FRAAVCTRGVA |
| F6L9J4 | NS3 protein | 832,4337 | 147 - 153 | 87 | 0.00027 | RAAVCTR |
| F6L9J4 | NS3 protein | 1157,6557 | 153 - 163 | 87 | 0.00027 | RGVAKAVDFVP |
| K7Y470 | NS3-4 protease | 1277,6728 | 4 - 15 | 94 | 4.6e-005 | TAYAQQTRGLLG |
| K7Y470 | NS3-4 protease | 2820,4552 | 6 - 30 | 94 | 4.6e-005 | YAQQTRGLLGCIITSLTGRDKNQVE |
| K7Y470 | NS3-4 protease | 748,3715 | 19 - 25 | 94 | 4.6e-005 | TSLTGRD |
| K7Y470 | NS3-4 protease | 1344,6633 | 21 - 32 | 94 | 4.6e-005 | LTGRDKNQVEGE |
| K7Y470 | NS3-4 protease | 919,4611 | 34 - 42 | 94 | 4.6e-005 | QVVSTATQS |
| K7Y470 | NS3-4 protease | 1098,5016 | 38 - 47 | 94 | 4.6e-005 | TATQSFLATC |
| K7Y470 | NS3-4 protease | 2141,9663 | 40 - 57 | 94 | 4.6e-005 | TQSFLATCVNGVCWTVYH |
| K7Y470 | NS3-4 protease | 1540,6803 | 41 - 53 | 94 | 4.6e-005 | QSFLATCVNGVCW |
| K7Y470 | NS3-4 protease | 979,4797 | 43 - 51 | 94 | 4.6e-005 | FLATCVNGV |
| K7Y470 | NS3-4 protease | 1096,5012 | 48 - 56 | 94 | 4.6e-005 | VNGVCWTVY |
| K7Y470 | NS3-4 protease | 2503,2417 | 63 - 85 | 94 | 4.6e-005 | TLAGPKGPITQMYTNVDQDLVGW |
| K7Y470 | NS3-4 protease | 1762,8196 | 66 - 81 | 94 | 4.6e-005 | GPKGPITQMYTNVDQD |
| K7Y470 | NS3-4 protease | 1423,6290 | 70 - 81 | 94 | 4.6e-005 | PITQMYTNVDQD |
| K7Y470 | NS3-4 protease | 1226,6408 | 84 - 95 | 94 | 4.6e-005 | GWPVPSGARSLT |
| K7Y470 | NS3-4 protease | 1244,6183 | 87 - 98 | 94 | 4.6e-005 | VPSGARSLTPCT |
| K7Y470 | NS3-4 protease | 953,4706 | 100 - 108 | 94 | 4.6e-005 | GSSDLYLVT |
| K7Y470 | NS3-4 protease | 1153,6680 | 113 - 122 | 94 | 4.6e-005 | VIPVRRRGDS |
| K7Y470 | NS3-4 protease | 1044,5968 | 127 - 135 | 94 | 4.6e-005 | LSPRPISYL |
| K7Y470 | NS3-4 protease | 1953,0142 | 135 - 154 | 94 | 4.6e-005 | LKGSSGGPLLCPSGHAVGIF |
| K7Y470 | NS3-4 protease | 1183,6462 | 147 - 158 | 94 | 4.6e-005 | SGHAVGIFRAAV |
| K7Y470 | NS3-4 protease | 889,4552 | 155 - 162 | 94 | 4.6e-005 | RAAVCTRG |
| K7Y470 | NS3-4 protease | 2210,0712 | 158 - 177 | 94 | 4.6e-005 | VCTRGVAKAVDFVPVESMET** |
| K7Y470 | NS3-4 protease | 1870,8805 | 165 - 180 | 94 | 4.6e-005 | KAVDFVPVESMETTMR** |
| K7Y470 | NS3-4 protease | 1756,7648 | 168 - 182 | 94 | 4.6e-005 | DFVPVESMETTMRSP** |
| K7Y470 | NS3-4 protease | 1789,8523 | 181 - 197 | 94 | 4.6e-005 | SPVFTDNSSPPAVPQTF |
| K7Y470 | NS3-4 protease | 1832,8945 | 183 - 199 | 94 | 4.6e-005 | VFTDNSSPPAVPQTFQV |
| K7Y470 | NS3-4 protease | 1774,9115 | 194 - 210 | 94 | 4.6e-005 | PQTFQVAHLHAPTGSGK |
| K7Y470 | NS3-4 protease | 829,4083 | 195 - 201 | 94 | 4.6e-005 | QTFQVAH |
| K7Y470 | NS3-4 protease | 2863,5708 | 201 - 228 | 94 | 4.6e-005 | HLHAPTGSGKSTKVPAAYAAQGYKVLVL |
| K7Y470 | NS3-4 protease | 3024,6396 | 202 - 231 | 94 | 4.6e-005 | LHAPTGSGKSTKVPAAYAAQGYKVLVLNPS |
| K7Y470 | NS3-4 protease | 1505,8242 | 217 - 230 | 94 | 4.6e-005 | AYAAQGYKVLVLNP |
| K7Y470 | NS3-4 protease | 777,4385 | 220 - 226 | 94 | 4.6e-005 | AQGYKVL |
| K7Y470 | NS3-4 protease | 1597,7810 | 228 - 243 | 94 | 4.6e-005 | LNPSVAATLGFGAYMS |
| K7Y470 | NS3-4 protease | 3173,6040 | 228 - 258 | 94 | 4.6e-005 | LNPSVAATLGFGAYMSKAHGVDPNIRTGGRT** |
| K7Y470 | NS3-4 protease | 2958,4923 | 230 - 257 | 94 | 4.6e-005 | PSVAATLGFGAYMSKAHGVDPNIRTGWR |
| K7Y470 | NS3-4 protease | 731,2949 | 237 - 243 | 94 | 4.6e-005 | GFGAYMS |
| K7Y470 | NS3-4 protease | 1976,9309 | 241 - 257 | 94 | 4.6e-005 | YMSKAHGVDPNIRTGCR** |
| K7Y470 | NS3-4 protease | 2214,1175 | 242 - 262 | 94 | 4.6e-005 | MSKAHGVDPNIRTGSRTITTG** |
| K7Y470 | NS3-4 protease | 1040,5727 | 249 - 257 | 94 | 4.6e-005 | DPNIRTGIR |
| K7Y470 | NS3-4 protease | 1129,5840 | 249 - 258 | 94 | 4.6e-005 | DPNIRTGTRT |
| K7Y470 | NS3-4 protease | 843,4675 | 251 - 257 | 94 | 4.6e-005 | NIRTGQR |
| K7Y470 | NS3-4 protease | 933,4880 | 254 - 263 | 94 | 4.6e-005 | TGGRTITTGA |
| K7Y470 | NS3-4 protease | 1157,6404 | 254 - 265 | 94 | 4.6e-005 | TGARTITTGAPI |
| K7Y470 | NS3-4 protease | 1467,7569 | 256 - 269 | 94 | 4.6e-005 | SRTITTGAPITYST |
| K7Y470 | NS3-4 protease | 1515,6875 | 290 - 303 | 94 | 4.6e-005 | DECHSVDATTILGV |
| K7Y470 | NS3-4 protease | 729,3293 | 293 - 299 | 94 | 4.6e-005 | HSVDATT |
| K7Y470 | NS3-4 protease | 1273,6766 | 296 - 308 | 94 | 4.6e-005 | DATTILGVGTVLD |
| K7Y470 | NS3-4 protease | 1357,7453 | 297 - 310 | 94 | 4.6e-005 | ATTILGVGTVLDQA |
| K7Y470 | NS3-4 protease | 1031,5135 | 303 - 312 | 94 | 4.6e-005 | VGTVLDQAET |
| K7Y470 | NS3-4 protease | 1131,5408 | 304 - 315 | 94 | 4.6e-005 | GTVLDQAETAGA |
| K7Y470 | NS3-4 protease | 912,4917 | 319 - 328 | 94 | 4.6e-005 | VLATATPPGS |
| K7Y470 | NS3-4 protease | 770,4538 | 352 - 358 | 94 | 4.6e-005 | KAIPIET |
| K7Y470 | NS3-4 protease | 869,5222 | 354 - 361 | 94 | 4.6e-005 | IPIETIKG |
| K7Y470 | NS3-4 protease | 844,4378 | 363 - 368 | 94 | 4.6e-005 | RHLIFC |
| K7Y470 | NS3-4 protease | 1061,5539 | 371 - 379 | 94 | 4.6e-005 | KKKCDELAA |
| K7Y470 | NS3-4 protease | 1594,8831 | 379 - 393 | 94 | 4.6e-005 | AKLSGLGLNAVAYYR |
| K7Y470 | NS3-4 protease | 2050,0293 | 400 - 420 | 94 | 4.6e-005 | IPTSGDVVVVATDALMTGFTG |
| K7Y470 | NS3-4 protease | 1879,9238 | 401 - 419 | 94 | 4.6e-005 | PTSGDVVVVATDALMTGFT |
| K7Y470 | NS3-4 protease | 2231,0305 | 404 - 425 | 94 | 4.6e-005 | GDVVVVATDALMTGFTGDFDSV** |
| K7Y470 | NS3-4 protease | 877,4215 | 409 - 417 | 94 | 4.6e-005 | VATDALMTG |
| K7Y470 | NS3-4 protease | 1498,6610 | 429 - 441 | 94 | 4.6e-005 | NTCVTQTVDFSLD |
| K7Y470 | NS3-4 protease | 708,3113 | 430 - 435 | 94 | 4.6e-005 | TCVTQT |
| K7Y470 | NS3-4 protease | 2214,0944 | 432 - 451 | 94 | 4.6e-005 | VTQTVDFSLDPTFTIETTTV |
| K7Y470 | NS3-4 protease | 1471,7083 | 438 - 450 | 94 | 4.6e-005 | FSLDPTFTIETTT |
| K7Y470 | NS3-4 protease | 807,4014 | 442 - 448 | 94 | 4.6e-005 | PTFTIET |
| K7Y470 | NS3-4 protease | 908,4491 | 442 - 449 | 94 | 4.6e-005 | PTFTIETT |
| K7Y470 | NS3-4 protease | 1108,5652 | 443 - 452 | 94 | 4.6e-005 | TFTIETTTVP |
| K7Y470 | NS3-4 protease | 1454,7702 | 451 - 463 | 94 | 4.6e-005 | VPQDAVSRSQRRG |
| K7Y470 | NS3-4 protease | 829,4631 | 460 - 466 | 94 | 4.6e-005 | QRRGRTG |
| K7Y470 | NS3-4 protease | 2171,0582 | 469 - 487 | 94 | 4.6e-005 | RSGIYRFVTPGERPSGMFD |
| K7Y470 | NS3-4 protease | 1870,9036 | 472 - 487 | 94 | 4.6e-005 | IYRFVTPGERPSGMFD |
| K7Y470 | NS3-4 protease | 1230,4897 | 483 - 493 | 94 | 4.6e-005 | SGMFDSSVLCE |
| K7Y470 | NS3-4 protease | 1956,6998 | 484 - 500 | 94 | 4.6e-005 | GMFDSSVLCECYDAGCA** |
| K7Y470 | NS3-4 protease | 1232,5285 | 524 - 532 | 94 | 4.6e-005 | VCQDHLEFW |
| K7Y470 | NS3-4 protease | 891,4273 | 551 - 558 | 94 | 4.6e-005 | KQAGMNFP |
| K7Y470 | NS3-4 protease | 2499,2328 | 551 - 573 | 94 | 4.6e-005 | KQAGANFPYLTAYQATVCARAQA |
| K7Y470 | NS3-4 protease | 1030,5084 | 542 - 550 | 94 | 4.6e-005 | IDAHFLSQT |
| K7Y470 | NS3-4 protease | 1565,7838 | 549 - 562 | 94 | 4.6e-005 | QTKQAGQNFPYLTA |
| K7Y470 | NS3-4 protease | 1469,6721 | 569 - 581 | 94 | 4.6e-005 | ARAQAPPPSWDQM** |
| K7Y470 | NS3-4 protease | 1540,7133 | 571 - 583 | 94 | 4.6e-005 | AQAPPPSWDQMWK |
| K7Y470 | NS3-4 protease | 1213,5226 | 573 - 582 | 94 | 4.6e-005 | APPPSWDQMW |
| K7Y470 | NS3-4 protease | 794,4650 | 611 - 617 | 94 | 4.6e-005 | LTHPVTK |
| K7Y470 | NS3-4 protease | 784,3611 | 617 - 622 | 94 | 4.6e-005 | KYIMAC |
| K7Y470 | NS3-4 protease | 832,4178 | 625 - 632 | 94 | 4.6e-005 | ADLEVVTS |
| K7Y470 | NS3-4 protease | 1197,7122 | 633 - 644 | 94 | 4.6e-005 | TWVLVGGVLAAL |
| K7Y470 | NS3-4 protease | 841,5062 | 634 - 641 | 94 | 4.6e-005 | WVLVGGVL |
| K7Y470 | NS3-4 protease | 1848,9655 | 638 - 656 | 94 | 4.6e-005 | GGVLAALAAYCLTDGSVVI |
| K7Y470 | NS3-4 protease | 1349,7013 | 640 - 652 | 94 | 4.6e-005 | VLAALAAYCLTQG |
| K7Y470 | NS3-4 protease | 1296,6384 | 642 - 654 | 94 | 4.6e-005 | AALAAYCLTTGSV |
| K7Y470 | NS3-4 protease | 1011,4695 | 644 - 653 | 94 | 4.6e-005 | LAAYCLTGGS |
| K7Y470 | NS3-4 protease | 1518,8116 | 644 - 658 | 94 | 4.6e-005 | LAAYCLTPGSVVIVG |
| K7Y470 | NS3-4 protease | 933,5205 | 649 - 657 | 94 | 4.6e-005 | LTMGSVVIV** |
| K7Y470 | NS3-4 protease | 899,5189 | 651 - 659 | 94 | 4.6e-005 | NGSVVIVGR |
| K7Y470 | NS3-4 protease | 714,4388 | 658 - 664 | 94 | 4.6e-005 | GRIILSG |
| K7Y470 | NS3-4 protease | 2281,1353 | 660 - 679 | 94 | 4.6e-005 | IILSGKPAXIPDREVLYXEF |
| K7Y470 | NS3-4 protease | 1310,6830 | 662 - 673 | 94 | 4.6e-005 | LSGKPAEIPDRE |
| K7Y470 | NS3-4 protease | 764,4181 | 666 - 672 | 94 | 4.6e-005 | PAPIPDR |
| K7Y470 | NS3-4 protease | 1046,5509 | 670 - 677 | 94 | 4.6e-005 | PDREVLYR |
| K7Y470 | NS3-4 protease | 975,4120 | 684 - 691 | 94 | 4.6e-005 | ECASHLPY |
| Q68586 | NS4B | 1698,8440 | 3 - 17 | 82 | 0.00077 | MWNFVSGIQYLAGLT |
| Q68586 | NS4B | 2197,0878 | 3 - 22 | 82 | 0.00077 | MWNFVSGIQYLAGLTTLPGN** |
| Q68586 | NS4B | 2050,0524 | 4 - 22 | 82 | 0.00077 | WNFVSGIQYLAGLTTLPGN |
| Q68586 | NS4B | 849,4596 | 7 - 14 | 82 | 0.00077 | VSGIQYLA |
| Q68586 | NS4B | 2134,1133 | 11 - 31 | 82 | 0.00077 | QYLAGLTTLPGNPAVASLMAF |
| Q68586 | NS4B | 841,4909 | 13 - 21 | 82 | 0.00077 | LAGLTTLPG |
| Q68586 | NS4B | 714,3912 | 16 - 22 | 82 | 0.00077 | LTTLPGN |
| Q68586 | NS4B | 1789,9284 | 16 - 33 | 82 | 0.00077 | LTTLPGNPAVASLMAFTA** |
| Q68586 | NS4B | 1731,8866 | 17 - 34 | 82 | 0.00077 | TTLPGNPAVASLMAFTAA |
| Q68586 | NS4B | 1185,6064 | 18 - 29 | 82 | 0.00077 | TLPGNPAVASLM** |
| Q68586 | NS4B | 1575,7967 | 18 - 33 | 82 | 0.00077 | TLPGNPAVASLMAFTA** |
| Q68586 | NS4B | 1545,7861 | 19 - 34 | 82 | 0.00077 | LPGNPAVASLMAFTAA** |
| Q68586 | NS4B | 1800,9080 | 20 - 38 | 82 | 0.00077 | PGNPAVASLMAFTAAVTSP |
| Q68586 | NS4B | 1565,7759 | 22 - 37 | 82 | 0.00077 | NPAVASLMAFTAAVTS** |
| Q68586 | NS4B | 1548,7858 | 23 - 38 | 82 | 0.00077 | PAVASLMAFTAAVTSP** |
| Q68586 | NS4B | 1665,8648 | 24 - 40 | 82 | 0.00077 | AVASLMAFTAAVTSPLT** |
| Q68586 | NS4B | 1594,8277 | 25 - 40 | 82 | 0.00077 | VASLMAFTAAVTSPLT** |
| Q68586 | NS4B | 1107,5634 | 28 - 38 | 82 | 0.00077 | LMAFTAAVTSP |
| Q68586 | NS4B | 831,4338 | 35 - 42 | 82 | 0.00077 | VTSPLTTN |
| Q68586 | NS4B | 2142,0456 | 36 - 54 | 82 | 0.00077 | TSPLTTNQTMFFNILGGWV** |
| Q68586 | NS4B | 2112,0350 | 37 - 55 | 82 | 0.00077 | SPLTTNQTMFFNILGGWVA** |
| Q68586 | NS4B | 2247,1147 | 38 - 57 | 82 | 0.00077 | PLTTNQTMFFNILGGWVATH |
| Q68586 | NS4B | 2658,3265 | 38 - 62 | 82 | 0.00077 | PLTTNQTMFFNILGGWVATHLAGPG** |
| Q68586 | NS4B | 2945,4535 | 38 - 64 | 82 | 0.00077 | PLTTNQTMFFNILGGWVATHLAGPWAS** |
| Q68586 | NS4B | 2478,2002 | 43 - 66 | 82 | 0.00077 | QTMFFNILGGWVATHLAGPSASSA** |
| Q68586 | NS4B | 1544,8140 | 46 - 59 | 82 | 0.00077 | FFNILGGWVATHLA |
| Q68586 | NS4B | 2428,2540 | 47 - 71 | 82 | 0.00077 | FNILGGWVATHLAGPAASSAFVVSG |
| Q68586 | NS4B | 1724,9250 | 53 - 69 | 82 | 0.00077 | WVATHLAGPIASSAFVV |
| Q68586 | NS4B | 1540,7886 | 54 - 69 | 82 | 0.00077 | VATHLAGPDASSAFVV |
| Q68586 | NS4B | 864,4454 | 55 - 63 | 82 | 0.00077 | ATHLAGPQA |
| Q68586 | NS4B | 1248,6350 | 58 - 71 | 82 | 0.00077 | LAGPSASSAFVVSG |
| Q68586 | NS4B | 1402,7456 | 58 - 72 | 82 | 0.00077 | LAGPQASSAFVVSGL |
| Q68586 | NS4B | 1871,0153 | 61 - 82 | 82 | 0.00077 | PGASSAFVVSGLAGAAIGGIGL |
| Q68586 | NS4B | 1064,5502 | 62 - 72 | 82 | 0.00077 | QASSAFVVSGL |
| Q68586 | NS4B | 1121,5717 | 62 - 74 | 82 | 0.00077 | GASSAFVVSGLAG |
| Q68586 | NS4B | 1083,6288 | 68 - 80 | 82 | 0.00077 | VVSGLAGAAIGGI |
| Q68586 | NS4B | 855,5178 | 75 - 84 | 82 | 0.00077 | AAIGGIGLGK |
| Q68586 | NS4B | 1047,5601 | 87 - 97 | 82 | 0.00077 | LDILAGYGAGV |
| Q68586 | NS4B | 1147,6237 | 89 - 101 | 82 | 0.00077 | ILAGYGAGVSGAL |
| Q68586 | NS4B | 2209,1817 | 89 - 111 | 82 | 0.00077 | ILAGYGAGVSGALVAFKIMGGEL** |
| Q68586 | NS4B | 3112,5461 | 89 - 119 | 82 | 0.00077 | ILAGYGAGVSGALVAFKIMGGELPTTEDMVN** |
| Q68586 | NS4B | 2641,2768 | 91 - 117 | 82 | 0.00077 | AGYGAGVSGALVAFKIMGGELPTTEDM |
| Q68586 | NS4B | 2120,0824 | 94 - 115 | 82 | 0.00077 | GAGVSGALVAFKIMGGELPTTE** |
| Q68586 | NS4B | 1377,7326 | 97 - 110 | 82 | 0.00077 | VSGALVAFKIMGGE |
| Q68586 | NS4B | 1108,5951 | 98 - 108 | 82 | 0.00077 | SGALVAFKIMG** |
| Q68586 | NS4B | 2311,1076 | 98 - 119 | 82 | 0.00077 | SGALVAFKIMGGELPTTEDMVN** |
| Q68586 | NS4B | 1847,9339 | 99 - 116 | 82 | 0.00077 | GALVAFKIMGGELPTTED |
| Q68586 | NS4B | 1277,6690 | 102 - 113 | 82 | 0.00077 | VAFKIMGGELPT** |
| Q68586 | NS4B | 1091,5685 | 104 - 113 | 82 | 0.00077 | FKIMGGELPT |
| Q68586 | NS4B | 843,4524 | 105 - 112 | 82 | 0.00077 | KIMGGELP |
| Q68586 | NS4B | 1762,8481 | 105 - 120 | 82 | 0.00077 | KIMGGELPTTEDMVNL** |
| Q68586 | NS4B | 1650,7481 | 107 - 121 | 82 | 0.00077 | MGGELPTTEDMVNLL** |
| Q68586 | NS4B | 1614,7811 | 109 - 123 | 82 | 0.00077 | GELPTTEDMVNLLPA** |
| Q68586 | NS4B | 2137,0977 | 109 - 129 | 82 | 0.00077 | GELPTTEDMVNLLPAILSPGA |
| Q68586 | NS4B | 1244,6322 | 111 - 121 | 82 | 0.00077 | LPTTEDMVNLL |
| Q68586 | NS4B | 1879,9965 | 111 - 128 | 82 | 0.00077 | LPTTEDMVNLLPAILSPG |
| Q68586 | NS4B | 1953,0493 | 113 - 131 | 82 | 0.00077 | TTEDMVNLLPAILSPGALV |
| Q68586 | NS4B | 1030,5005 | 114 - 122 | 82 | 0.00077 | TEDMVNLLP |
| Q68586 | NS4B | 1226,6580 | 115 - 125 | 82 | 0.00077 | EDMVNLLPAIL |
| Q68586 | NS4B | 1467,7643 | 115 - 128 | 82 | 0.00077 | EDMVNLLPAILSPG |
| Q68586 | NS4B | 2428,4603 | 118 - 141 | 82 | 0.00077 | VNLLPAILSPGALVVGVICAAILR |
| Q68586 | NS4B | 1197,7155 | 129 - 140 | 82 | 0.00077 | ALVVGVICAAIL |
| Q68586 | NS4B | 2229,2164 | 131 - 152 | 82 | 0.00077 | VVGVICAAILRRHVGPGEGAAQ |
| Q68586 | NS4B | 2945,5592 | 131 - 157 | 82 | 0.00077 | VVGVICAAILRRHVGPGEGAAQWMNRL** |
| Q68586 | NS4B | 1774,9624 | 134 - 150 | 82 | 0.00077 | VICAAILRRHVGPGEGA |
| Q68586 | NS4B | 1820,8965 | 141 - 156 | 82 | 0.00077 | RRHVGPGEGAAQWMNR |
| Q68586 | NS4B | 717,3082 | 147 - 153 | 82 | 0.00077 | GEGAAQW |
| Q68586 | NS4B | 1804,9155 | 150 - 165 | 82 | 0.00077 | AAQWMNRLIAFASRGN |
| Q68586 | NS4B | 975,4960 | 152 - 158 | 82 | 0.00077 | QWMNRLI** |
| Q68586 | NS4B | 1970,9897 | 153 - 169 | 82 | 0.00077 | WMNRLIAFASRGNHVSP** |
| Q68586 | NS4B | 1157,5941 | 158 - 168 | 82 | 0.00077 | IAFASRGNHVS |
| Q68586 | NS4B | 1044,5101 | 159 - 168 | 82 | 0.00077 | AFASRGNHVS |
| Q68586 | NS4B | 1423,6957 | 161 - 173 | 82 | 0.00077 | ASRGNHVSPTHYV |
| Q68586 | NS4B | 1206,5782 | 164 - 174 | 82 | 0.00077 | GNHVSPTHYVP |
| Q68586 | NS4B | 1414,6841 | 171 - 183 | 82 | 0.00077 | HYVPESDAAARVT |
| Q68586 | NS4B | 2214,2107 | 174 - 195 | 82 | 0.00077 | PESDAAARVTALLSSLTVTSLL |
| Q68586 | NS4B | 2117,1579 | 175 - 195 | 82 | 0.00077 | ESDAAARVTALLSSLTVTSLL |
| Q68586 | NS4B | 1715,0193 | 179 - 195 | 82 | 0.00077 | AARVTALLSSLTVTSLL |
| C9WV93 | Polyprotein - NS4B | 1665,8474 | 2 - 16 | 97 | 2.5e-005 | QAAPYIEQAQVIAHQ |
| C9WV93 | Polyprotein - NS4B | 1870,9577 | 4 - 19 | 97 | 2.5e-005 | APYIEQAQVIAHQFKE |
| C9WV93 | Polyprotein - NS4B | 2197,1895 | 5 - 23 | 97 | 2.5e-005 | PYIEQAQVIAHQFKEKVLG |
| C9WV93 | Polyprotein - NS4B | 849,4232 | 6 - 12 | 97 | 2.5e-005 | YIEQAQV |
| C9WV93 | Polyprotein - NS4B | 1098,5822 | 11 - 19 | 97 | 2.5e-005 | QVIAHQFKE |
| C9WV93 | Polyprotein - NS4B | 1226,6771 | 11 - 20 | 97 | 2.5e-005 | QVIAHQFKEK |
| C9WV93 | Polyprotein - NS4B | 1268,7241 | 13 - 23 | 97 | 2.5e-005 | IAHQFKEKVLG |
| C9WV93 | Polyprotein - NS4B | 1197,6870 | 15 - 24 | 97 | 2.5e-005 | HQFKEKVLGL |
| C9WV93 | Polyprotein - NS4B | 1553,8525 | 23 - 36 | 97 | 2.5e-005 | GLLQRATQQQAVIE |
| C9WV93 | Polyprotein - NS4B | 843,4563 | 25 - 31 | 97 | 2.5e-005 | LQRATQQ |
| C9WV93 | Polyprotein - NS4B | 1083,5560 | 28 - 37 | 97 | 2.5e-005 | ATQQQAVIEP |
| C9WV93 | Polyprotein - NS4B | 3112,5381 | 35 - 59 | 97 | 2.5e-005 | IEPIVASNWQKLEAFWHKHMWNFVS** |
| C9WV93 | Polyprotein - NS4B | 831,4239 | 39 - 45 | 97 | 2.5e-005 | VASNWQK |
| C9WV93 | Polyprotein - NS4B | 1614,7943 | 39 - 51 | 97 | 2.5e-005 | VASNWQKLEAFWH |
| C9WV93 | Polyprotein - NS4B | 2311,1109 | 39 - 56 | 97 | 2.5e-005 | VASNWQKLEAFWHKHMWN |
| C9WV93 | Polyprotein - NS4B | 1121,5505 | 41 - 49 | 97 | 2.5e-005 | SNWQKLEAF |
| C9WV93 | Polyprotein - NS4B | 2788,3333 | 41 - 62 | 97 | 2.5e-005 | SNWQKLEAFWHKHMWNFVSGIQ** |
| C9WV93 | Polyprotein - NS4B | 1185,6295 | 44 - 52 | 97 | 2.5e-005 | QKLEAFWHK |
| C9WV93 | Polyprotein - NS4B | 1540,7398 | 50 - 61 | 97 | 2.5e-005 | WHKHMWNFVSGI |
| C9WV93 | Polyprotein - NS4B | 2036,0190 | 53 - 70 | 97 | 2.5e-005 | HMWNFVSGIQYLAGLSTL |
| C9WV93 | Polyprotein - NS4B | 2658,3264 | 53 - 77 | 97 | 2.5e-005 | HMWNFVSGIQYLAGLSTLPGNPAVA** |
| C9WV93 | Polyprotein - NS4B | 2117,1157 | 56 - 76 | 97 | 2.5e-005 | NFVSGIQYLAGLSTLPGNPAV |
| C9WV93 | Polyprotein - NS4B | 1467,7973 | 57 - 70 | 97 | 2.5e-005 | FVSGIQYLAGLSTL |
| C9WV93 | Polyprotein - NS4B | 1108,5764 | 59 - 69 | 97 | 2.5e-005 | SGIQYLAGLST |
| C9WV93 | Polyprotein - NS4B | 2478,2828 | 59 - 83 | 97 | 2.5e-005 | SGIQYLAGLSTLPGNPAVASLMAFT |
| C9WV93 | Polyprotein - NS4B | 714,3912 | 65 - 72 | 97 | 2.5e-005 | AGLSTLPG |
| C9WV93 | Polyprotein - NS4B | 1731,8866 | 65 - 82 | 97 | 2.5e-005 | AGLSTLPGNPAVASLMAF** |
| C9WV93 | Polyprotein - NS4B | 2134,0980 | 67 - 88 | 97 | 2.5e-005 | LSTLPGNPAVASLMAFTASVTS |
| C9WV93 | Polyprotein - NS4B | 1490,7439 | 68 - 82 | 97 | 2.5e-005 | STLPGNPAVASLMAF** |
| C9WV93 | Polyprotein - NS4B | 1575,7967 | 69 - 84 | 97 | 2.5e-005 | TLPGNPAVASLMAFTA** |
| C9WV93 | Polyprotein - NS4B | 1545,7861 | 70 - 85 | 97 | 2.5e-005 | LPGNPAVASLMAFTAS |
| C9WV93 | Polyprotein - NS4B | 1248,6172 | 72 - 84 | 97 | 2.5e-005 | GNPAVASLMAFTA |
| C9WV93 | Polyprotein - NS4B | 1565,7759 | 73 - 88 | 97 | 2.5e-005 | NPAVASLMAFTASVTS |
| C9WV93 | Polyprotein - NS4B | 1548,7858 | 74 - 89 | 97 | 2.5e-005 | PAVASLMAFTASVTSP |
| C9WV93 | Polyprotein - NS4B | 1879,9601 | 74 - 92 | 97 | 2.5e-005 | PAVASLMAFTASVTSPLTT** |
| C9WV93 | Polyprotein - NS4B | 1594,8277 | 76 - 91 | 97 | 2.5e-005 | VASLMAFTASVTSPLT |
| C9WV93 | Polyprotein - NS4B | 2142,0456 | 87 - 105 | 97 | 2.5e-005 | TSPLTTNQTMFFNILGGWV** |
| C9WV93 | Polyprotein - NS4B | 1698,8287 | 88 - 102 | 97 | 2.5e-005 | SPLTTNQTMFFNILG** |
| C9WV93 | Polyprotein - NS4B | 2112,0350 | 88 - 106 | 97 | 2.5e-005 | SPLTTNQTMFFNILGGWVA** |
| C9WV93 | Polyprotein - NS4B | 2247,1147 | 89 - 108 | 97 | 2.5e-005 | PLTTNQTMFFNILGGWVATH |
| C9WV93 | Polyprotein - NS4B | 2765,3636 | 94 - 119 | 97 | 2.5e-005 | QTMFFNILGGWVATHLAGPQASSAFV** |
| C9WV93 | Polyprotein - NS4B | 1544,8140 | 97 - 110 | 97 | 2.5e-005 | FFNILGGWVATHLA |
| C9WV93 | Polyprotein - NS4B | 2428,2540 | 98 - 121 | 97 | 2.5e-005 | FNILGGWVATHLAGPQASSAFVVS |
| C9WV93 | Polyprotein - NS4B | 1064,5403 | 102 - 112 | 97 | 2.5e-005 | GGWVATHLAGP |
| C9WV93 | Polyprotein - NS4B | 1826,9315 | 104 - 121 | 97 | 2.5e-005 | WVATHLAGPQASSAFVVS |
| C9WV93 | Polyprotein - NS4B | 2214,0892 | 104 - 126 | 97 | 2.5e-005 | WVATHLAGPQASSAFVVSGMAGA |
| C9WV93 | Polyprotein - NS4B | 864,4454 | 106 - 114 | 97 | 2.5e-005 | ATHLAGPQA |
| C9WV93 | Polyprotein - NS4B | 1820,8727 | 110 - 130 | 97 | 2.5e-005 | AGPQASSAFVVSGMAGAAIGG** |
| C9WV93 | Polyprotein - NS4B | 1789,9033 | 112 - 131 | 97 | 2.5e-005 | PQASSAFVVSGMAGAAIGGI |
| C9WV93 | Polyprotein - NS4B | 1206,6067 | 117 - 130 | 97 | 2.5e-005 | AFVVSGMAGAAIGG |
| C9WV93 | Polyprotein - NS4B | 848,4062 | 120 - 129 | 97 | 2.5e-005 | VSGMAGAAIG** |
| C9WV93 | Polyprotein - NS4B | 832,4113 | 123 - 132 | 97 | 2.5e-005 | MAGAAIGGIG** |
| C9WV93 | Polyprotein - NS4B | 855,4814 | 124 - 134 | 97 | 2.5e-005 | AGAAIGGIGLG |
| C9WV93 | Polyprotein - NS4B | 1971,1153 | 131 - 151 | 97 | 2.5e-005 | IGLGRVLLDILAGYGAGVSGA |
| C9WV93 | Polyprotein - NS4B | 841,5022 | 132 - 139 | 97 | 2.5e-005 | GLGRVLLD |
| C9WV93 | Polyprotein - NS4B | 1801,0098 | 134 - 152 | 97 | 2.5e-005 | GRVLLDILAGYGAGVSGAL |
| C9WV93 | Polyprotein - NS4B | 1971,1153 | 134 - 154 | 97 | 2.5e-005 | GRVLLDILAGYGAGVSGALVA |
| C9WV93 | Polyprotein - NS4B | 1047,5601 | 138 - 148 | 97 | 2.5e-005 | LDILAGYGAGV |
| C9WV93 | Polyprotein - NS4B | 1147,6237 | 140 - 152 | 97 | 2.5e-005 | ILAGYGAGVSGAL |
| C9WV93 | Polyprotein - NS4B | 2209,1817 | 140 - 162 | 97 | 2.5e-005 | ILAGYGAGVSGALVAFKIMGGEL** |
| C9WV93 | Polyprotein - NS4B | 1724,8920 | 142 - 160 | 97 | 2.5e-005 | AGYGAGVSGALVAFKIMGG |
| C9WV93 | Polyprotein - NS4B | 2641,2768 | 142 - 168 | 97 | 2.5e-005 | AGYGAGVSGALVAFKIMGGELPTTEDM |
| C9WV93 | Polyprotein - NS4B | 2120,0824 | 145 - 166 | 97 | 2.5e-005 | GAGVSGALVAFKIMGGELPTTE** |
| C9WV93 | Polyprotein - NS4B | 1377,7326 | 148 - 161 | 97 | 2.5e-005 | VSGALVAFKIMGGE |
| C9WV93 | Polyprotein - NS4B | 2050,0293 | 148 - 167 | 97 | 2.5e-005 | VSGALVAFKIMGGELPTTED** |
| C9WV93 | Polyprotein - NS4B | 1847,9339 | 150 - 167 | 97 | 2.5e-005 | GALVAFKIMGGELPTTED |
| C9WV93 | Polyprotein - NS4B | 1277,6690 | 153 - 164 | 97 | 2.5e-005 | VAFKIMGGELPT** |
| C9WV93 | Polyprotein - NS4B | 1091,5685 | 155 - 164 | 97 | 2.5e-005 | FKIMGGELPT |
| C9WV93 | Polyprotein - NS4B | 1762,8481 | 156 - 171 | 97 | 2.5e-005 | KIMGGELPTTEDMVNL** |
| C9WV93 | Polyprotein - NS4B | 2229,1272 | 157 - 177 | 97 | 2.5e-005 | IMGGELPTTEDMVNLLPAILS** |
| C9WV93 | Polyprotein - NS4B | 1650,7481 | 158 - 172 | 97 | 2.5e-005 | MGGELPTTEDMVNLL** |
| C9WV93 | Polyprotein - NS4B | 1244,6322 | 162 - 172 | 97 | 2.5e-005 | LPTTEDMVNLL |
| C9WV93 | Polyprotein - NS4B | 1953,0493 | 164 - 182 | 97 | 2.5e-005 | TTEDMVNLLPAILSPGALV |
| C9WV93 | Polyprotein - NS4B | 1030,5005 | 165 - 173 | 97 | 2.5e-005 | TEDMVNLLP |
| C9WV93 | Polyprotein - NS4B | 2428,4603 | 169 - 192 | 97 | 2.5e-005 | VNLLPAILSPGALVVGVICAAILR |
| C9WV93 | Polyprotein - NS4B | 2945,5480 | 179 - 206 | 97 | 2.5e-005 | GALVVGVICAAILRRHVGPGEGAVQWMN** |
| C9WV93 | Polyprotein - NS4B | 1774,9624 | 185 - 201 | 97 | 2.5e-005 | VICAAILRRHVGPGEGA |
| C9WV93 | Polyprotein - NS4B | 1774,9624 | 186 - 202 | 97 | 2.5e-005 | ICAAILRRHVGPGEGAV |
| C9WV93 | Polyprotein - NS4B | 1804,9519 | 190 - 205 | 97 | 2.5e-005 | ILRRHVGPGEGAVQWM |
| C9WV93 | Polyprotein - NS4B | 2137,1003 | 193 - 211 | 97 | 2.5e-005 | RHVGPGEGAVQWMNRLIAF |
| C9WV93 | Polyprotein - NS4B | 2609,2921 | 197 - 220 | 97 | 2.5e-005 | PGEGAVQWMNRLIAFASRGNHVSP** |
| C9WV93 | Polyprotein - NS4B | 1402,7027 | 199 - 210 | 97 | 2.5e-005 | EGAVQWMNRLIA** |
| C9WV93 | Polyprotein - NS4B | 1475,7707 | 200 - 212 | 97 | 2.5e-005 | GAVQWMNRLIAFA |
| C9WV93 | Polyprotein - NS4B | 832,4014 | 202 - 207 | 97 | 2.5e-005 | VQWMNR |
| C9WV93 | Polyprotein - NS4B | 975,4960 | 203 - 209 | 97 | 2.5e-005 | QWMNRLI** |
| C9WV93 | Polyprotein - NS4B | 1157,5941 | 209 - 219 | 97 | 2.5e-005 | IAFASRGNHVS |
| C9WV93 | Polyprotein - NS4B | 1044,5101 | 210 - 219 | 97 | 2.5e-005 | AFASRGNHVS |
| C9WV93 | Polyprotein - NS4B | 1423,6957 | 212 - 224 | 97 | 2.5e-005 | ASRGNHVSPTHYV |
| C9WV93 | Polyprotein - NS4B | 1414,6841 | 222 - 234 | 97 | 2.5e-005 | HYVPESDAAARVT |
| C9WV93 | Polyprotein - NS4B | 1715,0193 | 230 - 246 | 97 | 2.5e-005 | AARVTALLSSLTVTSLL |
| C9WV93 | Polyprotein - NS4B | 851,4766 | 248 - 253 | 97 | 2.5e-005 | RLHQWI |
| A9JKN5 | Polyprotein - NS5A | 1762,8138 | 3 - 15 | 68 | 0.02 | DWLRDIWDWVCTV |
| A9JKN5 | Polyprotein - NS5A | 877,4227 | 8 - 13 | 68 | 0.02 | IWDWVC |
| A9JKN5 | Polyprotein - NS5A | 2231,0762 | 8 - 24 | 68 | 0.02 | IWDWVCTVLYDFKTWLS |
| A9JKN5 | Polyprotein - NS5A | 1244,6200 | 12 - 21 | 68 | 0.02 | VCTVLYDFKT |
| A9JKN5 | Polyprotein - NS5A | 3173,6004 | 13 - 39 | 68 | 0.02 | CTVLYDFKTWLSAKIMPTLPGLPFISC** |
| A9JKN5 | Polyprotein - NS5A | 1183,6416 | 15 - 23 | 68 | 0.02 | VLYDFKTWL |
| A9JKN5 | Polyprotein - NS5A | 3024,5857 | 15 - 40 | 68 | 0.02 | VLYDFKTWLSAKIMPTLPGLPFISCQ |
| A9JKN5 | Polyprotein - NS5A | 1129,5727 | 17 - 25 | 68 | 0.02 | YDFKTWLSA |
| A9JKN5 | Polyprotein - NS5A | 2863,5492 | 20 - 44 | 68 | 0.02 | KTWLSAKIMPTLPGLPFISCQKGYK |
| A9JKN5 | Polyprotein - NS5A | 1061,5327 | 21 - 29 | 68 | 0.02 | TWLSAKIMP** |
| A9JKN5 | Polyprotein - NS5A | 869,5167 | 25 - 32 | 68 | 0.02 | AKIMPTLP |
| A9JKN5 | Polyprotein - NS5A | 1197,6806 | 27 - 37 | 68 | 0.02 | IMPTLPGLPFI |
| A9JKN5 | Polyprotein - NS5A | 2050,0710 | 30 - 47 | 68 | 0.02 | TLPGLPFISCQKGYKGVW |
| A9JKN5 | Polyprotein - NS5A | 889,4527 | 32 - 39 | 68 | 0.02 | PGLPFISC |
| A9JKN5 | Polyprotein - NS5A | 1098,5360 | 35 - 43 | 68 | 0.02 | PFISCQKGY |
| A9JKN5 | Polyprotein - NS5A | 1226,6127 | 35 - 44 | 68 | 0.02 | PFISCQKGYK |
| A9JKN5 | Polyprotein - NS5A | 1349,7227 | 40 - 51 | 68 | 0.02 | QKGYKGVWRGDG |
| A9JKN5 | Polyprotein - NS5A | 2141,9558 | 42 - 60 | 68 | 0.02 | GYKGVWRGDGVMSTRCPCG |
| A9JKN5 | Polyprotein - NS5A | 807,4027 | 43 - 48 | 68 | 0.02 | YKGVWR |
| A9JKN5 | Polyprotein - NS5A | 979,4865 | 43 - 50 | 68 | 0.02 | YKGVWRGD |
| A9JKN5 | Polyprotein - NS5A | 1607,7661 | 44 - 57 | 68 | 0.02 | KGVWRGDGVMSTRC |
| A9JKN5 | Polyprotein - NS5A | 975,4437 | 45 - 53 | 68 | 0.02 | GVWRGDGVM |
| A9JKN5 | Polyprotein - NS5A | 1976,8503 | 49 - 67 | 68 | 0.02 | GDGVMSTRCPCGASITGHV** |
| A9JKN5 | Polyprotein - NS5A | 2171,0510 | 56 - 75 | 68 | 0.02 | RCPCGASITGHVKNGSMRLA |
| A9JKN5 | Polyprotein - NS5A | 1296,6547 | 58 - 70 | 68 | 0.02 | PCGASITGHVKNG |
| A9JKN5 | Polyprotein - NS5A | 1597,8358 | 60 - 75 | 68 | 0.02 | GASITGHVKNGSMRLA |
| A9JKN5 | Polyprotein - NS5A | 1031,5527 | 67 - 76 | 68 | 0.02 | VKNGSMRLAG |
| A9JKN5 | Polyprotein - NS5A | 891,4627 | 68 - 75 | 68 | 0.02 | KNGSMRLA** |
| A9JKN5 | Polyprotein - NS5A | 2499,1610 | 69 - 90 | 68 | 0.02 | NGSMRLAGPRTCANMWYGTFPI |
| A9JKN5 | Polyprotein - NS5A | 1044,5575 | 70 - 79 | 68 | 0.02 | GSMRLAGPRT |
| A9JKN5 | Polyprotein - NS5A | 1213,5181 | 77 - 85 | 68 | 0.02 | PRTCANMWY** |
| A9JKN5 | Polyprotein - NS5A | 1505,8103 | 107 - 119 | 68 | 0.02 | TRALWRVAANSYV |
| A9JKN5 | Polyprotein - NS5A | 899,5227 | 109 - 116 | 68 | 0.02 | ALWRVAAN |
| A9JKN5 | Polyprotein - NS5A | 829,4427 | 120 - 126 | 68 | 0.02 | EVRRVGD |
| A9JKN5 | Polyprotein - NS5A | 748,3627 | 122 - 127 | 68 | 0.02 | RRVGDF |
| A9JKN5 | Polyprotein - NS5A | 1423,6256 | 124 - 136 | 68 | 0.02 | VGDFHYITGATED |
| A9JKN5 | Polyprotein - NS5A | 1565,7371 | 138 - 150 | 68 | 0.02 | LKCPCQVPAAEFF |
| A9JKN5 | Polyprotein - NS5A | 1108,5727 | 144 - 153 | 68 | 0.02 | VPAAEFFTEV |
| A9JKN5 | Polyprotein - NS5A | 1518,7467 | 147 - 159 | 68 | 0.02 | AEFFTEVDGVRLH |
| A9JKN5 | Polyprotein - NS5A | 1310,6427 | 148 - 158 | 68 | 0.02 | EFFTEVDGVRL |
| A9JKN5 | Polyprotein - NS5A | 794,4729 | 153 - 159 | 68 | 0.02 | VDGVRLH |
| A9JKN5 | Polyprotein - NS5A | 1471,6653 | 171 - 183 | 68 | 0.02 | DEINFMVGLNSYA |
| A9JKN5 | Polyprotein - NS5A | 1515,7028 | 174 - 187 | 68 | 0.02 | NFMVGLNSYAIGSQ** |
| A9JKN5 | Polyprotein - NS5A | 1498,7490 | 175 - 188 | 68 | 0.02 | FMVGLNSYAIGSQL |
| A9JKN5 | Polyprotein - NS5A | 1832,8615 | 177 - 193 | 68 | 0.02 | VGLNSYAIGSQLPCEPE |
| A9JKN5 | Polyprotein - NS5A | 2214,0661 | 185 - 204 | 68 | 0.02 | GSQLPCEPEPDVSVLTSMLR |
| A9JKN5 | Polyprotein - NS5A | 1756,8189 | 186 - 201 | 68 | 0.02 | SQLPCEPEPDVSVLTS |
| A9JKN5 | Polyprotein - NS5A | 1870,9459 | 196 - 212 | 68 | 0.02 | VSVLTSMLRDPSHITAE** |
| A9JKN5 | Polyprotein - NS5A | 1357,6660 | 197 - 208 | 68 | 0.02 | SVLTSMLRDPSH** |
| A9JKN5 | Polyprotein - NS5A | 1454,7551 | 197 - 209 | 68 | 0.02 | SVLTSMLRDPSHI |
| A9JKN5 | Polyprotein - NS5A | 933,5027 | 198 - 205 | 68 | 0.02 | VLTSMLRD |
| A9JKN5 | Polyprotein - NS5A | 1046,5227 | 198 - 206 | 68 | 0.02 | VLTSMLRDP** |
| A9JKN5 | Polyprotein - NS5A | 2281,0355 | 189 - 208 | 68 | 0.02 | PCEPEPDVSVLTSMLRDPSH** |
| A9JKN5 | Polyprotein - NS5A | 1953,0391 | 209 - 227 | 68 | 0.02 | ITAETAARRLARGSPPSEA |
| A9JKN5 | Polyprotein - NS5A | 1467,7906 | 212 - 225 | 68 | 0.02 | ETAARRLARGSPPS |
| A9JKN5 | Polyprotein - NS5A | 1870,9609 | 213 - 231 | 68 | 0.02 | TAARRLARGSPPSEASSSA |
| A9JKN5 | Polyprotein - NS5A | 1131,5279 | 220 - 231 | 68 | 0.02 | RGSPPSEASSSA |
| A9JKN5 | Polyprotein - NS5A | 1540,7668 | 233 - 246 | 68 | 0.02 | QLSAPSLKATCQTH |
| A9JKN5 | Polyprotein - NS5A | 714,4199 | 259 - 263 | 68 | 0.02 | LLWRQ |
| A9JKN5 | Polyprotein - NS5A | 832,4527 | 269 - 275 | 68 | 0.02 | ITRVESE |
| A9JKN5 | Polyprotein - NS5A | 2958,5186 | 269 - 294 | 68 | 0.02 | ITRVESETKVVILDSFEPLRAEPDDT |
| A9JKN5 | Polyprotein - NS5A | 1879,9058 | 262 - 277 | 68 | 0.02 | RQEMGSNITRVESETK** |
| A9JKN5 | Polyprotein - NS5A | 2820,4215 | 263 - 287 | 68 | 0.02 | QEMGSNITRVESETKVVILDSFEPL |
| A9JKN5 | Polyprotein - NS5A | 1273,6779 | 298 - 308 | 68 | 0.02 | VAAECFKKPPK |
| A9JKN5 | Polyprotein - NS5A | 807,4052 | 301 - 306 | 68 | 0.02 | ECFKKP |
| A9JKN5 | Polyprotein - NS5A | 1277,6829 | 306 - 316 | 68 | 0.02 | PPKYPPALPIW |
| A9JKN5 | Polyprotein - NS5A | 851,4961 | 313 - 319 | 68 | 0.02 | LPIWARP |
| A9JKN5 | Polyprotein - NS5A | 919,4611 | 315 - 321 | 68 | 0.02 | IWARPDY |
| A9JKN5 | Polyprotein - NS5A | 1848,9522 | 322 - 337 | 68 | 0.02 | NPPLLDRWKAPDYAPP |
| A9JKN5 | Polyprotein - NS5A | 1469,7667 | 323 - 334 | 68 | 0.02 | PPLLDRWKAPDY |
| A9JKN5 | Polyprotein - NS5A | 729,3088 | 331 - 337 | 68 | 0.02 | APDYAPP |
| A9JKN5 | Polyprotein - NS5A | 1789,8458 | 331 - 347 | 68 | 0.02 | APDYAPPTVHGCALPPQ |
| A9JKN5 | Polyprotein - NS5A | 1594,7926 | 336 - 351 | 68 | 0.02 | PPTVHGCALPPQGAPP |
| A9JKN5 | Polyprotein - NS5A | 844,4341 | 361 - 368 | 68 | 0.02 | IQLDGSNV |
| A9JKN5 | Polyprotein - NS5A | 1774,9101 | 364 - 381 | 68 | 0.02 | DGSNVSAALAVLAEKSFP |
| A9JKN5 | Polyprotein - NS5A | 2503,2442 | 367 - 390 | 68 | 0.02 | NVSAALAVLAEKSFPSSKPEEENS |
| A9JKN5 | Polyprotein - NS5A | 1157,6127 | 368 - 379 | 68 | 0.02 | VSAALAVLAEKS |
| A9JKN5 | Polyprotein - NS5A | 843,5027 | 369 - 377 | 68 | 0.02 | SAALAVLAE |
| A9JKN5 | Polyprotein - NS5A | 908,4791 | 377 - 384 | 68 | 0.02 | EKSFPSSK |
| A9JKN5 | Polyprotein - NS5A | 748,3682 | 379 - 385 | 68 | 0.02 | SFPSSKP |
| A9JKN5 | Polyprotein - NS5A | 1030,5133 | 398 - 407 | 68 | 0.02 | QSSTTSKVPP |
| A9JKN5 | Polyprotein - NS5A | 912,4826 | 401 - 409 | 68 | 0.02 | TTSKVPPSP |
| A9JKN5 | Polyprotein - NS5A | 953,4779 | 404 - 413 | 68 | 0.02 | KVPPSPGGES |
| A9JKN5 | Polyprotein - NS5A | 1040,5427 | 403 - 413 | 68 | 0.02 | SKVPPSPGGES |
| A9JKN5 | Polyprotein - NS5A | 708,2798 | 410 - 417 | 68 | 0.02 | GGESGSES |
| A9JKN5 | Polyprotein - NS5A | 1230,4901 | 419 - 430 | 68 | 0.02 | SSMPPLEGEPGD** |
| A9JKN5 | Polyprotein - NS5A | 1956,8047 | 422 - 439 | 68 | 0.02 | PPLEGEPGDPDLSCDSWS |
| A9JKN5 | Polyprotein - NS5A | 705,2368 | 431 - 436 | 68 | 0.02 | PDLSCD |
| A9JKP2 | Polyprotein - NS5A | 1762,8138 | 3 - 15 | 72 | 0.008 | DWLRDIWDWVCTV |
| A9JKP2 | Polyprotein - NS5A | 877,3793 | 8 - 13 | 72 | 0.008 | IWDWVC |
| A9JKP2 | Polyprotein - NS5A | 1296,6319 | 12 - 21 | 72 | 0.008 | VCTVLCDFKR |
| A9JKP2 | Polyprotein - NS5A | 2231,1959 | 15 - 33 | 72 | 0.008 | VLCDFKRWLSAKIMPTLPG |
| A9JKP2 | Polyprotein - NS5A | 1597,9014 | 21 - 34 | 72 | 0.008 | RWLSAKIMPTLPGL** |
| A9JKP2 | Polyprotein - NS5A | 2499,3494 | 21 - 42 | 72 | 0.008 | RWLSAKIMPTLPGLPFISCQKG |
| A9JKP2 | Polyprotein - NS5A | 2863,5241 | 21 - 45 | 72 | 0.008 | RWLSAKIMPTLPGLPFISCQKGYKG.V** |
| A9JKP2 | Polyprotein - NS5A | 1061,5579 | 22 - 30 | 72 | 0.008 | WLSAKIMPT** |
| A9JKP2 | Polyprotein - NS5A | 869,5044 | 25 - 32 | 72 | 0.008 | AKIMPTLP |
| A9JKP2 | Polyprotein - NS5A | 1197,6832 | 27 - 37 | 72 | 0.008 | IMPTLPGLPFI |
| A9JKP2 | Polyprotein - NS5A | 2050,0710 | 30 - 47 | 72 | 0.008 | TLPGLPFISCQKGYKGVW |
| A9JKP2 | Polyprotein - NS5A | 889,4368 | 32 - 39 | 72 | 0.008 | PGLPFISC |
| A9JKP2 | Polyprotein - NS5A | 1098,5168 | 35 - 43 | 72 | 0.008 | PFISCQKGY |
| A9JKP2 | Polyprotein - NS5A | 1226,6118 | 35 - 44 | 72 | 0.008 | PFISCQKGYK |
| A9JKP2 | Polyprotein - NS5A | 1129,5590 | 36 - 44 | 72 | 0.008 | FISCQKGYK |
| A9JKP2 | Polyprotein - NS5A | 1349,6840 | 40 - 51 | 72 | 0.008 | QKGYKGVWRGDG |
| A9JKP2 | Polyprotein - NS5A | 2141,9558 | 42 - 60 | 72 | 0.008 | GYKGVWRGDGVMSTRCPCG |
| A9JKP2 | Polyprotein - NS5A | 807,4391 | 43 - 48 | 72 | 0.008 | YKGVWR |
| A9JKP2 | Polyprotein - NS5A | 979,4876 | 43 - 50 | 72 | 0.008 | YKGVWRGD |
| A9JKP2 | Polyprotein - NS5A | 1607,7661 | 44 - 57 | 72 | 0.008 | KGVWRGDGVMSTRC |
| A9JKP2 | Polyprotein - NS5A | 975,4597 | 45 - 53 | 72 | 0.008 | GVWRGDGVM |
| A9JKP2 | Polyprotein - NS5A | 1976,8503 | 49 - 67 | 72 | 0.008 | GDGVMSTRCPCGASITGHV** |
| A9JKP2 | Polyprotein - NS5A | 2171,0510 | 56 - 75 | 72 | 0.008 | RCPCGASITGHVKNGSMRLA |
| A9JKP2 | Polyprotein - NS5A | 1031,5546 | 67 - 76 | 72 | 0.008 | VKNGSMRLAG |
| A9JKP2 | Polyprotein - NS5A | 891,4596 | 68 - 75 | 72 | 0.008 | KNGSMRLA** |
| A9JKP2 | Polyprotein - NS5A | 1044,5498 | 70 - 79 | 72 | 0.008 | GSMRLAGPRT |
| A9JKP2 | Polyprotein - NS5A | 3173,4699 | 73 - 100 | 72 | 0.008 | RLAGPRTCANMWYGTFPINEYTTGPSTP** |
| A9JKP2 | Polyprotein - NS5A | 1213,5008 | 77 - 85 | 72 | 0.008 | PRTCANMWY |
| A9JKP2 | Polyprotein - NS5A | 1505,8103 | 107 - 119 | 72 | 0.008 | TRALWRVAANSYV |
| A9JKP2 | Polyprotein - NS5A | 899,4977 | 109 - 116 | 72 | 0.008 | ALWRVAAN |
| A9JKP2 | Polyprotein - NS5A | 829,4406 | 120 - 126 | 72 | 0.008 | EVRRVGD |
| A9JKP2 | Polyprotein - NS5A | 748,3980 | 122 - 127 | 72 | 0.008 | RRVGDF |
| A9JKP2 | Polyprotein - NS5A | 1423,6256 | 124 - 136 | 72 | 0.008 | VGDFHYITGATED |
| A9JKP2 | Polyprotein - NS5A | 1565,7371 | 138 - 150 | 72 | 0.008 | LKCPCQVPAAEFF |
| A9JKP2 | Polyprotein - NS5A | 1108,5441 | 144 - 153 | 72 | 0.008 | VPAAEFFTEV |
| A9JKP2 | Polyprotein - NS5A | 1518,7467 | 147 - 159 | 72 | 0.008 | AEFFTEVDGVRLH |
| A9JKP2 | Polyprotein - NS5A | 1310,6507 | 148 - 158 | 72 | 0.008 | EFFTEVDGVRL |
| A9JKP2 | Polyprotein - NS5A | 3024,5426 | 152 - 176 | 72 | 0.008 | EVDGVRLHRYAPPCKPLLRDEINFM |
| A9JKP2 | Polyprotein - NS5A | 794,4399 | 153 - 159 | 72 | 0.008 | VDGVRLH |
| A9JKP2 | Polyprotein - NS5A | 1471,6653 | 171 - 183 | 72 | 0.008 | DEINFMVGLNSYA |
| A9JKP2 | Polyprotein - NS5A | 1515,7028 | 174 - 187 | 72 | 0.008 | NFMVGLNSYAIGSQ** |
| A9JKP2 | Polyprotein - NS5A | 1498,7490 | 175 - 188 | 72 | 0.008 | FMVGLNSYAIGSQL |
| A9JKP2 | Polyprotein - NS5A | 1832,8615 | 177 - 193 | 72 | 0.008 | VGLNSYAIGSQLPCEPE |
| A9JKP2 | Polyprotein - NS5A | 2214,0661 | 185 - 204 | 72 | 0.008 | GSQLPCEPEPDVSVLTSMLR |
| A9JKP2 | Polyprotein - NS5A | 1756,8189 | 186 - 201 | 72 | 0.008 | SQLPCEPEPDVSVLTS |
| A9JKP2 | Polyprotein - NS5A | 2281,0355 | 189 - 208 | 72 | 0.008 | PCEPEPDVSVLTSMLRDPSH** |
| A9JKP2 | Polyprotein - NS5A | 1870,9459 | 196 - 212 | 72 | 0.008 | VSVLTSMLRDPSHITAE** |
| A9JKP2 | Polyprotein - NS5A | 1357,6660 | 197 - 208 | 72 | 0.008 | SVLTSMLRDPSH |
| A9JKP2 | Polyprotein - NS5A | 1454,7551 | 197 - 209 | 72 | 0.008 | SVLTSMLRDPSHI |
| A9JKP2 | Polyprotein - NS5A | 933,4953 | 198 - 205 | 72 | 0.008 | VLTSMLRD |
| A9JKP2 | Polyprotein - NS5A | 1046,5430 | 198 - 206 | 72 | 0.008 | VLTSMLRDP** |
| A9JKP2 | Polyprotein - NS5A | 1953,0391 | 209 - 227 | 72 | 0.008 | ITAETAARRLARGSPPSEA |
| A9JKP2 | Polyprotein - NS5A | 1467,7906 | 212 - 225 | 72 | 0.008 | ETAARRLARGSPPS |
| A9JKP2 | Polyprotein - NS5A | 1870,9609 | 213 - 231 | 72 | 0.008 | TAARRLARGSPPSEASSSA |
| A9JKP2 | Polyprotein - NS5A | 1157,5676 | 218 - 229 | 72 | 0.008 | LARGSPPSEASS |
| A9JKP2 | Polyprotein - NS5A | 1244,5997 | 218 - 230 | 72 | 0.008 | LARGSPPSEASSS |
| A9JKP2 | Polyprotein - NS5A | 1131,5156 | 220 - 231 | 72 | 0.008 | RGSPPSEASSSA |
| A9JKP2 | Polyprotein - NS5A | 1540,7668 | 233 - 246 | 72 | 0.008 | QLSAPSLKATCQTH |
| A9JKP2 | Polyprotein - NS5A | 714,4177 | 259 - 263 | 72 | 0.008 | LLWRQ |
| A9JKP2 | Polyprotein - NS5A | 1879,9058 | 262 - 277 | 72 | 0.008 | RQEMGSNITRVESETK** |
| A9JKP2 | Polyprotein - NS5A | 2820,4215 | 263 - 287 | 72 | 0.008 | QEMGSNITRVESETKVVILDSFEPL |
| A9JKP2 | Polyprotein - NS5A | 832,4290 | 269 - 275 | 72 | 0.008 | ITRVESE |
| A9JKP2 | Polyprotein - NS5A | 1774,8737 | 284 - 300 | 72 | 0.008 | FEPLRAETGDAELSVAA |
| A9JKP2 | Polyprotein - NS5A | 1273,6853 | 298 - 308 | 72 | 0.008 | VAAECFKKPPK |
| A9JKP2 | Polyprotein - NS5A | 807,3949 | 301 - 306 | 72 | 0.008 | ECFKKP |
| A9JKP2 | Polyprotein - NS5A | 851,5017 | 313 - 319 | 72 | 0.008 | LPIWARP |
| A9JKP2 | Polyprotein - NS5A | 919,4552 | 315 - 321 | 72 | 0.008 | IWARPDY |
| A9JKP2 | Polyprotein - NS5A | 1848,9522 | 322 - 337 | 72 | 0.008 | NPPLLDRWKAPDYAPP |
| A9JKP2 | Polyprotein - NS5A | 1469,7667 | 323 - 334 | 72 | 0.008 | PPLLDRWKAPDY |
| A9JKP2 | Polyprotein - NS5A | 2503,2067 | 327 - 349 | 72 | 0.008 | DRWKAPDYAPPTVHGCALPPQGA |
| A9JKP2 | Polyprotein - NS5A | 729,3333 | 331 - 337 | 72 | 0.008 | APDYAPP |
| A9JKP2 | Polyprotein - NS5A | 1789,8458 | 331 - 347 | 72 | 0.008 | APDYAPPTVHGCALPPQ |
| A9JKP2 | Polyprotein - NS5A | 1594,7926 | 336 - 351 | 72 | 0.008 | PPTVHGCALPPQGAPP |
| A9JKP2 | Polyprotein - NS5A | 844,4290 | 361 - 368 | 72 | 0.008 | IQLDGSNV |
| A9JKP2 | Polyprotein - NS5A | 843,5066 | 368 - 376 | 72 | 0.008 | VSAALAVLT |
| A9JKP2 | Polyprotein - NS5A | 1277,6867 | 372 - 383 | 72 | 0.008 | LAVLTEKSFPSS |
| A9JKP2 | Polyprotein - NS5A | 908,4603 | 377 - 384 | 72 | 0.008 | EKSFPSSK |
| A9JKP2 | Polyprotein - NS5A | 748,3755 | 379 - 385 | 72 | 0.008 | SFPSSKP |
| A9JKP2 | Polyprotein - NS5A | 1030,5295 | 398 - 407 | 72 | 0.008 | QSSTTSKVPP |
| A9JKP2 | Polyprotein - NS5A | 912,4917 | 401 - 409 | 72 | 0.008 | TTSKVPPSP |
| A9JKP2 | Polyprotein - NS5A | 1040,5138 | 403 - 413 | 72 | 0.008 | SKVPPSPGGES |
| A9JKP2 | Polyprotein - NS5A | 953,4818 | 404 - 413 | 72 | 0.008 | KVPPSPGGES |
| A9JKP2 | Polyprotein - NS5A | 1230,5074 | 419 - 430 | 72 | 0.008 | SSMPPLEGEPGD** |
| A9JKP2 | Polyprotein - NS5A | 1956,8047 | 422 - 439 | 72 | 0.008 | PPLEGEPGDPDLSCDSWS |
| A9JKP2 | Polyprotein - NS5A | 705,2640 | 431 - 436 | 72 | 0.008 | PDLSCD |
| Q6TZ17 | NS5A protein | 1183,5986 | 1 - 11 | 77 | 0.0026 | VGLHQYPVGS |
| Q6TZ17 | NS5A protein | 1296,6826 | 1 - 12 | 77 | 0.008 | VGLHQYPVGSQL |
| Q6TZ17 | NS5A protein | 1454,6976 | 2 - 14 | 77 | 0.008 | GLHQYPVGSQLPC |
| Q6TZ17 | NS5A protein | 2958,3813 | 3 - 28 | 77 | 0.0026 | LHQYPVGSQLPCEPEPDVTMLTSMLT** |
| Q6TZ17 | NS5A protein | 1471,6653 | 6 - 18 | 77 | 0.0026 | YPVGSQLPCEPEP |
| Q6TZ17 | NS5A protein | 1423,6290 | 7 - 19 | 77 | 0.008 | PVGSQLPCEPEPD |
| Q6TZ17 | NS5A protein | 1976,9071 | 19 - 36 | 77 | 0.008 | DVTMLTSMLTDPSHITAE** |
| Q6TZ17 | NS5A protein | 1011,4981 | 20 - 28 | 77 | 0.008 | VTMLTSMLT** |
| Q6TZ17 | NS5A protein | 1310,6098 | 20 - 31 | 77 | 0.008 | VTMLTSMLTDPS** |
| Q6TZ17 | NS5A protein | 2049,9599 | 20 - 38 | 77 | 0.008 | VTMLTSMLTDPSHITAETA** |
| Q6TZ17 | NS5A protein | 1344,6418 | 22 - 33 | 77 | 0.008 | MLTSMLTDPSHI |
| Q6TZ17 | NS5A protein | 1762,8117 | 22 - 37 | 77 | 0.0026 | MLTSMLTDPSHITAET** |
| Q6TZ17 | NS5A protein | 979,4532 | 23 - 31 | 77 | 0.008 | LTSMLTDPS** |
| Q6TZ17 | NS5A protein | 1518,6872 | 24 - 37 | 77 | 0.0026 | TSMLTDPSHITAET** |
| Q6TZ17 | NS5A protein | 2210,1590 | 26 - 44 | 77 | 0.008 | MLTDPSHITAETAKRRLDR |
| Q6TZ17 | NS5A protein | 953,4818 | 27 - 35 | 77 | 0.008 | LTDPSHITA |
| Q6TZ17 | NS5A protein | 899,5413 | 39 - 45 | 77 | 0.0026 | KRRLDRG |
| Q6TZ17 | NS5A protein | 1870,9973 | 39 - 56 | 77 | 0.0026 | KRRLDRGSPPSLASSSAS |
| Q6TZ17 | NS5A protein | 1870,9609 | 40 - 57 | 77 | 0.008 | RRLDRGSPPSLASSSASQ |
| Q6TZ17 | NS5A protein | 1098,5669 | 42 - 52 | 77 | 0.0026 | LDRGSPPSLAS |
| Q6TZ17 | NS5A protein | 1044,5200 | 44 - 54 | 77 | 0.0026 | RGSPPSLASSS |
| Q6TZ17 | NS5A protein | 1030,4931 | 47 - 57 | 77 | 0.0026 | PPSLASSSASQ |
| Q6TZ17 | NS5A protein | 1046,5244 | 53 - 63 | 77 | 0.0026 | SSASQLSAPSL |
| Q6TZ17 | NS5A protein | 1607,7825 | 53 - 68 | 77 | 0.0026 | SSASQLSAPSLKATCT |
| Q6TZ17 | NS5A protein | 2214,0699 | 54 - 74 | 77 | 0.008 | SASQLSAPSLKATCTTRHDSP |
| Q6TZ17 | NS5A protein | 2171,0277 | 56 - 75 | 77 | 0.008 | A.SQLSAPSLKATCTTRHDSPD.A |
| Q6TZ17 | NS5A protein | 933,4590 | 59 - 67 | 77 | 0.008 | SAPSLKATC |
| Q6TZ17 | NS5A protein | 2142,0011 | 59 - 78 | 77 | 0.008 | SAPSLKATCTTRHDSPDADL |
| Q6TZ17 | NS5A protein | 1226,5528 | 68 - 78 | 77 | 0.0026 | TTRHDSPDADL |
| Q6TZ17 | NS5A protein | 1879,9276 | 68 - 84 | 77 | 0.008 | TTRHDSPDADLIEANLL |
| Q6TZ17 | NS5A protein | 731,2974 | 72 - 78 | 77 | 0.0026 | DSPDADL |
| Q6TZ17 | NS5A protein | 3173,4935 | 72 - 99 | 77 | 0.0026 | DSPDADLIEANLLWRQEMGGNITRVESE** |
| Q6TZ17 | NS5A protein | 1515,7504 | 79 - 91 | 77 | 0.0026 | IEANLLWRQEMGG |
| Q6TZ17 | NS5A protein | 1157,5829 | 80 - 88 | 77 | 0.0026 | EANLLWRQE |
| Q6TZ17 | NS5A protein | 1273,6237 | 81 - 91 | 77 | 0.008 | ANLLWRQEMGG |
| Q6TZ17 | NS5A protein | 1031,5222 | 83 - 90 | 77 | 0.0026 | LLWRQEMG |
| Q6TZ17 | NS5A protein | 877,4116 | 84 - 89 | 77 | 0.0026 | LWRQEM** |
| Q6TZ17 | NS5A protein | 1774,8784 | 84 - 98 | 77 | 0.0026 | LWRQEMGGNITRVES |
| Q6TZ17 | NS5A protein | 748,3326 | 85 - 89 | 77 | 0.0026 | WRQEM |
| Q6TZ17 | NS5A protein | 2231,1117 | 85 - 103 | 77 | 0.0026 | WRQEMGGNITRVESENKVV |
| Q6TZ17 | NS5A protein | 1232,5819 | 87 - 97 | 77 | 0.0026 | QEMGGNITRVE |
| Q6TZ17 | NS5A protein | 1789,8628 | 87 - 102 | 77 | 0.0026 | QEMGGNITRVESENKV |
| Q6TZ17 | NS5A protein | 832,4290 | 93 - 99 | 77 | 0.0026 | ITRVESE |
| Q6TZ17 | NS5A protein | 807,4014 | 103 - 109 | 77 | 0.0026 | VILDSFD |
| Q6TZ17 | NS5A protein | 919,4399 | 106 - 113 | 77 | 0.008 | DSFDPLRA |
| Q6TZ17 | NS5A protein | 2863,5264 | 109 - 132 | 77 | 0.0026 | DPLRAEEDEREVSVPAEILRRTRK |
| Q6TZ17 | NS5A protein | 1244,5997 | 111 - 120 | 77 | 0.008 | LRAEEDEREV |
| Q6TZ17 | NS5A protein | 1131,5156 | 112 - 120 | 77 | 0.0026 | RAEEDEREV |
| Q6TZ17 | NS5A protein | 1129,5251 | 115 - 124 | 77 | 0.0026 | EDEREVSVPA |
| Q6TZ17 | NS5A protein | 714,4177 | 131 - 136 | 77 | 0.0026 | RKFPPA |
| Q6TZ17 | NS5A protein | 1597,8405 | 133 - 146 | 77 | 0.0026 | FPPALPIWARPGYN |
| Q6TZ17 | NS5A protein | 851,5017 | 137 - 143 | 77 | 0.0026 | LPIWARP |
| Q6TZ17 | NS5A protein | 908,5232 | 137 - 144 | 77 | 0.008 | LPIWARPG |
| Q6TZ17 | NS5A protein | 748,3656 | 140 - 145 | 77 | 0.0026 | WARPGY |
| Q6TZ17 | NS5A protein | 2499,2223 | 140 - 160 | 77 | 0.0026 | WARPGYNPPLLESWKDPDYVP |
| Q6TZ17 | NS5A protein | 912,4817 | 142 - 149 | 77 | 0.008 | RPGYNPPL |
| Q6TZ17 | NS5A protein | 844,4330 | 145 - 151 | 77 | 0.0026 | YNPPLLE |
| Q6TZ17 | NS5A protein | 889,4545 | 149 - 155 | 77 | 0.0026 | LLESWKD |
| Q6TZ17 | NS5A protein | 843,4967 | 173 - 180 | 77 | 0.0026 | APPIPPPR |
| Q6TZ17 | NS5A protein | 3024,6567 | 180 - 207 | 77 | 0.0026 | RRKRTVVLTESTVSSALAELATKTFSSS |
| Q6TZ17 | NS5A protein | 1277,6827 | 183 - 194 | 77 | 0.008 | RTVVLTESTVSS |
| Q6TZ17 | NS5A protein | 1505,7825 | 184 - 198 | 77 | 0.0026 | TVVLTESTVSSALAE |
| Q6TZ17 | NS5A protein | 2503,2177 | 187 - 211 | 77 | 0.0026 | LTESTVSSALAELATKTFSSSESSA |
| Q6TZ17 | NS5A protein | 1061,5604 | 191 - 201 | 77 | 0.0026 | TVSSALAELAT |
| Q6TZ17 | NS5A protein | 1498,7515 | 193 - 207 | 77 | 0.0026 | SSALAELATKTFSSS |
| Q6TZ17 | NS5A protein | 1540,7620 | 194 - 208 | 77 | 0.0026 | SALAELATKTFSSSE |
| Q6TZ17 | NS5A protein | 1540,7620 | 195 - 209 | 77 | 0.0026 | ALAELATKTFSSSES |
| Q6TZ17 | NS5A protein | 1469,7249 | 196 - 209 | 77 | 0.008 | LAELATKTFSSSES |
| Q6TZ17 | NS5A protein | 1832,8276 | 201 - 219 | 77 | 0.0026 | TKTFSSSESSAVDSGTATA |
| Q6TZ17 | NS5A protein | 784,3603 | 202 - 208 | 77 | 0.0026 | KTFSSSE |
| Q6TZ17 | NS5A protein | 729,2817 | 204 - 210 | 77 | 0.0026 | FSSSESS |
| Q6TZ17 | NS5A protein | 1096,4520 | 206 - 217 | 77 | 0.0026 | SSESSAVDSGTA |
| Q6TZ17 | NS5A protein | 975,4509 | 210 - 220 | 77 | 0.008 | SAVDSGTATAP |
| Q6TZ17 | NS5A protein | 1956,7531 | 224 - 242 | 77 | 0.008 | PSDNGDTGSDVESYSSMPP** |
| Q6TZ17 | NS5A protein | 1756,6734 | 226 - 242 | 77 | 0.0026 | DNGDTGSDVESYSSMPP |
| Q6TZ17 | NS5A protein | 1213,4809 | 232 - 242 | 77 | 0.0026 | SDVESYSSMPP** |
| Q6TZ17 | NS5A protein | 718,2480 | 235 - 240 | 77 | 0.0026 | ESYSSM** |
| Q6TZ17 | NS5A protein | 1230,5074 | 238 - 249 | 77 | 0.0026 | SSMPPLEGEPGD** |
| Q1HFC6 | NS5A protein | 1976,9455 | 4 - 18 | 73 | 0.0061 | WLRDVWDWICTVLTD |
| Q1HFC6 | NS5A protein | 877,3793 | 8 - 13 | 73 | 0.0061 | VWDWIC |
| Q1HFC6 | NS5A protein | 2210,0871 | 8 - 24 | 73 | 0.0061 | VWDWICTVLTDFKTWLQ |
| Q1HFC6 | NS5A protein | 891,4524 | 11 - 17 | 73 | 0.0061 | WICTVLT |
| Q1HFC6 | NS5A protein | 3024,6048 | 19 - 43 | 73 | 0.0061 | FKTWLQTKLLPQLPGIPFFSCQRGY |
| Q1HFC6 | NS5A protein | 1505,8970 | 22 - 34 | 73 | 0.0061 | WLQTKLLPQLPGI |
| Q1HFC6 | NS5A protein | 1597,9232 | 24 - 37 | 73 | 0.0061 | QTKLLPQLPGIPFF |
| Q1HFC6 | NS5A protein | 1540,7497 | 31 - 43 | 73 | 0.0061 | Q.LPGIPFFSCQRGY.R |
| Q1HFC6 | NS5A protein | 2141,9847 | 38 - 55 | 73 | 0.0061 | SCQRGYRGVWRGDGIMQT** |
| Q1HFC6 | NS5A protein | 1277,6378 | 41 - 51 | 73 | 0.0061 | RGYRGVWRGDG |
| Q1HFC6 | NS5A protein | 844,4304 | 44 - 50 | 73 | 0.0061 | RGVWRGD |
| Q1HFC6 | NS5A protein | 1273,6350 | 44 - 54 | 73 | 0.0061 | RGVWRGDGIMQ |
| Q1HFC6 | NS5A protein | 3173,4740 | 44 - 72 | 73 | 0.0061 | RGVWRGDGIMQTTCPCGAQITGHVKNGSM |
| Q1HFC6 | NS5A protein | 2499,2475 | 59 - 81 | 73 | 0.0061 | CGAQITGHVKNGSMRIVGPKTWS** |
| Q1HFC6 | NS5A protein | 1454,7412 | 60 - 73 | 73 | 0.0061 | GAQITGHVKNGSMR |
| Q1HFC6 | NS5A protein | 2050,1106 | 60 - 79 | 73 | 0.0061 | GAQITGHVKNGSMRIVGPKT |
| Q1HFC6 | NS5A protein | 1467,7616 | 64 - 77 | 73 | 0.0061 | TGHVKNGSMRIVGP** |
| Q1HFC6 | NS5A protein | 1040,5549 | 66 - 74 | 73 | 0.0061 | HVKNGSMRI |
| Q1HFC6 | NS5A protein | 919,4909 | 68 - 75 | 73 | 0.0061 | KNGSMRIV** |
| Q1HFC6 | NS5A protein | 832,4225 | 69 - 76 | 73 | 0.0061 | NGSMRIVG |
| Q1HFC6 | NS5A protein | 1344,6972 | 69 - 80 | 73 | 0.0061 | NGSMRIVGPKTW |
| Q1HFC6 | NS5A protein | 1044,5750 | 70 - 79 | 73 | 0.0061 | GSMRIVGPKT |
| Q1HFC6 | NS5A protein | 2171,0735 | 71 - 89 | 73 | 0.0061 | SMRIVGPKTWSNAWHGTFP |
| Q1HFC6 | NS5A protein | 2820,3806 | 72 - 96 | 73 | 0.0061 | MRIVGPKTWSNAWHGTFPINAYTTG** |
| Q1HFC6 | NS5A protein | 1953,0010 | 73 - 89 | 73 | 0.0061 | RIVGPKTWSNAWHGTFP |
| Q1HFC6 | NS5A protein | 1832,8482 | 81 - 97 | 73 | 0.0061 | SNAWHGTFPINAYTTGP |
| Q1HFC6 | NS5A protein | 1226,5833 | 82 - 92 | 73 | 0.0061 | NAWHGTFPINA |
| Q1HFC6 | NS5A protein | 784,3868 | 85 - 91 | 73 | 0.0061 | HGTFPIN |
| Q1HFC6 | NS5A protein | 1756,8090 | 89 - 105 | 73 | 0.0061 | PINAYTTGPCTPSPAPN |
| Q1HFC6 | NS5A protein | 1096,4859 | 90 - 99 | 73 | 0.0061 | INAYTTGPCT |
| Q1HFC6 | NS5A protein | 1357,6779 | 100 - 111 | 73 | 0.0061 | PSPAPNYSRALW |
| Q1HFC6 | NS5A protein | 2503,2495 | 100 - 121 | 73 | 0.0061 | PSPAPNYSRALWRVAAEEYVEV |
| Q1HFC6 | NS5A protein | 908,4504 | 105 - 111 | 73 | 0.0061 | NYSRALW |
| Q1HFC6 | NS5A protein | 1108,5288 | 113 - 122 | 73 | 0.0061 | VAAEEYVEVT |
| Q1HFC6 | NS5A protein | 2231,0205 | 116 - 134 | 73 | 0.0061 | EEYVEVTRVGDFHYVTGMT |
| Q1HFC6 | NS5A protein | 1349,6728 | 121 - 132 | 73 | 0.0061 | VTRVGDFHYVTG |
| Q1HFC6 | NS5A protein | 1296,5921 | 123 - 133 | 73 | 0.0061 | RVGDFHYVTGM** |
| Q1HFC6 | NS5A protein | 1498,6908 | 135 - 147 | 73 | 0.0061 | TDNLKCPCQVPAP |
| Q1HFC6 | NS5A protein | 933,4596 | 143 - 150 | 73 | 0.0061 | QVPAPEFF |
| Q1HFC6 | NS5A protein | 807,3803 | 145 - 151 | 73 | 0.0061 | PAPEFFT |
| Q1HFC6 | NS5A protein | 1310,6507 | 148 - 158 | 73 | 0.0061 | EFFTEVDGVRL |
| Q1HFC6 | NS5A protein | 1870,9472 | 151 - 166 | 73 | 0.0061 | TEVDGVRLHRYAPACK |
| Q1HFC6 | NS5A protein | 794,4399 | 153 - 159 | 73 | 0.0061 | VDGVRLH |
| Q1HFC6 | NS5A protein | 851,4726 | 154 - 160 | 73 | 0.0061 | DGVRLHR |
| Q1HFC6 | NS5A protein | 899,5090 | 155 - 161 | 73 | 0.0061 | GVRLHRY |
| Q1HFC6 | NS5A protein | 1031,5474 | 161 - 169 | 73 | 0.0061 | YAPACKPLL |
| Q1HFC6 | NS5A protein | 1153,6277 | 162 - 171 | 73 | 0.0061 | APACKPLLRE |
| Q1HFC6 | NS5A protein | 777,4021 | 174 - 180 | 73 | 0.0061 | TFQVGLN |
| Q1HFC6 | NS5A protein | 2863,3508 | 182 - 207 | 73 | 0.0061 | YLVGSQLPCEPEPDVTVLTSMLTDPS** |
| Q1HFC6 | NS5A protein | 1098,5379 | 183 - 192 | 73 | 0.0061 | LVGSQLPCEP |
| Q1HFC6 | NS5A protein | 1469,6708 | 186 - 198 | 73 | 0.0061 | SQLPCEPEPDVTV |
| Q1HFC6 | NS5A protein | 2214,0726 | 194 - 214 | 73 | 0.0061 | PDVTVLTSMLTDPSHITAETA** |
| Q1HFC6 | NS5A protein | 979,4896 | 197 - 205 | 73 | 0.0061 | TVLTSMLTD |
| Q1HFC6 | NS5A protein | 1518,6872 | 200 - 213 | 73 | 0.0061 | TSMLTDPSHITAET** |
| Q1HFC6 | NS5A protein | 2214,1426 | 200 - 219 | 73 | 0.0061 | TSMLTDPSHITAETAKRRLA** |
| Q1HFC6 | NS5A protein | 953,4818 | 203 - 211 | 73 | 0.0061 | LTDPSHITA |
| Q1HFC6 | NS5A protein | 1030,4931 | 223 - 233 | 73 | 0.0061 | PPSLASSSASQ |
| Q1HFC6 | NS5A protein | 1046,5244 | 229 - 239 | 73 | 0.0061 | SSASQLSAPSL |
| Q1HFC6 | NS5A protein | 1607,7825 | 229 - 244 | 73 | 0.0061 | SSASQLSAPSLKATCT |
| Q1HFC6 | NS5A protein | 1879,9276 | 244 - 260 | 73 | 0.0061 | TTRHDSPDADLIEANLL |
| Q1HFC6 | NS5A protein | 731,2974 | 248 - 254 | 73 | 0.0061 | DSPDADL |
| Q1HFC6 | NS5A protein | 1515,7504 | 255 - 267 | 73 | 0.0061 | IEANLLWRQEMGG |
| Q1HFC6 | NS5A protein | 1157,5829 | 256 - 264 | 73 | 0.0061 | EANLLWRQE |
| Q1HFC6 | NS5A protein | 714,4177 | 259 - 263 | 73 | 0.0061 | LLWRQ |
| Q1HFC6 | NS5A protein | 748,3326 | 261 - 265 | 73 | 0.0061 | WRQEM.G |
| Q1HFC6 | NS5A protein | 1232,5819 | 263 - 273 | 73 | 0.0061 | QEMGGNITRVE |
| Q1HFC6 | NS5A protein | 1789,8628 | 263 - 278 | 73 | 0.0061 | QEMGGNITRVESENKV |
| Q1HFC6 | NS5A protein | 2958,4934 | 267 - 292 | 73 | 0.0061 | GNITRVESENKVVILDSFEPLRAEED |
| Q1HFC6 | NS5A protein | 1131,5156 | 288 - 296 | 73 | 0.0061 | RAEEDEREV |
| Q1HFC6 | NS5A protein | 1244,5997 | 287 - 296 | 73 | 0.0061 | LRAEEDEREV |
| Q1HFC6 | NS5A protein | 975,4509 | 291 - 298 | 73 | 0.0061 | EDEREVSI |
| Q1HFC6 | NS5A protein | 770,4327 | 309 - 315 | 73 | 0.0061 | FPQALPV |
| Q1HFC6 | NS5A protein | 1183,6502 | 309 - 318 | 73 | 0.0061 | FPQALPVWAR |
| Q1HFC6 | NS5A protein | 1848,9886 | 310 - 325 | 73 | 0.0061 | PQALPVWARPDYNPLL |
| Q1HFC6 | NS5A protein | 2281,2259 | 312 - 330 | 73 | 0.0061 | ALPVWARPDYNPLLLETWK |
| Q1HFC6 | NS5A protein | 1774,9077 | 333 - 348 | 73 | 0.0061 | DYVPPVVHGCPLPPTK |
| Q1HFC6 | NS5A protein | 912,4739 | 342 - 349 | 73 | 0.0061 | CPLPPTKT |
| Q1HFC6 | NS5A protein | 1061,5604 | 363 - 373 | 73 | 0.0061 | LTESTVASALA |
| Q1HFC6 | NS5A protein | 1565,8301 | 367 - 382 | 73 | 0.0061 | TVASALAELATKTFGS |
| Q1HFC6 | NS5A protein | 843,4702 | 368 - 376 | 73 | 0.0061 | VASALAELA |
| Q1HFC6 | NS5A protein | 1213,5463 | 378 - 389 | 73 | 0.0061 | KTFGSSESSAVD |
| Q1HFC6 | NS5A protein | 1762,7493 | 381 - 399 | 73 | 0.0061 | GSSESSAVDSGTATAPPDQ |
| Q1HFC6 | NS5A protein | 1423,6177 | 410 - 422 | 73 | 0.0061 | VESYSSMPPLEGE |
| Q1HFC6 | NS5A protein | 718,2480 | 411 - 416 | 73 | 0.0061 | ESYSSM** |
| Q1HFC6 | NS5A protein | 1230,5074 | 414 - 425 | 73 | 0.0061 | SSMPPLEGEPGD** |
| Q1HFC6 | NS5A protein | 729,3367 | 415 - 421 | 73 | 0.0061 | SMPPLEG |
| Q1HFC6 | NS5A protein | 1756,7428 | 418 - 434 | 73 | 0.0061 | PLEGEPGDPDLSDGSWS |
| M9UX90 | NS5A protein (3b) | 2124,1111 | 13 - 30 | 68 | 0.019 | CTVLSDFKTWLSAKIMPK |
| M9UX90 | NS5A protein (3b) | 1061,5579 | 21 - 29 | 68 | 0.019 | TWLSAKIMP.K** |
| M9UX90 | NS5A protein (3b) | 1514,8353 | 23 - 36 | 68 | 0.019 | LSAKIMPKMPGVPF |
| M9UX90 | NS5A protein (3b) | 916,4874 | 26 - 33 | 68 | 0.019 | KIMPKMPG** |
| M9UX90 | NS5A protein (3b) | 2214,0793 | 34 - 52 | 68 | 0.019 | VPFFSCQRGYKGVWRGDGV |
| M9UX90 | NS5A protein (3b) | 1471,7565 | 66 - 79 | 68 | 0.019 | HVKNGSMRIAGSSL** |
| M9UX90 | NS5A protein (3b) | 1976,8543 | 70 - 87 | 68 | 0.019 | GSMRIAGSSLCANMWYGT** |
| M9UX90 | NS5A protein (3b) | 794,4075 | 106 - 111 | 68 | 0.019 | YSRALW |
| M9UX90 | NS5A protein (3b) | 2290,0940 | 132 - 152 | 68 | 0.019 | GATNDGLKIPCQVPAPEFFTE |
| M9UX90 | NS5A protein (3b) | 784,3643 | 148 - 153 | 68 | 0.019 | EFFTEL |
| M9UX90 | NS5A protein (3b) | 1756,8090 | 178 - 193 | 68 | 0.019 | GLHSYAIGSQLPCEPE |
| M9UX90 | NS5A protein (3b) | 2191,0831 | 192 - 212 | 68 | 0.019 | PEPDVAVLTSMLQDPSHITAA |
| M9UX90 | NS5A protein (3b) | 1998,0204 | 198 - 216 | 68 | 0.019 | VLTSMLQDPSHITAATAAR** |
| M9UX90 | NS5A protein (3b) | 1833,9921 | 204 - 220 | 68 | 0.019 | QDPSHITAATAARRLAR |
| M9UX90 | NS5A protein (3b) | 1226,6554 | 239 - 248 | 68 | 0.019 | LKATCQTHRL |
| M9UX90 | NS5A protein (3b) | 844,4304 | 246 - 252 | 68 | 0.019 | HRLHPDA |
| M9UX90 | NS5A protein (3b) | 2644,2942 | 264 - 286 | 68 | 0.019 | DMGSYITRIESDTKVLILDSFEP** |
| M9UX90 | NS5A protein (3b) | 1273,7242 | 270 - 280 | 68 | 0.019 | TRIESDTKVLI |
| M9UX90 | NS5A protein (3b) | 1501,8352 | 270 - 282 | 68 | 0.019 | TRIESDTKVLILD |
| M9UX90 | NS5A protein (3b) | 1244,6864 | 272 - 282 | 68 | 0.019 | IESDTKVLILD |
| M9UX90 | NS5A protein (3b) | 2318,1529 | 279 - 298 | 68 | 0.019 | LILDSFEPLRVEEDDTELSV |
| M9UX90 | NS5A protein (3b) | 1064,5325 | 297 - 305 | 68 | 0.019 | SVPAECFKK |
| M9UX90 | NS5A protein (3b) | 975,4848 | 300 - 307 | 68 | 0.019 | AECFKKPP |
| M9UX90 | NS5A protein (3b) | 1604,9442 | 304 - 317 | 68 | 0.019 | KKPPKYPPALPIWA |
| M9UX90 | NS5A protein (3b) | 1879,9985 | 306 - 321 | 68 | 0.019 | PPKYPPALPIWARPDY |
| M9UX90 | NS5A protein (3b) | 1435,7500 | 321 - 332 | 68 | 0.019 | YNPPLLPPWKDP |
| M9UX90 | NS5A protein (3b) | 1061,5910 | 324 - 332 | 68 | 0.019 | PLLPPWKDP |
| M9UX90 | NS5A protein (3b) | 1230,5921 | 329 - 338 | 68 | 0.019 | WKDPTYEPPV |
| M9UX90 | NS5A protein (3b) | 1183,6826 | 346 - 356 | 68 | 0.019 | PTRPAPVPPPR |
| M9UX90 | NS5A protein (3b) | 1272,7190 | 370 - 381 | 68 | 0.019 | RALAALAEKSFP |
| M9UX90 | NS5A protein (3b) | 2030,0796 | 370 - 389 | 68 | 0.019 | RALAALAEKSFPSTKPEGTG |
| M9UX90 | NS5A protein (3b) | 714,3548 | 381 - 387 | 68 | 0.019 | PSTKPEG |
| M9UX90 | NS5A protein (3b) | 877,4545 | 375 - 382 | 68 | 0.019 | LAEKSFPS |
| M9UX90 | NS5A protein (3b) | 1939,7993 | 406 - 424 | 68 | 0.019 | SPETGEGSDAESYSSMPPL |
| M9UX90 | NS5B protein (3b) | 1756,8553 | 464 - 479 | 68 | 0.019 | TPCSAEEEKLPISPLS |
| M9UX90 | NS5B protein (3b) | 1969,0745 | 470 - 486 | 68 | 0.019 | EEKLPISPLSNSLLRHH |
| M9UX90 | NS5B protein (3b) | 829,4545 | 475 - 482 | 68 | 0.019 | ISPLSNSL |
| M9UX90 | NS5B protein (3b) | 1098,6397 | 475 - 484 | 68 | 0.019 | ISPLSNSLLR |
| M9UX90 | NS5B protein (3b) | 1376,7484 | 491 - 503 | 68 | 0.019 | STSSRSAAARQKK |
| M9UX90 | NS5B protein (3b) | 1298,8034 | 517 - 527 | 68 | 0.019 | KKALKEVKERA |
| M9UX90 | NS5B protein (3b) | 1547,8420 | 526 - 539 | 68 | 0.019 | RASGVKGRLLSFEE |
| M9UX90 | NS5B protein (3b) | 3685,8662 | 574 - 606 | 68 | 0.019 | VWEDLLEDNTTPIPTTIMAKNEVFSVNPVKGGR** |
| M9UX90 | NS5B protein (3b) | 787,4150 | 585 - 591 | 68 | 0.019 | PIPTTIM** |
| M9UX90 | NS5B protein (3b) | 2214,1718 | 585 - 605 | 68 | 0.019 | PIPTTIMAKNEVFSVNPVKGG** |
| M9UX90 | NS5B protein (3b) | 720,3476 | 590 - 595 | 68 | 0.019 | IMAKNE** |
| M9UX90 | NS5B protein (3b) | 1994,1901 | 603 - 620 | 68 | 0.019 | KGGRKPARLIVYPDLGVR |
| M9UX90 | NS5B protein (3b) | 1851,1207 | 606 - 621 | 68 | 0.019 | RKPARLIVYPDLGVRV |
| M9UX90 | NS5B protein (3b) | 1402,7643 | 611 - 622 | 68 | 0.019 | LIVYPDLGVRVC |
| M9UX90 | NS5B protein (3b) | 1789,9873 | 617 - 631 | 68 | 0.019 | LGVRVCEKRALYDVI |
| M9UX90 | NS5B protein (3b) | 1148,6441 | 628 - 637 | 68 | 0.019 | YDVIQKLSIA |
| M9UX90 | NS5B protein (3b) | 1147,6271 | 631 - 641 | 68 | 0.019 | IQKLSIATMGA** |
| M9UX90 | NS5B protein (3b) | 2210,0942 | 639 - 657 | 68 | 0.019 | MGAAYGFQYSPKQRVEHLL** |
| M9UX90 | NS5B protein (3b) | 1953,0618 | 648 - 663 | 68 | 0.019 | SPKQRVEHLLKMWSSK |
| M9UX90 | NS5B protein (3b) | 1608,8698 | 657 - 670 | 68 | 0.019 | LKMWSSKKTPLGFS |
| M9UX90 | NS5B protein (3b) | 912,5028 | 698 - 705 | 68 | 0.019 | EPEARKAI |
| M9UX90 | NS5B protein (3b) | 1213,5801 | 710 - 719 | 68 | 0.019 | ERLYIGGPMY** |
| M9UX90 | NS5B protein (3b) | 2503,1631 | 714 - 735 | 68 | 0.019 | IGGPMYNSKGLQCGYRRCRASG** |
| M9UX90 | NS5B protein (3b) | 2015,0034 | 734 - 752 | 68 | 0.019 | SGVLPTSFGNTVTCYIKAT |
| M9UX90 | NS5B protein (3b) | 1096,5587 | 745 - 754 | 68 | 0.019 | VTCYIKATAA |
| M9UX90 | NS5B protein (3b) | 1226,6731 | 751 - 763 | 68 | 0.019 | ATAASRAAGLKNP |
| M9UX90 | NS5B protein (3b) | 843,4926 | 753 - 761 | 68 | 0.019 | AASRAAGLK |
| M9UX90 | NS5B protein (3b) | 1271,7350 | 756 - 767 | 68 | 0.019 | RAAGLKNPSFLV |
| M9UX90 | NS5B protein (3b) | 1115,6339 | 757 - 767 | 68 | 0.019 | AAGLKNPSFLV |
| M9UX90 | NS5B protein (3b) | 2430,0903 | 777 - 797 | 68 | 0.019 | ESCGVEEDRSALRAFTEAMTR** |
| M9UX90 | NS5B protein (3b) | 1956,8669 | 779 - 795 | 68 | 0.019 | CGVEEDRSALRAFTEAM** |
| M9UX90 | NS5B protein (3b) | 2492,1377 | 800 - 823 | 68 | 0.019 | APPGDAPQATYDLELITSCSSNVS |
| M9UX90 | NS5B protein (3b) | 2379,0987 | 822 - 841 | 68 | 0.019 | VSVACDGMGKRYYYLTRDPE |
| M9UX90 | NS5B protein (3b) | 1110,5418 | 850 - 859 | 68 | 0.019 | ETARHTPVNS |
| M9UX90 | NS5B protein (3b) | 841,4810 | 868 - 874 | 68 | 0.019 | APTIWVR |
| M9UX90 | NS5B protein (3b) | 1244,6774 | 869 - 878 | 68 | 0.019 | PTIWVRMVLM |
| M9UX90 | NS5B protein (3b) | 855,5039 | 935 - 942 | 68 | 0.019 | NRVAGALR |
| M9UX90 | NS5B protein (3b) | 1031,5777 | 947 - 954 | 68 | 0.019 | PPLRAWRH |
| M9UX90 | NS5B protein (3b) | 1349,8394 | 985 - 997 | 68 | 0.019 | KTRLTPLPAAGLL |
| M9UX90 | NS5B protein (3b) | 764,3858 | 1002 - 1008 | 68 | 0.019 | WFTVGVG |
| M9UX90 | NS5B protein (3b) | 1068,5901 | 1015 - 1023 | 68 | 0.019 | SVSRARTRH |
| M9UX90 | NS5B protein (3b) | 1466,9476 | 1028 - 1041 | 68 | 0.019 | LLLLTVGVGIFLLP |
| G8CSB7 | NS5B protein | 1871,0788 | 4 - 17 | 86 | 0.00028 | KQRVDRLLKMWRAR** |
| G8CSB7 | NS5B protein | 2367,2456 | 7 - 25 | 86 | 0.00028 | VDRLLKMWRARKNPMGFSY |
| G8CSB7 | NS5B protein | 748,4054 | 11 - 15 | 86 | 0.00028 | LKMWR** |
| G8CSB7 | NS5B protein | 2191,0204 | 14 - 30 | 86 | 0.00028 | WRARKNPMGFSYDTRCF |
| G8CSB7 | NS5B protein | 2409,0743 | 14 - 32 | 86 | 0.00028 | WRARKNPMGFSYDTRCFDS** |
| G8CSB7 | NS5B protein | 1098,5280 | 17 - 25 | 86 | 0.00028 | RKNPMGFSY |
| G8CSB7 | NS5B protein | 2582,1642 | 17 - 37 | 86 | 0.00028 | RKNPMGFSYDTRCFDSTVTER** |
| G8CSB7 | NS5B protein | 1073,4488 | 18 - 26 | 86 | 0.00028 | KNPMGFSYD** |
| G8CSB7 | NS5B protein | 1232,4954 | 20 - 29 | 86 | 0.00028 | PMGFSYDTRC |
| G8CSB7 | NS5B protein | 975,4120 | 21 - 28 | 86 | 0.00028 | MGFSYDTR |
| G8CSB7 | NS5B protein | 1961,9443 | 33 - 48 | 86 | 0.00028 | TVTERDIRTEHDIYQS |
| G8CSB7 | NS5B protein | 1147,5258 | 40 - 48 | 86 | 0.00028 | RTEHDIYQS |
| G8CSB7 | NS5B protein | 1216,5506 | 47 - 56 | 86 | 0.00028 | QSCQLEPEAR |
| G8CSB7 | NS5B protein | 1802,9196 | 48 - 63 | 86 | 0.00028 | SCQLEPEARKAITSLT |
| G8CSB7 | NS5B protein | 1129,5550 | 49 - 57 | 86 | 0.00028 | CQLEPEARK |
| G8CSB7 | NS5B protein | 1501,7558 | 49 - 61 | 86 | 0.00028 | CQLEPEARKAITS |
| G8CSB7 | NS5B protein | 2093,0575 | 52 - 69 | 86 | 0.00028 | EPEARKAITSLTERLYCG |
| G8CSB7 | NS5B protein | 1958,9594 | 57 - 73 | 86 | 0.00028 | KAITSLTERLYCGGPMY |
| G8CSB7 | NS5B protein | 2290,0293 | 62 - 80 | 86 | 0.00028 | LTERLYCGGPMYNSRGQLC** |
| G8CSB7 | NS5B protein | 2675,2050 | 67 - 89 | 86 | 0.00028 | YCGGPMYNSRGQLCGIRRCRASG |
| G8CSB7 | NS5B protein | 1820,8886 | 69 - 84 | 86 | 0.00028 | GGPMYNSRGQLCGIRR |
| G8CSB7 | NS5B protein | 2230,1535 | 75 - 94 | 86 | 0.00028 | SRGQLCGIRRCRASGVLPTS |
| G8CSB7 | NS5B protein | 1114,6142 | 76 - 84 | 86 | 0.00028 | RGQLCGIRR |
| G8CSB7 | NS5B protein | 1516,8079 | 79 - 91 | 86 | 0.00028 | LCGIRRCRASGVL |
| G8CSB7 | NS5B protein | 1134,5499 | 80 - 88 | 86 | 0.00028 | CGIRRCRAS |
| G8CSB7 | NS5B protein | 1688,8563 | 80 - 94 | 86 | 0.00028 | CGIRRCRASGVLPTS |
| G8CSB7 | NS5B protein | 1031,5407 | 81 - 89 | 86 | 0.00028 | GIRRCRASG |
| G8CSB7 | NS5B protein | 2582,2880 | 81 - 103 | 86 | 0.00028 | GIRRCRASGVLPTSLGNTMTCYI |
| G8CSB7 | NS5B protein | 2583,2720 | 84 - 106 | 86 | 0.00028 | RCRASGVLPTSLGNTMTCYIKAQ.A |
| G8CSB7 | NS5B protein | 1046,5179 | 85 - 94 | 86 | 0.00028 | CRASGVLPTS |
| G8CSB7 | NS5B protein | 799,4552 | 86 - 93 | 86 | 0.00028 | RASGVLPT |
| G8CSB7 | NS5B protein | 1170,6357 | 86 - 97 | 86 | 0.00028 | RASGVLPTSLGN |
| G8CSB7 | NS5B protein | 2084,0394 | 86 - 104 | 86 | 0.00028 | RASGVLPTSLGNTMTCYIK** |
| G8CSB7 | NS5B protein | 856,4654 | 89 - 97 | 86 | 0.00028 | GVLPTSLGN |
| G8CSB7 | NS5B protein | 1952,9700 | 89 - 106 | 86 | 0.00028 | GVLPTSLGNTMTCYIKAQ |
| G8CSB7 | NS5B protein | 2214,0483 | 90 - 109 | 86 | 0.00028 | VLPTSLGNTMTCYIKAQAAC |
| G8CSB7 | NS5B protein | 2114,9799 | 91 - 109 | 86 | 0.00028 | LPTSLGNTMTCYIKAQAAC** |
| G8CSB7 | NS5B protein | 2157,9969 | 92 - 110 | 86 | 0.00028 | PTSLGNTMTCYIKAQAACR |
| G8CSB7 | NS5B protein | 2101,9707 | 94 - 112 | 86 | 0.00028 | SLGNTMTCYIKAQAACRAA |
| G8CSB7 | NS5B protein | 1540,7378 | 95 - 108 | 86 | 0.00028 | LGNTMTCYIKAQAA |
| G8CSB7 | NS5B protein | 2282,0970 | 103 - 123 | 86 | 0.00028 | IKAQAACRAAGLTNFDMLVCG** |
| G8CSB7 | NS5B protein | 1498,6432 | 111 - 124 | 86 | 0.00028 | AAGLTNFDMLVCGD** |
| G8CSB7 | NS5B protein | 2208,0443 | 111 - 131 | 86 | 0.00028 | AAGLTNFDMLVCGDDLVVVAE |
| G8CSB7 | NS5B protein | 2820,3198 | 112 - 138 | 86 | 0.00028 | AGLTNFDMLVCGDDLVVVAESVGVPED |
| G8CSB7 | NS5B protein | 2234,0600 | 116 - 136 | 86 | 0.00028 | NFDMLVCGDDLVVVAESVGVP |
| G8CSB7 | NS5B protein | 1988,9435 | 118 - 136 | 86 | 0.00028 | DMLVCGDDLVVVAESVGVP** |
| G8CSB7 | NS5B protein | 1473,7385 | 120 - 133 | 86 | 0.00028 | LVCGDDLVVVAESV |
| G8CSB7 | NS5B protein | 1360,6545 | 121 - 133 | 86 | 0.00028 | VCGDDLVVVAESV |
| G8CSB7 | NS5B protein | 1516,7444 | 121 - 135 | 86 | 0.00028 | VCGDDLVVVAESVGV |
| G8CSB7 | NS5B protein | 1514,7287 | 122 - 136 | 86 | 0.00028 | CGDDLVVVAESVGVP |
| G8CSB7 | NS5B protein | 2124,0699 | 123 - 143 | 86 | 0.00028 | GDDLVVVAESVGVPEDAANLR |
| G8CSB7 | NS5B protein | 1142,5455 | 129 - 140 | 86 | 0.00028 | VAESVGVPEDAA |
| G8CSB7 | NS5B protein | 2106,9892 | 131 - 150 | 86 | 0.00028 | ESVGVPEDAANLRAFTEAMT |
| G8CSB7 | NS5B protein | 1226,6255 | 132 - 143 | 86 | 0.00028 | SVGVPEDAANLR |
| G8CSB7 | NS5B protein | 1789,8669 | 133 - 149 | 86 | 0.00028 | VGVPEDAANLRAFTEAM |
| G8CSB7 | NS5B protein | 2210,0790 | 133 - 152 | 86 | 0.00028 | VGVPEDAANLRAFTEAMTRY |
| G8CSB7 | NS5B protein | 1040,5251 | 134 - 143 | 86 | 0.00028 | GVPEDAANLR |
| G8CSB7 | NS5B protein | 827,4025 | 135 - 142 | 86 | 0.00028 | VPEDAANL |
| G8CSB7 | NS5B protein | 2325,1059 | 135 - 155 | 86 | 0.00028 | VPEDAANLRAFTEAMTRYSAP** |
| G8CSB7 | NS5B protein | 876,4453 | 138 - 145 | 86 | 0.00028 | DAANLRAF |
| G8CSB7 | NS5B protein | 2082,9793 | 140 - 158 | 86 | 0.00028 | ANLRAFTEAMTRYSAPPGD** |
| G8CSB7 | NS5B protein | 1515,7140 | 143 - 155 | 86 | 0.00028 | RAFTEAMTRYSAP** |
| G8CSB7 | NS5B protein | 1518,6773 | 148 - 161 | 86 | 0.00028 | AMTRYSAPPGDEPQ |
| G8CSB7 | NS5B protein | 1600,7290 | 163 - 176 | 86 | 0.00028 | TYDLELITSCSSNV |
| G8CSB7 | NS5B protein | 1423,6501 | 165 - 177 | 86 | 0.00028 | DLELITSCSSNVS |
| G8CSB7 | NS5B protein | 1832,8211 | 167 - 184 | 86 | 0.00028 | ELITSCSSNVSVAHDGTG |
| G8CSB7 | NS5B protein | 1232,5092 | 172 - 183 | 86 | 0.00028 | CSSNVSVAHDGT |
| G8CSB7 | NS5B protein | 2499,1779 | 174 - 195 | 86 | 0.00028 | SNVSVAHDGTGQRYYYLTRDPT |
| G8CSB7 | NS5B protein | 2214,0454 | 175 - 193 | 86 | 0.00028 | NVSVAHDGTGQRYYYLTRD |
| G8CSB7 | NS5B protein | 1614,7427 | 176 - 189 | 86 | 0.00028 | VSVAHDGTGQRYYY |
| G8CSB7 | NS5B protein | 2565,2612 | 176 - 198 | 86 | 0.00028 | VSVAHDGTGQRYYYLTRDPTGPL |
| G8CSB7 | NS5B protein | 1457,7191 | 187 - 198 | 86 | 0.00028 | YYYLTRDPTGPL |
| G8CSB7 | NS5B protein | 2142,0534 | 187 - 204 | 86 | 0.00028 | YYYLTRDPTGPLARAAWE |
| G8CSB7 | NS5B protein | 714,3701 | 188 - 192 | 86 | 0.00028 | YYLTR |
| G8CSB7 | NS5B protein | 1978,9901 | 188 - 204 | 86 | 0.00028 | YYLTRDPTGPLARAAWE |
| Q81598 | NS5B protein | 1939,7928 | 9 - 23 | 65 | 0.07 | EEEIYQCCDLEPEAR |
| Q81598 | NS5B protein | 912,5028 | 19 - 26 | 65 | 0.07 | EPEARKAI |
| Q81598 | NS5B protein | 1183,6560 | 19 - 29 | 65 | 0.07 | EPEARKAISAL |
| Q81598 | NS5B protein | 1790,0050 | 21 - 36 | 65 | 0.07 | EARKAISALTERLYIG |
| Q81598 | NS5B protein | 1376,8027 | 24 - 35 | 65 | 0.07 | KAISALTERLYI |
| Q81598 | NS5B protein | 1998,0244 | 28 - 45 | 65 | 0.07 | ALTERLYIGGPMYNSKGL** |
| Q81598 | NS5B protein | 1756,8818 | 29 - 43 | 65 | 0.07 | LTERLYIGGPMYNSK** |
| Q81598 | NS5B protein | 1213,5801 | 31 - 40 | 65 | 0.07 | ERLYIGGPMY |
| Q81598 | NS5B protein | 2430,1467 | 35 - 55 | 65 | 0.07 | IGGPMYNSKGLQCGYRRCRAS |
| Q81598 | NS5B protein | 2503,1631 | 35 - 56 | 65 | 0.07 | IGGPMYNSKGLQCGYRRCRASG |
| Q81598 | NS5B protein | 1879,9621 | 43 - 58 | 65 | 0.07 | KGLQCGYRRCRASGVL |
| Q81598 | NS5B protein | 1977,0149 | 43 - 59 | 65 | 0.07 | KGLQCGYRRCRASGVLP |
| Q81598 | NS5B protein | 1096,5587 | 66 - 75 | 65 | 0.07 | VTCYIKATAA |
| Q81598 | NS5B protein | 1833,9407 | 72 - 89 | 65 | 0.07 | ATAASRAAGLKDPSFLVC |
| Q81598 | NS5B protein | 843,4926 | 74 - 82 | 65 | 0.07 | AASRAAGLK |
| Q81598 | NS5B protein | 1402,7568 | 74 - 87 | 65 | 0.07 | AASRAAGLKDPSFL |
| Q81598 | NS5B protein | 1501,8253 | 74 - 88 | 65 | 0.07 | AASRAAGLKDPSFLV |
| Q81598 | NS5B protein | 1147,5986 | 76 - 86 | 65 | 0.07 | SRAAGLKDPSF |
| Q81598 | NS5B protein | 1272,7190 | 77 - 88 | 65 | 0.07 | RAAGLKDPSFLV |
| Q81598 | NS5B protein | 2379,0570 | 88 - 108 | 65 | 0.07 | VCEDDLVVISESCGVEEDRVA |
| Q81598 | NS5B protein | 2492,1411 | 88 - 109 | 65 | 0.07 | VCEDDLVVISESCGVEEDRVAL |
| Q81598 | NS5B protein | 2030,0466 | 93 - 110 | 65 | 0.07 | LVVISESCGVEEDRVALR |
| Q81598 | NS5B protein | 2644,2373 | 100 - 122 | 65 | 0.07 | CGVEEDRVALRAFTEAMTRYSAP**) |
| Q81598 | NS5B protein | 1061,5982 | 106 - 114 | 65 | 0.07 | RVALRAFTE |
| Q81598 | NS5B protein | 2290,1117 | 118 - 138 | 65 | 0.07 | RYSAPPGDAPQPTYDLELITS |
| Q81598 | NS5B protein | 714,3701 | 155 - 159 | 65 | 0.07 | YYLTR |
| Q81598 | NS5B protein | 1547,8096 | 156 - 168 | 65 | 0.07 | YLTRDPEIPFARA |
| Q81598 | NS5B protein | 1110,5418 | 171 - 180 | 65 | 0.07 | ETARHTPVNS |
| Q81598 | NS5B protein | 877,4731 | 182 - 189 | 65 | 0.07 | LGNIIMFA |
| Q81598 | NS5B protein | 2124,0723 | 185 - 201 | 65 | 0.07 | IIMFAPTIWVRMVFMTH** |
| Q81598 | NS5B protein | 3685,8863 | 185 - 214 | 65 | 0.07 | IIMFAPTIWVRMVFMTHFFSILQAQEQLEK** |
| Q81598 | NS5B protein | 841,4810 | 189 - 195 | 65 | 0.07 | APTIWVR |
| Q81598 | NS5B protein | 1435,7105 | 191 - 201 | 65 | 0.07 | TIWVRMVFMTH** |
| Q81598 | NS5B protein | 1230,5678 | 195 - 203 | 65 | 0.07 | RMVFMTHFF |
| Q81598 | NS5B protein | 2214,0489 | 196 - 213 | 65 | 0.07 | MVFMTHFFSILQAQEQLE** |
| Q81598 | NS5B protein | 2318,1140 | 203 - 221 | 65 | 0.07 | FSILQAQEQLEKALDFEMY** |
| Q81598 | NS5B protein | 787,4076 | 204 - 210 | 65 | 0.07 | SILQAQE |
| Q81598 | NS5B protein | 844,4290 | 209 - 215 | 65 | 0.07 | QEQLEKA |
| Q81598 | NS5B protein | 829,4545 | 210 - 216 | 65 | 0.07 | EQLEKAL |
| Q81598 | NS5B protein | 1471,7419 | 246 - 258 | 65 | 0.07 | SLHGYSPTELNRV |
| Q81598 | NS5B protein | 1226,6619 | 251 - 262 | 65 | 0.07 | SPTELNRVAGAL |
| Q81598 | NS5B protein | 1423,8259 | 252 - 264 | 65 | 0.07 | PTELNRVAGALRK |
| Q81598 | NS5B protein | 855,5039 | 256 - 263 | 65 | 0.07 | NRVAGALR |
| Q81598 | NS5B protein | 1064,6243 | 266 - 274 | 65 | 0.07 | GIPPLRAWR |
| Q81598 | NS5B protein | 1031,5777 | 268 - 275 | 65 | 0.07 | PPLRAWRH |
| Q81598 | NS5B protein | 1969,0608 | 294 - 309 | 65 | 0.07 | CGLYLFNWAVRTKTKL |
| Q81598 | NS5B protein | 1068,6543 | 305 - 314 | 65 | 0.07 | TKTKLTPLPA |
| Q81598 | NS5B protein | 2015,0364 | 310 - 329 | 65 | 0.07 | TPLPAAGQLDLSSWFTVGVG |
| Q81598 | NS5B protein | 764,3858 | 323 - 329 | 65 | 0.07 | WFTVGVG |
| Q81598 | NS5B protein | 2191,1247 | 324 - 343 | 65 | 0.07 | FTVGVGGNDIYHSVSRARTR |
| Q81598 | NS5B protein | 2210,1491 | 331 - 348 | 65 | 0.07 | NDIYHSVSRARTRHLLLC |
| Q81598 | NS5B protein | 975,4774 | 332 - 339 | 65 | 0.07 | DIYHSVSR |
| Q81598 | NS5B protein | 1466,9476 | 349 - 362 | 65 | 0.07 | LLLLTVGVGIFLLP |
| C7SCB7 | NS5B protein | 1402,6464 | 2 - 15 | 86 | 0.00031 | GDDLVVVAESDGVE |
| C7SCB7 | NS5B protein | 1800,8741 | 4 - 20 | 86 | 0.00031 | DLVVVAESDGVEEDRAV |
| C7SCB7 | NS5B protein | 1244,6136 | 5 - 16 | 86 | 0.00031 | LVVVAESDGVEE |
| C7SCB7 | NS5B protein | 3112,5499 | 5 - 32 | 86 | 0.00031 | LVVVAESDGVEEDRAVLRAFTEAMTRYS |
| C7SCB7 | NS5B protein | 1762,8486 | 9 - 24 | 86 | 0.00031 | AESDGVEEDRAVLRAF |
| C7SCB7 | NS5B protein | 2111,9793 | 11 - 29 | 86 | 0.00031 | SDGVEEDRAVLRAFTEAMT** |
| C7SCB7 | NS5B protein | 2050,0266 | 13 - 30 | 86 | 0.00031 | D.GVEEDRAVLRAFTEAMTR.Y |
| C7SCB7 | NS5B protein | 717,3293 | 14 - 19 | 86 | 0.00031 | VEEDRA |
| C7SCB7 | NS5B protein | 2765,3079 | 15 - 39 | 86 | 0.00031 | EEDRAVLRAFTEAMTRYSAPPGDAP** |
| C7SCB7 | NS5B protein | 1047,5825 | 17 - 25 | 86 | 0.00031 | DRAVLRAFT |
| C7SCB7 | NS5B protein | 1107,5746 | 19 - 28 | 86 | 0.00031 | AVLRAFTEAM |
| C7SCB7 | NS5B protein | 1952,9778 | 19 - 36 | 86 | 0.00031 | AVLRAFTEAMTRYSAPPG** |
| C7SCB7 | NS5B protein | 1614,8188 | 20 - 33 | 86 | 0.00031 | VLRAFTEAMTRYSA |
| C7SCB7 | NS5B protein | 2609,2319 | 27 - 50 | 86 | 0.00031 | AMTRYSAPPGDAPQPTYDLELITS** |
| C7SCB7 | NS5B protein | 1847,8690 | 29 - 45 | 86 | 0.00031 | TRYSAPPGDAPQPTYDL |
| C7SCB7 | NS5B protein | 1098,4982 | 31 - 41 | 86 | 0.00031 | YSAPPGDAPQP |
| C7SCB7 | NS5B protein | 2451,1588 | 39 - 60 | 86 | 0.00031 | PQPTYDLELITSCSSNVSVARD |
| C7SCB7 | NS5B protein | 864,3865 | 40 - 46 | 86 | 0.00031 | QPTYDLE |
| C7SCB7 | NS5B protein | 764,3956 | 43 - 48 | 86 | 0.00031 | YDLELI |
| C7SCB7 | NS5B protein | 1423,6501 | 44 - 56 | 86 | 0.00031 | DLELITSCSSNVS |
| C7SCB7 | NS5B protein | 1774,9114 | 57 - 70 | 86 | 0.00031 | VARDDKGRRYYYLT |
| C7SCB7 | NS5B protein | 2117,0766 | 57 - 73 | 86 | 0.00031 | VARDDKGRRYYYLTRDA |
| C7SCB7 | NS5B protein | 1064,5475 | 59 - 66 | 86 | 0.00031 | RDDKGRRY |
| C7SCB7 | NS5B protein | 2214,1657 | 63 - 81 | 86 | 0.00031 | K.GRRYYYLTRDATTPLARAA.W |
| C7SCB7 | NS5B protein | 1475,7296 | 66 - 77 | 86 | 0.00031 | YYYLTRDATTPL |
| C7SCB7 | NS5B protein | 714,3701 | 67 - 71 | 86 | 0.00031 | YYLTR |
| C7SCB7 | NS5B protein | 2120,0875 | 71 - 89 | 86 | 0.00031 | RDATTPLARAAWETARHTP |
| C7SCB7 | NS5B protein | 843,4450 | 72 - 79 | 86 | 0.00031 | DATTPLAR |
| C7SCB7 | NS5B protein | 1185,6142 | 73 - 83 | 86 | 0.00031 | ATTPLARAAWE |
| C7SCB7 | NS5B protein | 1650,8590 | 73 - 87 | 86 | 0.00031 | ATTPLARAAWETARH |
| C7SCB7 | NS5B protein | 1377,7265 | 76 - 87 | 86 | 0.00031 | PLARAAWETARH |
| C7SCB7 | NS5B protein | 1575,8270 | 76 - 89 | 86 | 0.00031 | PLARAAWETARHTP |
| C7SCB7 | NS5B protein | 2247,1508 | 76 - 96 | 86 | 0.00031 | PLARAAWETARHTPVNSSLGN |
| C7SCB7 | NS5B protein | 1665,8335 | 78 - 92 | 86 | 0.00031 | ARAAWETARHTPVNS |
| C7SCB7 | NS5B protein | 1030,5308 | 78 - 86 | 86 | 0.00031 | ARAAWETAR |
| C7SCB7 | NS5B protein | 1268,6374 | 78 - 88 | 86 | 0.00031 | ARAAWETARHT |
| C7SCB7 | NS5B protein | 2036,0439 | 80 - 98 | 86 | 0.00031 | AAWETARHTPVNSSLGNII |
| C7SCB7 | NS5B protein | 2112,0422 | 81 - 99 | 86 | 0.00031 | AWETARHTPVNSSLGNIIM** |
| C7SCB7 | NS5B protein | 1553,7984 | 86 - 99 | 86 | 0.00031 | RHTPVNSSLGNIIM** |
| C7SCB7 | NS5B protein | 1544,7657 | 87 - 100 | 86 | 0.00031 | HTPVNSSLGNIIMY |
| C7SCB7 | NS5B protein | 1789,9284 | 88 - 104 | 86 | 0.00031 | TPVNSSLGNIIMYAPTI |
| C7SCB7 | NS5B protein | 1490,7439 | 89 - 102 | 86 | 0.00031 | PVNSSLGNIIMYAP** |
| C7SCB7 | NS5B protein | 2658,3042 | 90 - 112 | 86 | 0.00031 | VNSSLGNIIMYAPTIWVRMVMMT** |
| C7SCB7 | NS5B protein | 2311,1527 | 91 - 110 | 86 | 0.00031 | NSSLGNIIMYAPTIWVRMVM** |
| C7SCB7 | NS5B protein | 849,4266 | 92 - 99 | 86 | 0.00031 | SSLGNIIM** |
| C7SCB7 | NS5B protein | 2197,1098 | 92 - 110 | 86 | 0.00031 | SSLGNIIMYAPTIWVRMVM** |
| C7SCB7 | NS5B protein | 1879,9688 | 93 - 108 | 86 | 0.00031 | SLGNIIMYAPTIWVRM** |
| C7SCB7 | NS5B protein | 1091,5685 | 94 - 103 | 86 | 0.00031 | LGNIIMYAPT |
| C7SCB7 | NS5B protein | 1277,6478 | 95 - 105 | 86 | 0.00031 | GNIIMYAPTIW |
| C7SCB7 | NS5B protein | 1548,8123 | 95 - 107 | 86 | 0.00031 | GNIIMYAPTIWVR** |
| C7SCB7 | NS5B protein | 1970,9854 | 97 - 112 | 86 | 0.00031 | IIMYAPTIWVRMVMMT** |
| C7SCB7 | NS5B protein | 1108,5627 | 98 - 106 | 86 | 0.00031 | IMYAPTIWV** |
| C7SCB7 | NS5B protein | 1248,6689 | 98 - 107 | 86 | 0.00031 | IMYAPTIWVR |
| C7SCB7 | NS5B protein | 2142,0287 | 98 - 114 | 86 | 0.00031 | IMYAPTIWVRMVMMTHF** |
| C7SCB7 | NS5B protein | 2945,4464 | 98 - 121 | 86 | 0.00031 | IMYAPTIWVRMVMMTHFFSILQSQ** |
| C7SCB7 | NS5B protein | 848,4432 | 100 - 106 | 86 | 0.00031 | YAPTIWV |
| C7SCB7 | NS5B protein | 841,4810 | 101 - 107 | 86 | 0.00031 | APTIWVR |
| C7SCB7 | NS5B protein | 1147,5882 | 102 - 110 | 86 | 0.00031 | PTIWVRMVM** |
| C7SCB7 | NS5B protein | 1826,8671 | 102 - 115 | 86 | 0.00031 | PTIWVRMVMMTHFF** |
| C7SCB7 | NS5B protein | 2188,0268 | 105 - 121 | 86 | 0.00031 | WVRMVMMTHFFSILQSQ** |
| C7SCB7 | NS5B protein | 1545,7142 | 106 - 117 | 86 | 0.00031 | VRMVMMTHFFSI** |
| C7SCB7 | NS5B protein | 1414,6560 | 107 - 117 | 86 | 0.00031 | RMVMMTHFFSI** |
| C7SCB7 | NS5B protein | 1870,8892 | 107 - 121 | 86 | 0.00031 | RMVMMTHFFSILQSQ** |
| C7SCB7 | NS5B protein | 1698,7932 | 108 - 121 | 86 | 0.00031 | MVMMTHFFSILQSQ |
| C7SCB7 | NS5B protein | 1143,5093 | 109 - 117 | 86 | 0.00031 | VMMTHFFSI** |
| C7SCB7 | NS5B protein | 1206,6033 | 112 - 121 | 86 | 0.00031 | THFFSILQSQ |
| C7SCB7 | NS5B protein | 1731,8944 | 113 - 126 | 86 | 0.00031 | HFFSILQSQEILDR |
| C7SCB7 | NS5B protein | 1594,8355 | 114 - 126 | 86 | 0.00031 | FFSILQSQEILDR |
| C7SCB7 | NS5B protein | 1804,9723 | 114 - 128 | 86 | 0.00031 | FFSILQSQEILDRPL |
| C7SCB7 | NS5B protein | 2788,3266 | 119 - 142 | 86 | 0.00031 | QSQEILDRPLDFEMYGATYSVTPL** |
| C7SCB7 | NS5B protein | 1724,8080 | 121 - 134 | 86 | 0.00031 | QEILDRPLDFEMYG |
| C7SCB7 | NS5B protein | 1820,8179 | 128 - 143 | 86 | 0.00031 | LDFEMYGATYSVTPLD |
| E7BK75 | NS5B protein (3b) | 2214,1579 | 1 - 20 | 69 | 0.016 | EARKAISSLTERLYVGGPMH |
| E7BK75 | NS5B protein (3b) | 1272,7513 | 3 - 13 | 69 | 0.016 | RKAISSLTERL |
| E7BK75 | NS5B protein (3b) | 2030,0731 | 3 - 20 | 69 | 0.016 | RKAISSLTERLYVGGPMH** |
| E7BK75 | NS5B protein (3b) | 2015,0258 | 9 - 26 | 69 | 0.016 | LTERLYVGGPMHNSKGLQ** |
| E7BK75 | NS5B protein (3b) | 1976,8880 | 16 - 32 | 69 | 0.016 | GGPMHNSKGLQCGYRRC |
| E7BK75 | NS5B protein (3b) | 2503,2107 | 17 - 38 | 69 | 0.016 | GPMHNSKGLQCGYRRCRASGVL |
| E7BK75 | NS5B protein (3b) | 2644,2897 | 18 - 40 | 69 | 0.016 | PMHNSKGLQCGYRRCRASGVLPT |
| E7BK75 | NS5B protein (3b) | 3685,8596 | 37 - 71 | 69 | 0.016 | VLPTSFGNTVTCYIKATAASRAAGLKNPSFLVCGD |
| E7BK75 | NS5B protein (3b) | 1096,5587 | 46 - 55 | 69 | 0.016 | VTCYIKATAA |
| E7BK75 | NS5B protein (3b) | 1226,6731 | 52 - 64 | 69 | 0.016 | ATAASRAAGLKNP |
| E7BK75 | NS5B protein (3b) | 843,4926 | 54 - 62 | 69 | 0.016 | AASRAAGLK |
| E7BK75 | NS5B protein (3b) | 912,5141 | 57 - 65 | 69 | 0.016 | RAAGLKNPS |
| E7BK75 | NS5B protein (3b) | 1271,7350 | 57 - 68 | 69 | 0.016 | RAAGLKNPSFLV |
| E7BK75 | NS5B protein (3b) | 1115,6339 | 58 - 68 | 69 | 0.016 | AAGLKNPSFLV |
| E7BK75 | NS5B protein (3b) | 916,5382 | 61 - 68 | 69 | 0.016 | LKNPSFLV |
| E7BK75 | NS5B protein (3b) | 2210,0572 | 80 - 98 | 69 | 0.016 | CGVEEDRVALRAFTEAMTR |
| E7BK75 | NS5B protein (3b) | 1061,5982 | 86 - 94 | 69 | 0.016 | RVALRAFTE |
| E7BK75 | NS5B protein (3b) | 2290,0940 | 95 - 115 | 69 | 0.016 | AMTRYSAPPGDAPQPVYDLEL |
| E7BK75 | NS5B protein (3b) | 2379,0940 | 99 - 120 | 69 | 0.016 | YSAPPGDAPQPVYDLELITSCS |
| E7BK75 | NS5B protein (3b) | 975,4913 | 108 - 115 | 69 | 0.016 | QPVYDLEL |
| E7BK75 | NS5B protein (3b) | 764,3956 | 111 - 116 | 69 | 0.016 | YDLELI |
| E7BK75 | NS5B protein (3b) | 1756,8189 | 111 - 126 | 69 | 0.016 | YDLELITSCSSNVSVA |
| E7BK75 | NS5B protein (3b) | 2430,1638 | 124 - 144 | 69 | 0.016 | SVACDGAGKRYYYLTRDPEIP |
| E7BK75 | NS5B protein (3b) | 714,3701 | 135 - 139 | 69 | 0.016 | YYLTR |
| E7BK75 | NS5B protein (3b) | 1547,8096 | 136 - 148 | 69 | 0.016 | YLTRDPEIPFARA.A |
| E7BK75 | NS5B protein (3b) | 2124,0575 | 150 - 167 | 69 | 0.016 | WETARHTPVNSWLGNIIM |
| E7BK75 | NS5B protein (3b) | 1110,5418 | 151 - 160 | 69 | 0.016 | ETARHTPVNS |
| E7BK75 | NS5B protein (3b) | 2191,1686 | 161 - 178 | 69 | 0.016 | WLGNIIMFAPTIWVRMVL.M** |
| E7BK75 | NS5B protein (3b) | 877,4731 | 162 - 169 | 69 | 0.016 | LGNIIMFA |
| E7BK75 | NS5B protein (3b) | 1376,7162 | 163 - 174 | 69 | 0.016 | GNIIMFAPTIWV** |
| E7BK75 | NS5B protein (3b) | 1953,0290 | 165 - 180 | 69 | 0.016 | IIMFAPTIWVRMVLMT |
| E7BK75 | NS5B protein (3b) | 841,4810 | 169 - 175 | 69 | 0.016 | APTIWVR |
| E7BK75 | NS5B protein (3b) | 1244,6774 | 170 - 179 | 69 | 0.016 | PTIWVRMVLM |
| E7BK75 | NS5B protein (3b) | 1147,6246 | 171 - 179 | 69 | 0.016 | TIWVRMVLM |
| E7BK75 | NS5B protein (3b) | 1879,9841 | 171 - 185 | 69 | 0.016 | TIWVRMVLMTHFFSI.L |
| E7BK75 | NS5B protein (3b) | 1608,8520 | 174 - 186 | 69 | 0.016 | VRMVLMTHFFSIL** |
| E7BK75 | NS5B protein (3b) | 844,4290 | 189 - 195 | 69 | 0.016 | QEQLEKA |
| E7BK75 | NS5B protein (3b) | 829,4545 | 190 - 196 | 69 | 0.016 | EQLEKAL |
| E7BK75 | NS5B protein (3b) | 2318,0963 | 194 - 214 | 69 | 0.016 | KALNFEMYGATYSVTPMDLPA |
| E7BK75 | NS5B protein (3b) | 1435,7269 | 204 - 216 | 69 | 0.016 | TYSVTPMDLPAII** |
| E7BK75 | NS5B protein (3b) | 1833,9546 | 204 - 219 | 69 | 0.016 | TYSVTPMDLPAIIERL** |
| E7BK75 | NS5B protein (3b) | 1068,5889 | 207 - 216 | 69 | 0.016 | VTPMDLPAII |
| E7BK75 | NS5B protein (3b) | 1213,6264 | 207 - 217 | 69 | 0.016 | VTPMDLPAIIE** |
| E7BK75 | NS5B protein (3b) | 1789,9760 | 207 - 222 | 69 | 0.016 | VTPMDLPAIIERLHGL** |
| E7BK75 | NS5B protein (3b) | 1098,5631 | 208 - 217 | 69 | 0.016 | TPMDLPAIIE |
| E7BK75 | NS5B protein (3b) | 787,4150 | 210 - 216 | 69 | 0.016 | MDLPAII** |
| E7BK75 | NS5B protein (3b) | 1998,0608 | 210 - 227 | 69 | 0.016 | MDLPAIIERLHGLSAFSL** |
| E7BK75 | NS5B protein (3b) | 1226,6619 | 231 - 242 | 69 | 0.016 | SPTELNRVAGAL |
| E7BK75 | NS5B protein (3b) | 1423,8259 | 232 - 244 | 69 | 0.016 | PTELNRVAGALRK |
| E7BK75 | NS5B protein (3b) | 855,5039 | 236 - 243 | 69 | 0.016 | NRVAGALR |
| E7BK75 | NS5B protein (3b) | 1064,6243 | 246 - 254 | 69 | 0.016 | GIPPLRAWR |
| E7BK75 | NS5B protein (3b) | 1031,5777 | 248 - 255 | 69 | 0.016 | PPLRAWRH |
| E7BK75 | NS5B protein (3b) | 1604,9151 | 278 - 290 | 69 | 0.016 | LFNWAVRTKTRLT |

*Modification – Carbamidomethyl (C). ** Modification - Oxidation (M).

**Supplementary Table S3. Hare MS/MS peptide sequence report. MS/MS peptide sequences reported by Mascot version 2.4 from *de novo* inoculated fresh naïve MDBK cells using supernatant of first inoculated naïve MDBK cells with hare DNA sample, 7 days post inoculation. P.S. > 64 are significant (p<0.05).**

| **UniProt accession no.. (genotype)** | **HCV fragment (genotype)** | **Calculated mass (Da)** | **Genome position (amino-acid)** | **Protein score** | **Expect-value** | **Peptide sequence** |
| --- | --- | --- | --- | --- | --- | --- |
| **B3TL57** | **F protein (1a)** | 841.4770 | 7 - 13 | 74 | 0.0053 | PQRKTNV |
| **B3TL57** | **F protein (1a)** | 1575.8845 | 6 - 19 | 74 | 0.0053 | KPQRKTNVTPTVAH |
| **B3TL57** | **F protein (1a)** | 2478.2332 | 18 - 38 | 74 | 0.0053 | AHRTSSSRVAVRSLVEFTCCR |
| **B3TL57** | **F protein (1a)** | 829.4155 | 19 - 25 | 74 | 0.0053 | A.HRTSSSR.V |
| **B3TL57** | **F protein (1a)** | 1879.9509 | 25 - 40 | 74 | 0.0053 | RVAVRSLVEFTCCRAG |
| **B3TL57** | **F protein (1a)** | 850.4549 | 29 - 35 | 74 | 0.0053 | RSLVEFT |
| **B3TL57** | **F protein (1a)** | 761.3490 | 37 - 43 | 74 | 0.0053 | C.CRAGALD.W |
| **B3TL57** | **F protein (1a)** | 1083.6084 | 45 - 53 | 74 | 0.0053 | VCARRGRLP |
| **B3TL57** | **F protein (1a)** | 855.4675 | 50 - 57 | 74 | 0.0053 | GRLPSGRN |
| **B3TL57** | **F protein (1a)** | 1553.8525 | 51 - 64 | 74 | 0.0053 | RLPSGRNLEVDVSL |
| **B3TL57** | **F protein (1a)** | 1197.6353 | 52 - 62 | 74 | 0.0053 | LPSGRNLEVDV |
| **B3TL57** | **F protein (1a)** | 1724.8958 | 54 - 68 | 74 | 0.0053 | SGRNLEVDVSLFPRH |
| **B3TL57** | **F protein (1a)** | 2134.1396 | 54 - 72 | 74 | 0.0053 | SGRNLEVDVSLFPRHVGPR |
| **B3TL57** | **F protein (1a)** | 2229.2018 | 57 - 77 | 74 | 0.0053 | NLEVDVSLFPRHVGPRAGPGL |
| **B3TL57** | **F protein (1a)** | 832.4443 | 61 - 67 | 74 | 0.0053 | DVSLFPR |
| **B3TL57** | **F protein (1a)** | 1107.6189 | 62 - 71 | 74 | 0.0053 | VSLFPRHVGP |
| **B3TL57** | **F protein (1a)** | 848.4729 | 67 - 74 | 74 | 0.0053 | RHVGPRAG |
| **B3TL57** | **F protein (1a)** | 1971.0473 | 67 - 86 | 74 | 0.0053 | RHVGPRAGPGLSPGILGPSM** |
| **B3TL57** | **F protein (1a)** | 1277.7092 | 72 - 85 | 74 | 0.0053 | RAGPGLSPGILGPS |
| **B3TL57** | **F protein (1a)** | 2584.2598 | 72 - 98 | 74 | 0.0053 | RAGPGLSPGILGPSMATRAAGGRDGSC** |
| **B3TL57** | **F protein (1a)** | 1121.6081 | 73 - 85 | 74 | 0.0053 | AGPGLSPGILGPS |
| **B3TL57** | **F protein (1a)** | 1848.8571 | 80 - 98 | 74 | 0.0053 | GILGPSMATRAAGGRDGSC** |
| **B3TL57** | **F protein (1a)** | 831.4273 | 82 - 89 | 74 | 0.0053 | LGPSMATR |
| **B3TL57** | **F protein (1a)** | 1030.5229 | 82 - 92 | 74 | 0.0053 | LGPSMATRAAG |
| **B3TL57** | **F protein (1a)** | 1789.8563 | 86 - 103 | 74 | 0.0053 | MATRAAGGRDGSCLPAAL** |
| **B3TL57** | **F protein (1a)** | 2945.5294 | 90 - 120 | 74 | 0.0053 | AAGGRDGSCLPAALGLAGAPQTPGVGRAIWV |
| **B3TL57** | **F protein (1a)** | 2036.0109 | 91 - 112 | 74 | 0.0053 | AGGRDGSCLPAALGLAGAPQTP |
| **B3TL57** | **F protein (1a)** | 2249.1223 | 91 - 115 | 74 | 0.0053 | AGGRDGSCLPAALGLAGAPQTPGVG |
| **B3TL57** | **F protein (1a)** | 1143.5594 | 95 - 106 | 74 | 0.0053 | DGSCLPAALGLA |
| **B3TL57** | **F protein (1a)** | 3073.6131 | 95 - 125 | 74 | 0.0053 | DGSCLPAALGLAGAPQTPGVGRAIWVRSSIP |
| **B3TL57** | **F protein (1a)** | 844.4113 | 96 - 104 | 74 | 0.0053 | GSCLPAALG |
| **B3TL57** | **F protein (1a)** | 1602.8842 | 100 - 117 | 74 | 0.0053 | PAALGLAGAPQTPGVGRA |
| **B3TL57** | **F protein (1a)** | 1804.9948 | 101 - 119 | 74 | 0.0053 | AALGLAGAPQTPGVGRAIW |
| **B3TL57** | **F protein (1a)** | 1129.6356 | 114 - 123 | 74 | 0.0053 | VGRAIWVRSS |
| **B3TL57** | **F protein (1a)** | 1157.6305 | 119 - 128 | 74 | 0.0053 | WVRSSIPSRA |
| **B3TL57** | **F protein (1a)** | 1226.6731 | 120 - 131 | 74 | 0.0053 | VRSSIPSRAASP |
| **B3TL57** | **F protein (1a)** | 1402.6841 | 122 - 135 | 74 | 0.0053 | SSIPSRAASPTSWG |
| **B3TL57** | **F protein (1a)** | 1545.7576 | 133 - 147 | 74 | 0.0053 | SWGTYRSSAPPLGAP |
| **B3TL57** | **F protein (1a)** | 1268.6513 | 137 - 149 | 74 | 0.0053 | YRSSAPPLGAPPG |
| **B3TL57** | **F protein (1a)** | 1762.8937 | 138 - 154 | 74 | 0.0053 | RSSAPPLGAPPGPWRMA** |
| **B3TL57** | **F protein (1a)** | 1535.7555 | 139 - 153 | 74 | 0.0053 | SSAPPLGAPPGPWRM** |
| **B3TL57** | **F protein (1a)** | 2210.1095 | 141 - 161 | 74 | 0.0053 | APPLGAPPGPWRMASGFWKTA** |
| **B3TL57** | **F protein (1a)** | 1248.6437 | 143 - 154 | 74 | 0.0053 | PLGAPPGPWRMA |
| **B3TL57** | **F protein (1a)** | 1756.8872 | 144 - 159 | 74 | 0.0053 | LGAPPGPWRMASGFWK |
| **C0SUM8** | Genome polyprotein - Core protein (1b) | 2367.2884 | 11 - 31 | 77 | 0.0027 | TKRNTNRRPQDVKFPGGGQIV |
| **C0SUM8** | Genome polyprotein - Core protein (1b) | 1040.5587 | 13 - 20 | 77 | 0.0027 | RNTNRRPQ |
| **C0SUM8** | Genome polyprotein - Core protein (1b) | 1098.5530 | 14 - 22 | 77 | 0.0027 | NTNRRPQDV |
| **C0SUM8** | Genome polyprotein - Core protein (1b) | 1226.6480 | 14 - 23 | 77 | 0.0027 | NTNRRPQDVK |
| **C0SUM8** | Genome polyprotein - Core protein (1b) | 1114.6135 | 23 - 34 | 77 | 0.0027 | KFPGGGQIVGGV |
| **C0SUM8** | Genome polyprotein - Core protein (1b) | 2582.3295 | 66 - 87 | 77 | 0.0027 | PKARRPEGRTWAQPGYPWPLYG |
| **C0SUM8** | Genome polyprotein - Core protein (1b) | 2599.3196 | 67 - 88 | 77 | 0.0027 | KARRPEGRTWAQPGYPWPLYGN |
| **C0SUM8** | Genome polyprotein - Core protein (1b) | 1952.9758 | 69 - 84 | 77 | 0.0027 | RRPEGRTWAQPGYPWP |
| **C0SUM8** | Genome polyprotein - Core protein (1b) | 2535.1470 | 76 - 97 | 77 | 0.0027 | WAQPGYPWPLYGNEGMGWAGWL |
| **C0SUM8** | Genome polyprotein - Core protein (1b) | 1832.8345 | 82 - 97 | 77 | 0.0027 | PWPLYGNEGMGWAGWL |
| **C0SUM8** | Genome polyprotein - Core protein (1b) | 1473.6711 | 87 - 100 | 77 | 0.0027 | GNEGMGWAGWLLSP |
| **C0SUM8** | Genome polyprotein - Core protein (1b) | 1118.5219 | 89 - 98 | 77 | 0.0027 | N.EGMGWAGWLL.S |
| **C0SUM8** | Genome polyprotein - Core protein (1b) | 876.3952 | 90 - 97 | 77 | 0.0027 | GMGWAGWL |
| **C0SUM8** | Genome polyprotein - Core protein (1b) | 2214.0793 | 91 - 110 | 77 | 0.0027 | MGWAGWLLSPRGSRPSWGPT** |
| **C0SUM8** | Genome polyprotein - Core protein (1b) | 2566.2652 | 91 - 113 | 77 | 0.0027 | MGWAGWLLSPRGSRPSWGPTDPR |
| **C0SUM8** | Genome polyprotein - Core protein (1b) | 844.4304 | 101 - 107 | 77 | 0.0027 | RGSRPSW |
| **C0SUM8** | Genome polyprotein - Core protein (1b) | 827.3926 | 107 - 113 | 77 | 0.0027 | WGPTDPR |
| **C0SUM8** | Genome polyprotein - Core protein (1b) | 2133.0922 | 116 - 135 | 77 | 0.0027 | SRNLGKVIDTLTCGLADLMG |
| **C0SUM8** | Genome polyprotein - Core protein (1b) | 1541.7106 | 123 - 136 | 77 | 0.0027 | IDTLTCGLADLMGY |
| **C0SUM8** | Genome polyprotein - Core protein (1b) | 2569.3760 | 126 - 151 | 77 | 0.0027 | T.LTCGLADLMGYIPLVGAPLGGAARAL.A |
| **C0SUM8** | Genome polyprotein - Core protein (1b) | 1537.7520 | 127 - 140 | 77 | 0.0027 | TCGLADLMGYIPLV** |
| **C0SUM8** | Genome polyprotein - Core protein (1b) | 1870.9863 | 129 - 148 | 77 | 0.0027 | GLADLMGYIPLVGAPLGGAA** |
| **C0SUM8** | Genome polyprotein - Core protein (1b) | 1120.5838 | 130 - 139 | 77 | 0.0027 | LADLMGYIPL** |
| **C0SUM8** | Genome polyprotein - Core protein (1b) | 1129.6205 | 133 - 143 | 77 | 0.0027 | LMGYIPLVGAP |
| **C0SUM8** | Genome polyprotein - Core protein (1b) | 1242.7046 | 133 - 144 | 77 | 0.0027 | LMGYIPLVGAPL |
| **C0SUM8** | Genome polyprotein - Core protein (1b) | 1129.6205 | 134 - 144 | 77 | 0.0027 | MGYIPLVGAPL |
| **C0SUM8** | Genome polyprotein - Core protein (1b) | 1540.7998 | 151 - 164 | 77 | 0.0027 | LAHGVRVLEDGVNY |
| **C0SUM8** | Genome polyprotein - Core protein (1b) | 2214.0488 | 153 - 173 | 77 | 0.0027 | HGVRVLEDGVNYATGNLPGCS |
| **C0SUM8** | Genome polyprotein - Core protein (1b) | 879.4160 | 169 - 176 | 77 | 0.0027 | LPGCSFSI |
| **C0SUM8** | Genome polyprotein - Core protein (1b) | 1170.6682 | 179 - 189 | 77 | 0.0027 | LALLSCLTIPA |
| **C0SUM8** | Genome polyprotein - Core/E1 proteins (1b) | 2129.9609 | 181 - 200 | 77 | 0.0027 | LLSCLTIPASAFTSDCSNSS |
| B6USQ0 | E1 protein | 2409.0035 | 11 - 31 | 70 | 0.011 | HVTNDCSNSSIVYEAADMIMH** |
| B6USQ0 | E1 protein | 732.3402 | 39 - 44 | 70 | 0.011 | VREENS |
| B6USQ0 | E1 protein | 748.3578 | 47 - 52 | 70 | 0.011 | CWVALT |
| B6USQ0 | E1 protein | 1471.7606 | 47 - 59 | 70 | 0.011 | R.CWVALTPTLAARN.S |
| B6USQ0 | E1 protein | 855.5066 | 49 - 57 | 70 | 0.011 | VALTPTLAA |
| B6USQ0 | E1 protein | 1498.8328 | 56 - 69 | 70 | 0.011 | AARNSSVPTTAIRR |
| B6USQ0 | E1 protein | 1115.5935 | 57 - 67 | 70 | 0.011 | ARNSSVPTTAI |
| B6USQ0 | E1 protein | 1564.8546 | 57 - 70 | 70 | 0.011 | ARNSSVPTTAIRRH |
| B6USQ0 | E1 protein | 843.4814 | 61 - 68 | 70 | 0.011 | SVPTTAIR |
| B6USQ0 | E1 protein | 1148.6778 | 62 - 71 | 70 | 0.011 | VPTTAIRRHV |
| B6USQ0 | E1 protein | 2191.1895 | 63 - 83 | 70 | 0.011 | PTTAIRRHVDLLVGAAALCSA |
| B6USQ0 | E1 protein | 2124.1296 | 65 - 84 | 70 | 0.011 | TAIRRHVDLLVGAAALCSAM |
| B6USQ0 | E1 protein | 2214.1765 | 67 - 86 | 70 | 0.011 | IRRHVDLLVGAAALCSAMYV |
| B6USQ0 | E1 protein | 841.4909 | 72 - 80 | 70 | 0.011 | DLLVGAAAL |
| B6USQ0 | E1 protein | 1044.5638 | 73 - 83 | 70 | 0.011 | LLVGAAALCSA |
| B6USQ0 | E1 protein | 860.4426 | 74 - 82 | 70 | 0.011 | LVGAAALCS |
| B6USQ0 | E1 protein | 2503.2161 | 79 - 101 | 70 | 0.011 | ALCSAMYVGDLCGSVLLVSQLFT |
| B6USQ0 | E1 protein | 767.3524 | 83 - 89 | 70 | 0.011 | AMYVGDL |
| B6USQ0 | E1 protein | 1435.7745 | 89 - 101 | 70 | 0.011 | LCGSVLLVSQLFT |
| B6USQ0 | E1 protein | 2015.0952 | 92 - 108 | 70 | 0.011 | SVLLVSQLFTFSPRRHE |
| B6USQ0 | E1 protein | 1040.5543 | 95 - 103 | 70 | 0.011 | LVSQLFTFS |
| B6USQ0 | E1 protein | 794.4147 | 104 - 109 | 70 | 0.011 | PRRHET |
| B6USQ0 | E1 protein | 1353.6612 | 117 - 128 | 70 | 0.011 | IYPGHVTGHRMA** |
| B6USQ0 | E1 protein | 1956.8354 | 126 - 141 | 70 | 0.011 | RMAWDMMMNWSPTTAL** |
| B6USQ0 | E1 protein | 1789.9107 | 132 - 147 | 70 | 0.011 | MMNWSPTTALVVSQLL |
| B6USQ0 | E1 protein | 1634.9719 | 137 - 151 | 70 | 0.011 | PTTALVVSQLLRIPQ |
| B6USQ0 | E1 protein | 1535.9035 | 142 - 155 | 70 | 0.011 | VVSQLLRIPQAVVD |
| B6USQ0 | E1 protein | 1142.6118 | 148 - 157 | 70 | 0.011 | RIPQAVVDMV** |
| B6USQ0 | E1 protein | 1270.6704 | 148 - 159 | 70 | 0.011 | RIPQAVVDMVAG** |
| B6USQ0 | E1 protein | 1098.5743 | 149 - 159 | 70 | 0.011 | IPQAVVDMVAG |
| B6USQ0 | E1 protein | 975.4695 | 151 - 160 | 70 | 0.011 | QAVVDMVAGA** |
| B6USQ0 | E1 protein | 1879.9615 | 151 - 169 | 70 | 0.011 | QAVVDMVAGAHWGVLAGLA** |
| B6USQ0 | E1 protein | 1423.7282 | 153 - 166 | 70 | 0.011 | VVDMVAGAHWGVLA |
| B6USQ0 | E1 protein | 1978.9611 | 154 - 172 | 70 | 0.011 | VDMVAGAHWGVLAGLAYYS |
| B6USQ0 | E1 protein | 1514.7704 | 156 - 170 | 70 | 0.011 | MVAGAHWGVLAGLAY |
| B6USQ0 | E1 protein | 1213.6244 | 159 - 170 | 70 | 0.011 | GAHWGVLAGLAY |
| B6USQ0 | E1 protein | 1856.9495 | 164 - 180 | 70 | 0.011 | VLAGLAYYSMVGNWAKV** |
| B6USQ0 | E1 protein | 1834.0426 | 171 - 186 | 70 | 0.011 | YSMVGNWAKVLIVLLL** |
| B6USQ0 | E1 protein | 1454.8683 | 173 - 185 | 70 | 0.011 | MVGNWAKVLIVLL |
| B6USQ0 | E1 protein | 1628.9654 | 175 - 189 | 70 | 0.011 | GNWAKVLIVLLLFSG |
| E9LLA5 | Polyprotein - E2 protein | 1129.5808 | 1 - 10 | 65 | 0.084 | QTGFLAALFY |
| E9LLA5 | Polyprotein - E2 protein | 2111.8823 | 16 - 33 | 65 | 0.084 | SSGCPERMASCRPIDQFD |
| E9LLA5 | Polyprotein - E2 protein | 1414.6630 | 27 - 38 | 65 | 0.084 | RPIDQFDQGWGP |
| E9LLA5 | Polyprotein - E2 protein | 2141.9589 | 38 - 54 | 65 | 0.084 | G.PITYSERQGSDQRPYCW.H |
| E9LLA5 | Polyprotein - E2 protein | 2641.1768 | 39 - 59 | 65 | 0.084 | ITYSERQGSDQRPYCWHYAPQ |
| E9LLA5 | Polyprotein - E2 protein | 844.4113 | 61 - 68 | 65 | 0.084 | CGIIPASQ |
| E9LLA5 | Polyprotein - E2 protein | 2111.9479 | 61 - 79 | 65 | 0.084 | CGIIPASQVCGPVYCFTPS |
| E9LLA5 | Polyprotein - E2 protein | 1774.9254 | 76 - 92 | 65 | 0.084 | FTPSPVVVGTTDRFGVP |
| E9LLA5 | Polyprotein - E2 protein | 855.4702 | 77 - 85 | 65 | 0.084 | TPSPVVVGT |
| E9LLA5 | Polyprotein - E2 protein | 2233.1015 | 91 - 109 | 65 | 0.084 | VPTYRWGENETDVLLLNNT |
| E9LLA5 | Polyprotein - E2 protein | 1834.8849 | 94 - 108 | 65 | 0.084 | YRWGENETDVLLLNN |
| E9LLA5 | Polyprotein - E2 protein | 3073.4465 | 95 - 120 | 65 | 0.084 | RWGENETDVLLLNNTRPPQGNWFGCT |
| E9LLA5 | Polyprotein - E2 protein | 1143.5771 | 99 - 108 | 65 | 0.084 | NETDVLLLNN |
| E9LLA5 | Polyprotein - E2 protein | 1244.6248 | 99 - 109 | 65 | 0.084 | NETDVLLLNNT |
| E9LLA5 | Polyprotein - E2 protein | 2114.9184 | 133 - 152 | 65 | 0.084 | GPPCNIGGVGNNTLICPTDC |
| E9LLA5 | Polyprotein - E2 protein | 912.4818 | 166 - 173 | 65 | 0.084 | SGPWLTPR |
| E9LLA5 | Polyprotein - E2 protein | 831.4061 | 169 - 174 | 65 | 0.084 | WLTPRC |
| E9LLA5 | Polyprotein - E2 protein | 1030.4807 | 181 - 187 | 65 | 0.084 | RLWHYPC |
| E9LLA5 | Polyprotein - E2 protein | 736.3214 | 186 - 191 | 65 | 0.084 | PCTVNF |
| E9LLA5 | Polyprotein - E2 protein | 851.4687 | 195 - 201 | 65 | 0.084 | KVRMYVG |
| E9LLA5 | Polyprotein - E2 protein | 2188.0232 | 199 - 217 | 65 | 0.084 | YVGGVEHRLNAACNWTRGE |
| E9LLA5 | Polyprotein - E2 protein | 1121.5941 | 200 - 210 | 65 | 0.084 | VGGVEHRLNAA |
| E9LLA5 | Polyprotein - E2 protein | 1762.8104 | 206 - 219 | 65 | 0.084 | RLNAACNWTRGERC |
| E9LLA5 | Polyprotein - E2 protein | 761.3192 | 220 - 225 | 65 | 0.084 | DLEDRD |
| E9LLA5 | Polyprotein - E2 protein | 2478.3094 | 243 - 264 | 65 | 0.084 | PCFFTTLPALSTGLIHLHQNIV |
| E9LLA5 | Polyprotein - E2 protein | 1714.9366 | 247 - 262 | 65 | 0.084 | TTLPALSTGLIHLHQN |
| E9LLA5 | Polyprotein - E2 protein | 1157.5757 | 277 - 285 | 65 | 0.084 | VSYAIKWEY |
| E9LLA5 | Polyprotein - E2 protein | 1268.7420 | 284 - 293 | 65 | 0.084 | EYVLLLFLFL |
| E9LLA5 | Polyprotein - E2 protein | 2584.3442 | 287 - 307 | 65 | 0.084 | LLLFLFLADARVCACLWMMLL** |
| E9LLA5 | Polyprotein - E2 protein | 1540.6659 | 295 - 306 | 65 | 0.084 | DARVCACLWMML** |
| E9LLA5 | Polyprotein - E2/p7 proteins | 2945.4635 | 295 - 320 | 65 | 0.084 | DARVCACLWMMLLVAQAEAALENLVV |
| E9LLA5 | Polyprotein - E2/p7 proteins | 810.3227 | 300 - 305 | 65 | 0.084 | ACLWMM |
| E9LLA5 | Polyprotein - E2/p7 proteins | 1789.8929 | 300 - 315 | 65 | 0.084 | ACLWMMLLVAQAEAAL |
| E9LLA5 | Polyprotein - E2/p7 proteins | 1226.6506 | 308 - 319 | 65 | 0.084 | VAQAEAALENLV |
| E9LLA5 | Polyprotein - p7 protein | 869.5222 | 314 - 321 | 65 | 0.084 | ALENLVVL |
| E9LLA5 | Polyprotein - p7 protein | 1277.7092 | 319 - 332 | 65 | 0.084 | VVLNAASVAGAHGL |
| E9LLA5 | Polyprotein - p7 protein | 1083.6077 | 327 - 337 | 65 | 0.084 | AGAHGLLSFLV |
| E9LLA5 | Polyprotein - p7 protein | 1377.7445 | 327 - 339 | 65 | 0.084 | AGAHGLLSFLVFF |
| E9LLA5 | Polyprotein - p7 protein | 2945.6030 | 332 - 357 | 65 | 0.084 | LLSFLVFFCAAWYIKGRLVPGAAYAL |
| E9LLA5 | Polyprotein - p7 protein | 1602.8810 | 351 - 365 | 65 | 0.084 | PGAAYALYGVWPLLL |
| E9LLA5 | Polyprotein - NS2-3 protease | 1206.6067 | 382 - 394 | 65 | 0.084 | AASCGGAVFVGLV |
| E9LLA5 | Polyprotein - NS2-3 protease | 1774.9691 | 396 - 410 | 65 | 0.084 | LTLSPHYKEFLAMLI |
| E9LLA5 | Polyprotein - NS2-3 protease | 1565.8316 | 407 - 418 | 65 | 0.084 | AMLIWWLQYLIT** |
| E9LLA5 | Polyprotein - NS2-3 protease | 1516.7674 | 418 - 430 | 65 | 0.084 | TRAEAYLQVWSPP |
| E9LLA5 | Polyprotein - NS2-3 protease | 1971.0149 | 419 - 434 | 65 | 0.084 | RAEAYLQVWCPPLNVR |
| E9LLA5 | Polyprotein - NS2-3 protease | 1248.6543 | 422 - 431 | 65 | 0.084 | AYLQVWYPPL |
| E9LLA5 | Polyprotein - NS2-3 protease | 1107.6189 | 426 - 434 | 65 | 0.084 | VWQPPLNVR |
| E9LLA5 | Polyprotein - NS2-3 protease | 1731.9090 | 434 - 449 | 65 | 0.084 | RGGRDAAILLTCVAYP |
| E9LLA5 | Polyprotein - NS2-3 protease | 714.4024 | 436 - 442 | 65 | 0.084 | GRDAAIL |
| E9LLA5 | Polyprotein - NS2-3 protease | 1540.7483 | 444 - 456 | 65 | 0.084 | TCVAYPELIFDIT |
| E9LLA5 | Polyprotein - NS2-3 protease | 832.4728 | 454 - 460 | 65 | 0.084 | DITKMLL |
| E9LLA5 | Polyprotein - NS2-3 protease | 1804.9653 | 458 - 474 | 65 | 0.084 | MLLAIFGPLMVLQAGMT |
| E9LLA5 | Polyprotein - NS2-3 protease | 1575.8517 | 461 - 475 | 65 | 0.084 | AIFGPLMVLQAGMTK |
| E9LLA5 | Polyprotein - NS2-3 protease | 1879.9940 | 462 - 478 | 65 | 0.084 | IFGPLMVLQAGMTKVPY** |
| E9LLA5 | Polyprotein - NS2-3 protease | 1614.8150 | 467 - 480 | 65 | 0.084 | MVLQAGMTKVPYFV** |
| E9LLA5 | Polyprotein - NS2-3 protease | 2036.0336 | 467 - 484 | 65 | 0.084 | MVLQAGMTKVPYFVRAHG** |
| E9LLA5 | Polyprotein - NS2-3 protease | 1273.6931 | 474 - 484 | 65 | 0.084 | TKVPYFVRAHG |
| E9LLA5 | Polyprotein - NS2-3 protease | 2428.3712 | 476 - 495 | 65 | 0.084 | VPYFVRAHGVIRLCMLVRKV** |
| E9LLA5 | Polyprotein - NS2-3 protease | 1044.5505 | 477 - 485 | 65 | 0.084 | PYFVRAHGV |
| E9LLA5 | Polyprotein - NS2-3 protease | 1848.9855 | 478 - 492 | 65 | 0.084 | YFVRAHGVIRLCMLV** |
| E9LLA5 | Polyprotein - NS2-3 protease | 2229.2061 | 487 - 505 | 65 | 0.084 | RLCMLVRKVAGGHYVQMVL |
| E9LLA5 | Polyprotein - NS2-3 protease | 790.4193 | 488 - 493 | 65 | 0.084 | LCMLVR |
| E9LLA5 | Polyprotein - NS2-3 protease | 1197.6982 | 491 - 501 | 65 | 0.084 | LVRKVAGGHYV |
| E9LLA5 | Polyprotein - NS2-3 protease | 829.4446 | 494 - 501 | 65 | 0.084 | KVAGGHYV |
| E9LLA5 | Polyprotein - NS2-3 protease | 1475.7629 | 498 - 510 | 65 | 0.084 | GHYVQMVLMKLAA** |
| E9LLA5 | Polyprotein - NS2-3 protease | 1402.7465 | 499 - 510 | 65 | 0.084 | HYVQMVLMKLAA |
| E9LLA5 | Polyprotein - NS2-3 protease | 2210.1003 | 500 - 518 | 65 | 0.084 | YVQMVLMKLAALTGTYVYD** |
| E9LLA5 | Polyprotein - NS2-3 protease | 719.3710 | 501 - 506 | 65 | 0.084 | VQMVLM |
| E9LLA5 | Polyprotein - NS2-3 protease | 2134.1133 | 506 - 524 | 65 | 0.084 | MKLAALTGTYVYDHLTPLQ |
| E9LLA5 | Polyprotein - NS2-3 protease | 2249.1402 | 506 - 525 | 65 | 0.084 | MKLAALTGTYVYDHLTPLQD |
| E9LLA5 | Polyprotein - NS2-3 protease | 1756.8209 | 515 - 528 | 65 | 0.084 | YVYDHLTPLQDWAH |
| E9LLA5 | Polyprotein - NS2-3 protease | 1565.7647 | 536 - 549 | 65 | 0.084 | VAVEPVVFSDMETK** |
| E9LLA5 | Polyprotein - NS2-3 protease | 1724.7927 | 544 - 559 | 65 | 0.084 | SDMETKIITWGADTAA** |
| E9LLA5 | Polyprotein - NS2-3 protease | 848.4313 | 545 - 551 | 65 | 0.084 | DMETKII |
| E9LLA5 | Polyprotein - NS2-3 protease | 850.4470 | 546 - 552 | 65 | 0.084 | METKIIT** |
| J7HHX1 | NS3 protein (3) | 1147.5986 | 2 – 11 | 70 | 0.012 | PITAYAQQTR |
| J7HHX1 | NS3 protein (3) | 1643.9207 | 8 – 23 | 70 | 0.012 | QQTRGLLGTIVTSLTG |
| J7HHX1 | NS3 protein (3) | 1501.8464 | 17 - 30 | 70 | 0.012 | IVTSLTGRDKNVVT |
| J7HHX1 | NS3 protein (3) | 1514.8053 | 21 - 34 | 70 | 0.012 | LTGRDKNVVTGEVQ |
| J7HHX1 | NS3 protein (3) | 1115.5935 | 24 - 33 | 70 | 0.012 | RDKNVVTGEV |
| J7HHX1 | NS3 protein (3) | 860.4240 | 25 - 32 | 70 | 0.012 | DKNVVTGE |
| J7HHX1 | NS3 protein (3) | 844.4291 | 27 - 34 | 70 | 0.012 | NVVTGEVQ |
| J7HHX1 | NS3 protein (3) | 1131.5772 | 31 - 41 | 70 | 0.012 | GEVQVLSTATQ |
| J7HHX1 | NS3 protein (3) | 1435.7559 | 32 - 44 | 70 | 0.012 | EVQVLSTATQTFL |
| J7HHX1 | NS3 protein (3) | 1971.9612 | 37 - 55 | 70 | 0.012 | STATQTFLGTTVGGVMWTV** |
| J7HHX1 | NS3 protein (3) | 2290.1529 | 44 - 66 | 70 | 0.012 | LGTTVGGVMWTVYHGAGSRTLAG |
| J7HHX1 | NS3 protein (3) | 2191.0845 | 46 - 67 | 70 | 0.012 | TTVGGVMWTVYHGAGSRTLAGA |
| J7HHX1 | NS3 protein (3) | 1789.8934 | 48 - 64 | 70 | 0.012 | VGGVMWTVYHGAGSRTL |
| J7HHX1 | NS3 protein (3) | 1979.0126 | 53 - 71 | 70 | 0.012 | WTVYHGAGSRTLAGAKHPA |
| J7HHX1 | NS3 protein (3) | 1833.9598 | 56 - 73 | 70 | 0.012 | YHGAGSRTLAGAKHPALQ |
| J7HHX1 | NS3 protein (3) | 1628.8344 | 64 - 78 | 70 | 0.012 | LAGAKHPALQMYTNV** |
| J7HHX1 | NS3 protein (3) | 2014.9782 | 67 - 84 | 70 | 0.012 | AKHPALQMYTNVDQDLVG** |
| J7HHX1 | NS3 protein (3) | 975.4484 | 69 - 76 | 70 | 0.012 | HPALQMYT** |
| J7HHX1 | NS3 protein (3) | 2503.2053 | 71 - 93 | 70 | 0.012 | ALQMYTNVDQDLVGWPAPPGTKS** |
| J7HHX1 | NS3 protein (3) | 1564.8250 | 80 - 94 | 70 | 0.012 | QDLVGWPAPPGTKSL |
| J7HHX1 | NS3 protein (3) | 2214.0086 | 81 - 101 | 70 | 0.012 | DLVGWPAPPGTKSLEPCSCGS |
| J7HHX1 | NS3 protein (3) | 1450.7820 | 82 - 95 | 70 | 0.012 | LVGWPAPPGTKSLE |
| J7HHX1 | NS3 protein (3) | 2030.0909 | 101 - 118 | 70 | 0.012 | SADLYLVTRDADVIPARR |
| J7HHX1 | NS3 protein (3) | 1487.8096 | 105 - 117 | 70 | 0.012 | YLVTRDADVIPAR |
| J7HHX1 | NS3 protein (3) | 1857.0333 | 105 - 120 | 70 | 0.012 | YLVTRDADVIPARRRG |
| J7HHX1 | NS3 protein (3) | 1226.6844 | 113 - 123 | 70 | 0.012 | VIPARRRGDST |
| J7HHX1 | NS3 protein (3) | 1142.5931 | 121 - 131 | 70 | 0.012 | DSTASLLSPRP |
| J7HHX1 | NS3 protein (3) | 2214.1501 | 123 - 144 | 70 | 0.012 | TASLLSPRPLACLKGSSGGPVM** |
| J7HHX1 | NS3 protein (3) | 1353.7802 | 125 - 136 | 70 | 0.012 | SLLSPRPLACLK |
| J7HHX1 | NS3 protein (3) | 1498.7926 | 127 - 141 | 70 | 0.012 | LSPRPLACLKGSSGG |
| J7HHX1 | NS3 protein (3) | 912.4851 | 128 - 135 | 70 | 0.012 | SPRPLACL |
| J7HHX1 | NS3 protein (3) | 1040.5801 | 128 - 136 | 70 | 0.012 | SPRPLACLK |
| D2JVF5 | Polyprotein - NS3protein | 866.4208 | 3 – 9 | 76 | 0.003 | AYDIIIC |
| D2JVF5 | Polyprotein - NS3protein | 2057.9762 | 7 - 25 | 76 | 0.003 | IICDECHAVDATTILGIGT |
| D2JVF5 | Polyprotein - NS3protein | 713.3344 | 13 - 19 | 76 | 0.003 | HAVDATT |
| D2JVF5 | Polyprotein - NS3protein | 2367.2646 | 25 - 48 | 76 | 0.003 | TVLDQAETAGVRLTVLATATPPGS |
| D2JVF5 | Polyprotein - NS3protein | 2230.1481 | 38 - 59 | 76 | 0.003 | TVLATATPPGSVTTPHPNIEEV |
| D2JVF5 | Polyprotein - NS3protein | 2599.3494 | 38 - 63 | 76 | 0.003 | TVLATATPPGSVTTPHPNIEEVALGQ |
| D2JVF5 | Polyprotein - NS3protein | 1114.5870 | 40 - 51 | 76 | 0.003 | V.LATATPPGSVTT.P |
| D2JVF5 | Polyprotein - NS3protein | 2214.1532 | 40 - 61 | 76 | 0.003 | LATATPPGSVTTPHPNIEEVAL |
| D2JVF5 | Polyprotein - NS3protein | 1098.5557 | 41 - 52 | 76 | 0.003 | ATATPPGSVTTP |
| D2JVF5 | Polyprotein - NS3protein | 1516.7522 | 43 - 57 | 76 | 0.003 | ATPPGSVTTPHPNIE |
| D2JVF5 | Polyprotein - NS3protein | 1473.7100 | 45 - 58 | 76 | 0.003 | PPGSVTTPHPNIEE |
| D2JVF5 | Polyprotein - NS3protein | 2583.2493 | 48 - 71 | 76 | 0.003 | SVTTPHPNIEEVALGQEGEIPFYG |
| D2JVF5 | Polyprotein - NS3protein | 1789.8846 | 49 - 65 | 76 | 0.003 | VTTPHPNIEEVALGQEG |
| D2JVF5 | Polyprotein - NS3protein | 2535.2758 | 52 - 74 | 76 | 0.003 | PHPNIEEVALGQEGEIPFYGRAI |
| D2JVF5 | Polyprotein - NS3protein | 2566.3373 | 82 - 103 | 76 | 0.003 | GRHLIFCHSKKKCDELAAALRG |
| D2JVF5 | Polyprotein - NS3protein | 1040.5226 | 84 - 91 | 76 | 0.003 | HLIFCHSK |
| D2JVF5 | Polyprotein - NS3protein | 1870.9505 | 89 - 105 | 76 | 0.003 | HSKKKCDELAAALRGMG |
| D2JVF5 | Polyprotein - NS3protein | 1273.6812 | 92 - 102 | 76 | 0.003 | KKCDELAAALR |
| D2JVF5 | Polyprotein - NS3protein | 1518.7646 | 92 - 105 | 76 | 0.003 | KKCDELAAALRGMG |
| D2JVF5 | Polyprotein - NS3protein | 1832.9236 | 92 - 108 | 76 | 0.003 | KKCDELAAALRGMGLNA** |
| D2JVF5 | Polyprotein - NS3protein | 876.4011 | 93 - 100 | 76 | 0.003 | KCDELAAA |
| D2JVF5 | Polyprotein - NS3protein | 2126.0578 | 95 - 114 | 76 | 0.003 | DELAAALRGMGLNAVAYYRG** |
| D2JVF5 | Polyprotein - NS3protein | 799.4552 | 96 - 103 | 76 | 0.003 | ELAAALRG |
| D2JVF5 | Polyprotein - NS3protein | 1634.8450 | 96 - 111 | 76 | 0.003 | ELAAALRGMGLNAVAY** |
| D2JVF5 | Polyprotein - NS3protein | 2409.2474 | 96 - 118 | 76 | 0.003 | ELAAALRGMGLNAVAYYRGLDVS |
| D2JVF5 | Polyprotein - NS3protein | 1270.6128 | 103 - 114 | 76 | 0.003 | GMGLNAVAYYRG |
| D2JVF5 | Polyprotein - NS3protein | 1498.7238 | 103 - 116 | 76 | 0.003 | GMGLNAVAYYRGLD |
| D2JVF5 | Polyprotein - NS3protein | 1457.6973 | 104 - 116 | 76 | 0.003 | MGLNAVAYYRGLD** |
| D2JVF5 | Polyprotein - NS3protein | 1540.7708 | 104 - 117 | 76 | 0.003 | MGLNAVAYYRGLDV |
| D2JVF5 | Polyprotein - NS3protein | 1953.0030 | 104 - 121 | 76 | 0.003 | MGLNAVAYYRGLDVSVIP** |
| D2JVF5 | Polyprotein - NS3protein | 2078.1048 | 111 - 129 | 76 | 0.003 | YYRGLDVSVIPTQGDVVVV |
| D2JVF5 | Polyprotein - NS3protein | 2214.1454 | 114 - 135 | 76 | 0.003 | GLDVSVIPTQGDVVVVATDALM** |
| D2JVF5 | Polyprotein - NS3protein | 2133.1028 | 119 - 139 | 76 | 0.003 | VIPTQGDVVVVATDALMTGFT |
| D2JVF5 | Polyprotein - NS3protein | 2107.0508 | 120 - 140 | 76 | 0.003 | IPTQGDVVVVATDALMTGFTG** |
| D2JVF5 | Polyprotein - NS3protein | 1537.7698 | 125 - 139 | 76 | 0.003 | DVVVVATDALMTGFT |
| D2JVF5 | Polyprotein - NS3protein | 2074.9406 | 125 - 144 | 76 | 0.003 | DVVVVATDALMTGFTGDFDS** |
| D2JVF5 | Polyprotein - NS3protein | 2158.0141 | 125 - 145 | 76 | 0.003 | DVVVVATDALMTGFTGDFDSV |
| D2JVF5 | Polyprotein - NS3protein | 2644.2401 | 126 - 150 | 76 | 0.003 | VVVVATDALMTGFTGDFDSVIDCNV |
| D2JVF5 | Polyprotein - NS3protein | 1118.4591 | 134 - 143 | 76 | 0.003 | LMTGFTGDFD** |
| D2JVF5 | Polyprotein - NS3protein | 1304.5595 | 134 - 145 | 76 | 0.003 | LMTGFTGDFDSV** |
| D2JVF5 | Polyprotein - NS3protein | 2141.9940 | 137 - 156 | 76 | 0.003 | GFTGDFDSVIDCNVAVTQVV |
| D2JVF5 | Polyprotein - NS3protein | 1453.7341 | 155 - 167 | 76 | 0.003 | VVDFSLDPTFTIT |
| D2JVF5 | Polyprotein - NS3protein | 1518.7566 | 162 - 175 | 76 | 0.003 | PTFTITTQTVPQDA |
| D2JVF5 | Polyprotein - NS3protein | 2569.3658 | 167 - 189 | 76 | 0.003 | TTQTVPQDAVSRSQRRGRTGRGR |
| D2JVF5 | Polyprotein - NS3protein | 856.4291 | 169 - 176 | 76 | 0.003 | QTVPQDAV |
| D2JVF5 | Polyprotein - NS3protein | 1142.5792 | 172 - 181 | 76 | 0.003 | PQDAVSRSQR |
| D2JVF5 | Polyprotein - NS3protein | 1129.6177 | 179 - 188 | 76 | 0.003 | SQRRGRTGRG |
| D2JVF5 | Polyprotein - NS3protein | 1541.8063 | 186 - 199 | 76 | 0.003 | GRGRLGTYRYVSTG |
| D2JVF5 | Polyprotein - NS3protein | 1170.6145 | 188 - 197 | 76 | 0.003 | GRLGTYRYVS |
| D2JVF5 | Polyprotein - NS3protein | 844.4079 | 193 - 199 | 76 | 0.003 | YRYVSTG |
| D2JVF5 | Polyprotein - NS3protein | 1129.5516 | 193 - 201 | 76 | 0.003 | YRYVSTGER |
| K7XN61 | NS3-4A proteins | 2133.1252 | 1 – 20 | 83 | 0.0007 | APITAYAQQTRGLLGCIITS |
| K7XN61 | NS3-4A proteins | 1516.8032 | 7 – 20 | 83 | 0.0007 | AQQTRGLLGCIITS |
| K7XN61 | NS3-4A proteins | 1516.7845 | 18 - 31 | 83 | 0.0007 | ITSLTGRDKNQVEG |
| K7XN61 | NS3-4A proteins | 1870.9749 | 20 - 36 | 83 | 0.0007 | SLTGRDKNQVEGEVQIV |
| K7XN61 | NS3-4A proteins | 2230.1190 | 22 - 42 | 83 | 0.0007 | TGRDKNQVEGEVQIVSTAAQT |
| K7XN61 | NS3-4A proteins | 2566.2408 | 29 - 52 | 83 | 0.0007 | VEGEVQIVSTAAQTFLATCINGVC |
| K7XN61 | NS3-4A proteins | 1453.6871 | 37 - 50 | 83 | 0.0007 | STAAQTFLATCING |
| K7XN61 | NS3-4A proteins | 1541.7007 | 42 - 54 | 83 | 0.0007 | TFLATCINGVCWT |
| K7XN61 | NS3-4A proteins | 1304.5972 | 48 - 58 | 83 | 0.0007 | INGVCWTVYHG |
| K7XN61 | NS3-4A proteins | 2214.1004 | 50 - 70 | 83 | 0.0007 | GVCWTVYHGAGTRTLASPKGP |
| K7XN61 | NS3-4A proteins | 2058.0105 | 52 - 70 | 83 | 0.0007 | CWTVYHGAGTRTLASPKGP |
| K7XN61 | NS3-4A proteins | 1360.6888 | 53 - 64 | 83 | 0.0007 | WTVYHGAGTRTL |
| K7XN61 | NS3-4A proteins | 1120.5587 | 69 - 78 | 83 | 0.0007 | GPVIQMYTNV |
| K7XN61 | NS3-4A proteins | 2003.9663 | 70 - 86 | 83 | 0.0007 | PVIQMYTNVDQDLIGWP** |
| K7XN61 | NS3-4A proteins | 1226.5932 | 77 - 87 | 83 | 0.0007 | NVDQDLIGWPA |
| K7XN61 | NS3-4A proteins | 827.4178 | 80 - 86 | 83 | 0.0007 | QDLIGWP |
| K7XN61 | NS3-4A proteins | 1540.7457 | 85 - 98 | 83 | 0.0007 | WPAPQGARSLTPCT |
| K7XN61 | NS3-4A proteins | 1537.8188 | 144 - 158 | 83 | 0.0007 | LCPAGHAVGIFRAAV |
| K7XN61 | NS3-4A proteins | 2367.2416 | 145 - 167 | 83 | 0.0007 | CPAGHAVGIFRAAVCTRGVAKAV |
| K7XN61 | NS3-4A proteins | 2409.2250 | 165 - 185 | 83 | 0.0007 | KAVDFIPVENLETTMRSPVFT** |
| K7XN61 | NS3-4A proteins | 1423.6541 | 168 - 179 | 83 | 0.0007 | DFIPVENLETTM** |
| K7XN61 | NS3-4A proteins | 1146.5703 | 174 - 183 | 83 | 0.0007 | NLETTMRSPV |
| K7XN61 | NS3-4A proteins | 2077.9990 | 175 - 193 | 83 | 0.0007 | LETTMRSPVFTDNSTPPAV** |
| K7XN61 | NS3-4A proteins | 1514.7075 | 229 - 243 | 83 | 0.0007 | NPSVAATLGFGTYMS |
| K7XN61 | NS3-4A proteins | 1518.7388 | 231 - 245 | 83 | 0.0007 | SVAATLGFGTYMSKA** |
| K7XN61 | NS3-4A proteins | 1360.6697 | 232 - 244 | 83 | 0.0007 | VAATLGFGTYMSK** |
| K7XN61 | NS3-4A proteins | 2075.0034 | 232 - 251 | 83 | 0.0007 | VAATLGFGTYMSKAYGIDPN |
| K7XN61 | NS3-4A proteins | 1904.8978 | 234 - 251 | 83 | 0.0007 | ATLGFGTYMSKAYGIDPN |
| K7XN61 | NS3-4A proteins | 1040.5615 | 247 - 256 | 83 | 0.0007 | GIDPNIRTGV |
| K7XN61 | NS3-4A proteins | 1634.8192 | 259 - 273 | 83 | 0.0007 | ITTGSPITYSTYGKF |
| K7XN61 | NS3-4A proteins | 866.3956 | 272 - 279 | 83 | 0.0007 | KFLADGGC |
| K7XN61 | NS3-4A proteins | 876.3899 | 285 - 291 | 83 | 0.0007 | DIIICDE |
| K7XN61 | NS3-4A proteins | 1232.5343 | 291 - 301 | 83 | 0.0007 | ECHSTDATSIL |
| K7XN61 | NS3-4A proteins | 1170.5881 | 293 - 304 | 83 | 0.0007 | HSTDATSILGIG |
| K7XN61 | NS3-4A proteins | 1789.8945 | 294 - 311 | 83 | 0.0007 | STDATSILGIGTVLDQAE |
| K7XN61 | NS3-4A proteins | 1273.6766 | 296 - 308 | 83 | 0.0007 | DATSILGIGTVLD |
| K7XN61 | NS3-4A proteins | 1114.6234 | 299 - 309 | 83 | 0.0007 | SILGIGTVLDQ |
| K7XN61 | NS3-4A proteins | 1129.5727 | 308 - 318 | 83 | 0.0007 | DQAETAGARLV |
| K7XN61 | NS3-4A proteins | 879.4451 | 327 - 335 | 83 | 0.0007 | GSVTVPHPS |
| K7XN61 | NS3-4A proteins | 1248.6238 | 338 - 349 | 83 | 0.0007 | EVGLSTTGEIPF |
| K7XN61 | NS3-4A proteins | 2126.0626 | 362 - 378 | 83 | 0.0007 | GRHLIFCHSRKKCDELA |
| K7XN61 | NS3-4A proteins | 844.4378 | 363 - 368 | 83 | 0.0007 | RHLIFC |
| K7XN61 | NS3-4A proteins | 2569.3080 | 364 - 385 | 83 | 0.0007 | HLIFCHSRKKCDELAAKLVGMG |
| K7XN61 | NS3-4A proteins | 1518.7898 | 372 - 385 | 83 | 0.0007 | KKCDELAAKLVGMG |
| K7XN61 | NS3-4A proteins | 1142.5430 | 382 - 392 | 83 | 0.0007 | VGMGVNAVAYY |
| K7XN61 | NS3-4A proteins | 856.4079 | 390 - 396 | 83 | 0.0007 | AYYRGLD |
| K7XN61 | NS3-4A proteins | 2130.1130 | 395 - 415 | 83 | 0.0007 | LDVSIIPTSGDVVVVATDALM** |
| K7XN61 | NS3-4A proteins | 872.3586 | 414 - 421 | 83 | 0.0007 | LMTGYTGD** |
| K7XN61 | NS3-4A proteins | 1118.4590 | 414 - 423 | 83 | 0.0007 | LMTGYTGDFD |
| K7XN61 | NS3-4A proteins | 1498.6610 | 429 - 441 | 83 | 0.0007 | NTCVTQTVDFSLD |
| K7XN61 | NS3-4A proteins | 1953.0729 | 451 - 467 | 83 | 0.0007 | LPQDAVSRTQRRGRTGR |
| K7XN61 | NS3-4A proteins | 1242.7130 | 458 - 467 | 83 | 0.0007 | RTQRRGRTGR |
| K7XN61 | NS3-4A proteins | 2583.2652 | 466 - 489 | 83 | 0.0007 | GRGKPGIYRFVAPGERPSGMFDSS** |
| K7XN61 | NS3-4A proteins | 2582.2952 | 468 - 491 | 83 | 0.0007 | GKPGIYRFVAPGERPSGMFDSSVL** |
| K7XN61 | NS3-4A proteins | 2210.0943 | 469 - 488 | 83 | 0.0007 | KPGIYRFVAPGERPSGMFDS |
| K7XN61 | NS3-4A proteins | 745.2047 | 492 - 496 | 83 | 0.0007 | CECYD |
| K7XN61 | NS3-4A proteins | 860.4127 | 503 - 510 | 83 | 0.0007 | ELTPAETT |
| K7XN61 | NS3-4A proteins | 1958.8655 | 517 - 532 | 83 | 0.0007 | MNTPGLPVCQDHLEFW** |
| K7XN61 | NS3-4A proteins | 1232.5285 | 524 - 532 | 83 | 0.0007 | VCQDHLEFW |
| K7XN61 | NS3-4A proteins | 1129.5880 | 537 - 546 | 83 | 0.0007 | TGLTRIDAHF |
| K7XN61 | NS3-4A proteins | 2599.2707 | 544 - 566 | 83 | 0.0007 | AHFLSQTKQSGENFPYLVAYQAT |
| K7XN61 | NS3-4A proteins | 1457.6827 | 553 - 565 | 83 | 0.0007 | SGENFPYLVAYQA |
| K7XN61 | NS3-4A proteins | 2142.0568 | 554 - 572 | 83 | 0.0007 | GENFPYLVAYQATVCARAL |
| K7XN61 | NS3-4A proteins | 2107.0925 | 557 - 575 | 83 | 0.0007 | FPYLVAYQATVCARALAPP |
| K7XN61 | NS3-4A proteins | 1270.5805 | 574 - 583 | 83 | 0.0007 | PPPSWDQMWK |
| K7XN61 | NS3-4A proteins | 1832.8702 | 575 - 588 | 83 | 0.0007 | PPSWDQMWKCLTRL** |
| K7XN61 | NS3-4A proteins | 748.4232 | 589 - 595 | 83 | 0.0007 | KPTLHGP |
| K7XN61 | NS3-4A proteins | 2115.1034 | 603 - 621 | 83 | 0.0007 | GAVQNEVTLTHPITKYIMT |
| K7XN61 | NS3-4A proteins | 2107.0152 | 613 - 630 | 83 | 0.0007 | HPITKYIMTCMSADLEVV |
| K7XN61 | NS3-4A proteins | 2158.0360 | 614 - 632 | 83 | 0.0007 | PITKYIMTCMSADLEVVTS |
| K7XN61 | NS3-4A proteins | 849.3071 | 618 - 623 | 83 | 0.0007 | YIMTCM** |
| K7XN61 | NS3-4A proteins | 731.2288 | 620 - 625 | 83 | 0.0007 | MTCMSA** |
| K7XN61 | NS3-4A proteins | 2535.3295 | 623 - 647 | 83 | 0.0007 | MSADLEVVTSTWVLVGGVLAALAAY |
| K7XN61 | NS3-4A proteins | 841.5062 | 634 - 641 | 83 | 0.0007 | WVLVGGVL |
| K7XN61 | NS3-4A proteins | 713.3054 | 646 - 651 | 83 | 0.0007 | AYCLST |
| K7XN61 | NS3-4A proteins | 799.4916 | 657 - 664 | 83 | 0.0007 | VGRIVLSG |
| K7XN61 | NS3-4A proteins | 1473.6810 | 672 - 682 | 83 | 0.0007 | KEVLYREFDEM** |
| K7XN61 | NS3-4A proteins | 1216.5434 | 674 - 682 | 83 | 0.0007 | VLYREFDEM** |
| K7XN61 | NS3-4A proteins | 798.2524 | 682 - 687 | 83 | 0.0007 | MEECSQ** |
| Q81592 | NS4A protein | 1762.8382 | 1 – 15 | 65 | 0.14 | HPVTKYIATCMQADL** |
| Q81592 | NS4A protein | 1756.8376 | 3 – 17 | 65 | 0.14 | VTKYIATCMQADLEV** |
| Q81592 | NS4A protein | 1540.7265 | 5 – 17 | 65 | 0.14 | KYIATCMQADLEV |
| Q81592 | NS4A protein | 724.2520 | 9 – 14 | 65 | 0.14 | TCMQAD |
| Q81592 | NS4A protein | 1540.6684 | 10 - 22 | 65 | 0.14 | CMQADLEVMTSTR |
| Q81592 | NS4A protein | 1849.0132 | 17 - 35 | 65 | 0.14 | VMTSTRVLAGGVLAAVAAY |
| Q81592 | NS4A protein | 1602.8763 | 18 - 34 | 65 | 0.14 | MTSTRVLAGGVLAAVAA** |
| Q81592 | NS4A protein | 1774.9764 | 22 - 39 | 65 | 0.14 | RVLAGGVLAAVAAYCLAT |
| Q81592 | NS4A protein | 1121.5539 | 25 - 36 | 65 | 0.14 | AGGVLAAVAAYC |
| Q81592 | NS4A protein | 1789.8967 | 34 - 49 | 65 | 0.14 | AYCLATGCVSIIGRLH |
| Q81592 | NS4A protein | 869.5083 | 50 - 57 | 65 | 0.14 | INQRAVVA |
| Q81592 | NS4A protein | 1402.7344 | 54 - 66 | 65 | 0.14 | AVVAPDKEVLYEA |
| Q81592 | NS4A protein | 855.4702 | 55 - 62 | 65 | 0.14 | VVAPDKEV |
| Q81592 | NS4A protein | 850.4436 | 60 - 66 | 65 | 0.14 | KEVLYEA |
| Q81592 | NS4A protein | 772.2915 | 64 - 69 | 65 | 0.14 | YEAFDE |
| Q81592 | NS4B protein | 831.4702 | 74 - 81 | 65 | 0.14 | ASKATLIE |
| Q81592 | NS4B protein | 1614.8577 | 74 - 88 | 65 | 0.14 | ASKATLIEEGQRIAE |
| Q81592 | NS4B protein | 1475.7290 | 77 - 89 | 65 | 0.14 | ATLIEEGQRIAEM** |
| Q81592 | NS4B protein | 1044.5750 | 83 - 91 | 65 | 0.14 | GQRIAEMLK |
| Q81592 | NS4B protein | 719.3887 | 86 - 91 | 65 | 0.14 | IAEMLK** |
| Q81592 | NS4B protein | 790.4258 | 86 - 92 | 65 | 0.14 | IAEMLKS |
| Q81592 | NS4B protein | 1714.9651 | 86 - 100 | 65 | 0.14 | IAEMLKSKIQGLLQQ** |
| Q81592 | NS4B protein | 1273.7428 | 89 - 99 | 65 | 0.14 | MLKSKIQGLLQ** |
| Q81592 | NS4B protein | 829.4294 | 103 - 109 | 65 | 0.14 | KQAQDIQ |
| Q81592 | NS4B protein | 1724.8734 | 106 - 120 | 65 | 0.14 | QDIQPAVQTSWPKVE |
| Q81592 | NS4B protein | 1143.5560 | 107 - 116 | 65 | 0.14 | DIQPAVQTSW |
| Q81592 | NS4B protein | 1516.7562 | 110 - 122 | 65 | 0.14 | PAVQTSWPKVEEF |
| Q81592 | NS4B protein | 1804.9148 | 111 - 125 | 65 | 0.14 | AVQTSWPKVEEFWAK |
| Q81592 | NS4B protein | 844.4443 | 113 - 119 | 65 | 0.14 | QTSWPKV |
| Q81592 | NS4B protein | 1206.5710 | 115 - 123 | 65 | 0.14 | SWPKVEEFW |
| Q1HFF3 | NS5A protein | 1115.6703 | 20 – 28 | 67 | 0.025 | KTWLQSKLL |
| Q1HFF3 | NS5A protein | 1880.0818 | 26 – 41 | 67 | 0.025 | KLLPRLPGVPFLSCQR |
| Q1HFF3 | NS5A protein | 1148.6203 | 40 – 48 | 67 | 0.025 | QRGYKGVWR |
| Q1HFF3 | NS5A protein | 772.3538 | 58 – 65 | 67 | 0.025 | PCGAQIAG |
| Q1HFF3 | NS5A protein | 1168.6135 | 63 – 73 | 67 | 0.025 | IAGHVKNGSMR |
| Q1HFF3 | NS5A protein | 1061.5288 | 68 – 77 | 67 | 0.025 | KNGSMRTVGP** |
| Q1HFF3 | NS5A protein | 1956.9628 | 97 - 113 | 67 | 0.025 | PCSPSPAPNYSRALWRV |
| Q1HFF3 | NS5A protein | 1064.5138 | 112 - 120 | 67 | 0.025 | RVAAEEYVE |
| Q1HFF3 | NS5A protein | 738.3072 | 115 - 120 | 67 | 0.025 | AEEYVE |
| Q1HFF3 | NS5A protein | 767.3636 | 141 - 147 | 67 | 0.025 | PCQVPAP |
| Q1HFF3 | NS5A protein | 768.3330 | 147 - 152 | 67 | 0.025 | PEFFTE |
| Q1HFF3 | NS5A protein | 736.3326 | 160 - 165 | 67 | 0.025 | RYAPAC |
| Q1HFF3 | NS5A protein | 869.4970 | 166 - 172 | 67 | 0.025 | KPLLRDE |
| Q1HFF3 | NS5A protein | 1213.5649 | 183 - 193 | 67 | 0.025 | VVGSQLPCEPE |
| Q1HFF3 | NS5A protein | 1977.0208 | 172 - 189 | 67 | 0.025 | EVTFQVGLNQYVVGSQLP |
| Q1HFF3 | NS5A protein | 2945.4515 | 183 - 210 | 67 | 0.025 | VVGSQLPCEPEPDAVVLASMLTDPAHIT |
| Q1HFF3 | NS5A protein | 1939.8907 | 189 - 206 | 67 | 0.025 | PCEPEPDAVVLASMLTDP |
| Q1HFF3 | NS5A protein | 1820.9342 | 194 - 211 | 67 | 0.025 | PDAVVLASMLTDPAHITA |
| Q1HFF3 | NS5A protein | 886.3855 | 201 - 208 | 67 | 0.025 | SMLTDPAH** |
| Q1HFF3 | NS5A protein | 1044.5676 | 210 - 219 | 67 | 0.025 | TAETAGRRLA |
| Q1HFF3 | NS5A protein | 1514.7689 | 218 - 233 | 67 | 0.025 | LARGSPPSLASSSASQ |
| Q1HFF3 | NS5A protein | 1230.6092 | 223 - 235 | 67 | 0.025 | PPSLASSSASQLS |
| Q1HFF3 | NS5A protein | 1516.8096 | 226 - 241 | 67 | 0.025 | LASSSASQLSAPSLKA |
| Q1HFF3 | NS5A protein | 1756.8890 | 232 - 247 | 67 | 0.025 | SQLSAPSLKATCTTRH |
| Q1HFF3 | NS5A protein | 714.3912 | 233 - 239 | 67 | 0.025 | QLSAPSL |
| Q1HFF3 | NS5A protein | 1147.5907 | 234 - 244 | 67 | 0.025 | LSAPSLKATCT |
| Q1HFF3 | NS5A protein | 1098.6073 | 254 - 262 | 67 | 0.025 | LIEANLLWQ |
| Q1HFF3 | NS5A protein | 1226.6659 | 254 - 263 | 67 | 0.025 | LIEANLLWQQ |
| Q1HFF3 | NS5A protein | 975.4808 | 265 - 273 | 67 | 0.025 | MGGNITRVE |
| Q1HFF3 | NS5A protein | 844.4403 | 266 - 273 | 67 | 0.025 | GGNITRVE |
| Q1HFF3 | NS5A protein | 1498.8719 | 269 - 281 | 67 | 0.025 | ITRVESENKVVIL |
| Q1HFF3 | NS5A protein | 1643.8883 | 275 - 288 | 67 | 0.025 | ENKVVILDSFDPLR |
| Q1HFF3 | NS5A protein | 1244.7016 | 277 - 287 | 67 | 0.025 | KVVILDSFDPL |
| Q1HFF3 | NS5A protein | 1471.8399 | 277 - 289 | 67 | 0.025 | KVVILDSFDPLRA |
| Q1HFF3 | NS5A protein | 1244.6765 | 279 - 289 | 67 | 0.025 | VILDSFDPLRA |
| Q1HFF3 | NS5A protein | 705.2453 | 289 - 295 | 67 | 0.025 | AEEDGGE |
| Q1HFF3 | NS5A protein | 2611.4420 | 295 - 316 | 67 | 0.025 | EVSAPAEILRKTRKFPRAMPIW** |
| Q1HFF3 | NS5A protein | 1183.6924 | 296 - 306 | 67 | 0.025 | VSAPAEILRKT |
| Q1HFF3 | NS5A protein | 855.4814 | 297 - 304 | 67 | 0.025 | SAPAEILR |
| Q1HFF3 | NS5A protein | 1454.8721 | 299 - 310 | 67 | 0.025 | PAEILRKTRKFP |
| Q1HFF3 | NS5A protein | 829.5021 | 300 - 306 | 67 | 0.025 | AEILRKT |
| Q1HFF3 | NS5A protein | 1271.7938 | 303 - 312 | 67 | 0.025 | LRKTRKFPRA |
| Q1HFF3 | NS5A protein | 871.4803 | 324 - 330 | 67 | 0.025 | PLLESWK |
| Q1HFF3 | NS5A protein | 843.4967 | 349 - 356 | 67 | 0.025 | APPIPPPR |
| Q1HFF3 | NS5A protein | 2114.2324 | 349 - 367 | 67 | 0.025 | APPIPPPRRKRTVVLTEST |
| Q1HFF3 | NS5A protein | 2379.3362 | 336 - 357 | 67 | 0.025 | PPVVHGCPLPPTKAPPIPPPRR |
| Q1HFF3 | NS5A protein | 1535.9300 | 344 - 357 | 67 | 0.025 | LPPTKAPPIPPPRR |
| Q1HFF3 | NS5A protein | 1789.9785 | 358 - 374 | 67 | 0.025 | KRTVVLTESTVSSALAE |
| Q1HFF3 | NS5A protein | 1096.5401 | 374 - 384 | 67 | 0.025 | ELATKTFGSSG |
| Q1HFF3 | NS5A protein | 753.3657 | 394 - 401 | 67 | 0.025 | TAPPGQPS |
| Q1HFF3 | NS5A protein | 790.3345 | 404 - 411 | 67 | 0.025 | GEVGSDVE |
| Q1HFF3 | NS5A protein | 723.2712 | 426 - 432 | 67 | 0.025 | PDFSDGS |
| Q1HFF4 | NS5A protein | 1634.9620 | 21 - 34 | 68 | 0.019 | TWLQSRLLPRLPGV |
| Q1HFF4 | NS5A protein | 1972.0717 | 27 - 43 | 68 | 0.019 | LLPRLPGVPFLSCQRGY |
| Q1HFF4 | NS5A protein | 2030.0884 | 29 - 46 | 68 | 0.019 | PRLPGVPFLSCQRGYKGV |
| Q1HFF4 | NS5A protein | 1833.9672 | 30 - 45 | 68 | 0.019 | RLPGVPFLSCQRGYKG |
| Q1HFF4 | NS5A protein | 1353.6863 | 34 - 44 | 68 | 0.019 | VPFLSCQRGYK |
| Q1HFF4 | NS5A protein | 1148.6203 | 40 - 48 | 68 | 0.019 | QRGYKGVWR |
| Q1HFF4 | NS5A protein | 2213.9980 | 49 - 69 | 68 | 0.019 | GDGIMQTTCPCGAQIAGHVKN |
| Q1HFF4 | NS5A protein | 794.4399 | 63 - 70 | 68 | 0.019 | IAGHVKNG |
| Q1HFF4 | NS5A protein | 1040.5549 | 66 - 74 | 68 | 0.019 | HVKNGSMRI |
| Q1HFF4 | NS5A protein | 1044.5750 | 70 - 79 | 68 | 0.019 | GSMRIVGPKT |
| Q1HFF4 | NS5A protein | 2191.0910 | 119 - 138 | 68 | 0.019 | VEVARVGDFHYVTGVTTDNI |
| Q1HFF4 | NS5A protein | 2409.1094 | 124 - 144 | 68 | 0.019 | VGDFHYVTGVTTDNIKCPCQV |
| Q1HFF4 | NS5A protein | 1142.5767 | 157 - 165 | 68 | 0.019 | RLHRYAPAC |
| Q1HFF4 | NS5A protein | 1270.6717 | 157 - 166 | 68 | 0.019 | RLHRYAPACK |
| Q1HFF4 | NS5A protein | 1068.5750 | 163 - 171 | 68 | 0.019 | PACKPLLRD |
| Q1HFF4 | NS5A protein | 869.4970 | 166 - 172 | 68 | 0.019 | KPLLRDE |
| Q1HFF4 | NS5A protein | 1879.9680 | 172 - 188 | 68 | 0.019 | EVTFQVGLNQYVVGSQL |
| Q1HFF4 | NS5A protein | 1213.5649 | 183 - 193 | 68 | 0.019 | VVGSQLPCEPE |
| Q1HFF4 | NS5A protein | 2210.0963 | 183 - 203 | 68 | 0.019 | VVGSQLPCEPEPDAVVLASML |
| Q1HFF4 | NS5A protein | 1030.5369 | 194 - 203 | 68 | 0.019 | PDAVVLASML** |
| Q1HFF4 | NS5A protein | 1115.5896 | 197 - 207 | 68 | 0.019 | VVLASMLTDPA |
| Q1HFF4 | NS5A protein | 912.4375 | 202 - 209 | 68 | 0.019 | MLTDPAHI** |
| Q1HFF4 | NS5A protein | 1979.0297 | 204 - 222 | 68 | 0.019 | TDPAHITAETAGRRLARGS |
| Q1HFF4 | NS5A protein | 1450.8117 | 208 - 220 | 68 | 0.019 | HITAETAGRRLAR |
| Q1HFF4 | NS5A protein | 855.5151 | 214 - 221 | 68 | 0.019 | AGRRLARG |
| Q1HFF4 | NS5A protein | 1423.8008 | 215 - 228 | 68 | 0.019 | GRRLARGSPPSLAS |
| Q1HFF4 | NS5A protein | 1514.7689 | 218 - 233 | 68 | 0.019 | LARGSPPSLASSSASQ |
| Q1HFF4 | NS5A protein | 1230.6092 | 223 - 235 | 68 | 0.019 | PPSLASSSASQLS |
| Q1HFF4 | NS5A protein | 2503.1609 | 228 - 251 | 68 | 0.019 | SSSASQLSAPSLKATCTTRHDSPD |
| Q1HFF4 | NS5A protein | 2214.0699 | 230 - 250 | 68 | 0.019 | SASQLSAPSLKATCTTRHDSP |
| Q1HFF4 | NS5A protein | 1147.5907 | 234 - 244 | 68 | 0.019 | LSAPSLKATCT |
| Q1HFF4 | NS5A protein | 1498.7780 | 253 - 264 | 68 | 0.019 | DLIEANLLWRQE |
| Q1HFF4 | NS5A protein | 1628.8344 | 254 - 267 | 68 | 0.019 | LIEANLLWRQEMGG |
| Q1HFF4 | NS5A protein | 748.3326 | 261 - 265 | 68 | 0.019 | WRQEM |
| Q1HFF4 | NS5A protein | 732.3402 | 271 - 276 | 68 | 0.019 | RVESEN |
| Q1HFF4 | NS5A protein | 1487.7872 | 275 - 287 | 68 | 0.019 | ENKVVILDSFDPL |
| Q1HFF4 | NS5A protein | 1643.8883 | 275 - 288 | 68 | 0.019 | ENKVVILDSFDPLR |
| Q1HFF4 | NS5A protein | 1514.8457 | 276 - 288 | 68 | 0.019 | NKVVILDSFDPLR |
| Q1HFF4 | NS5A protein | 1471.8399 | 277 - 289 | 68 | 0.019 | KVVILDSFDPLRA |
| Q1HFF4 | NS5A protein | 1272.7078 | 278 - 288 | 68 | 0.019 | VVILDSFDPLR |
| Q1HFF4 | NS5A protein | 975.4661 | 284 - 291 | 68 | 0.019 | FDPLRAEE |
| Q1HFF4 | NS5A protein | 829.3818 | 292 - 300 | 68 | 0.019 | DGGEVSVPA |
| Q1HFF4 | NS5A protein | 1226.7459 | 301 - 310 | 68 | 0.019 | EILRKTRKSP |
| Q1HFF4 | NS5A protein | 860.4650 | 307 - 313 | 68 | 0.019 | RKSPRAM** |
| Q1HFF4 | NS5A protein | 1564.8772 | 307 - 319 | 68 | 0.019 | RKSPRAMPIWARP |
| Q1HFF4 | NS5A protein | 2124.0938 | 308 - 325 | 68 | 0.019 | KSPRAMPIWARPDYNPPL** |
| Q1HFF4 | NS5A protein | 843.4425 | 312 - 318 | 68 | 0.019 | AMPIWAR |
| Q1HFF4 | NS5A protein | 844.4330 | 321 - 327 | 68 | 0.019 | YNPPLLE |
| Q1HFF4 | NS5A protein | 787.4116 | 333 - 339 | 68 | 0.019 | DYVPPVV |
| Q1HFF4 | NS5A protein | 1857.0625 | 339 - 356 | 68 | 0.019 | VHGSPLPPTKAPPIPPPR |
| Q1HFF4 | NS5A protein | 1270.7285 | 341 - 353 | 68 | 0.019 | GSPLPPTKAPPIP |
| Q1HFF4 | NS5A protein | 1535.9300 | 344 - 357 | 68 | 0.019 | LPPTKAPPIPPPRR |
| Q1HFF4 | NS5A protein | 1789.9785 | 358 - 374 | 68 | 0.019 | KRTVVLTESTVSSALAE |
| Q1HFF4 | NS5A protein | 2230.0601 | 369 - 392 | 68 | 0.019 | SSALAELATKTFGSSGSSAVDSGT |
| Q1HFF4 | NS5A protein | 1956.8549 | 391 - 411 | 68 | 0.019 | GTATAPPGQPSSDGEVGSDVE |
| Q1HFF4 | NS5A protein | 767.3160 | 412 - 418 | 68 | 0.019 | SYSSMPP |
| Q1HFG0 | NS5A protein | 1196.6917 | 20 - 29 | 65 | 0.047 | KTWPQSKLLP |
| Q1HFG0 | NS5A protein | 1068.5968 | 21 - 29 | 65 | 0.047 | TWPQSKLLP |
| Q1HFG0 | NS5A protein | 922.5964 | 25 - 32 | 65 | 0.047 | SKLLPRLP |
| Q1HFG0 | NS5A protein | 1972.0717 | 27 - 43 | 65 | 0.047 | LLPRLPGVPFLSCQRGY |
| Q1HFG0 | NS5A protein | 2030.0884 | 29 - 46 | 65 | 0.047 | PRLPGVPFLSCQRGYKGV |
| Q1HFG0 | NS5A protein | 1833.9672 | 30 - 45 | 65 | 0.047 | RLPGVPFLSCQRGYKG |
| Q1HFG0 | NS5A protein | 1353.6863 | 34 - 44 | 65 | 0.047 | VPFLSCQRGYK |
| Q1HFG0 | NS5A protein | 1148.6203 | 40 - 48 | 65 | 0.047 | QRGYKGVWR |
| Q1HFG0 | NS5A protein | 2213.9980 | 49 - 69 | 65 | 0.047 | GDGIMQTTCPCGAQIAGHVKN |
| Q1HFG0 | NS5A protein | 794.4399 | 63 - 70 | 65 | 0.047 | IAGHVKNG |
| Q1HFG0 | NS5A protein | 1040.5549 | 66 - 74 | 65 | 0.047 | HVKNGSMRI |
| Q1HFG0 | NS5A protein | 1044.5750 | 70 - 79 | 65 | 0.047 | GSMRIVGPKT |
| Q1HFG0 | NS5A protein | 1856.9533 | 100 - 116 | 65 | 0.047 | PSPAPSYSRALWRVAAE |
| Q1HFG0 | NS5A protein | 2124.0640 | 104 - 121 | 65 | 0.047 | PSYSRALWRVAAEEYVEV |
| Q1HFG0 | NS5A protein | 2409.1094 | 124 - 144 | 65 | 0.047 | VGDFHYVTGVTTDNIKCPCQV |
| Q1HFG0 | NS5A protein | 1142.5767 | 157 - 165 | 65 | 0.047 | RLHRYAPAC |
| Q1HFG0 | NS5A protein | 1270.6717 | 157 - 166 | 65 | 0.047 | RLHRYAPACK |
| Q1HFG0 | NS5A protein | 869.4970 | 166 - 172 | 65 | 0.047 | KPLLRDE |
| Q1HFG0 | NS5A protein | 1879.9680 | 172 - 188 | 65 | 0.047 | EVTFQVGLNQYVVGSQL |
| Q1HFG0 | NS5A protein | 1213.5649 | 183 - 193 | 65 | 0.047 | VVGSQLPCEPE |
| Q1HFG0 | NS5A protein | 2210.0963 | 183 - 203 | 65 | 0.047 | VVGSQLPCEPEPDAVVLASML |
| Q1HFG0 | NS5A protein | 1030.5369 | 194 - 203 | 65 | 0.047 | PDAVVLASML** |
| Q1HFG0 | NS5A protein | 1115.5896 | 197 - 207 | 65 | 0.047 | VVLASMLTDPA |
| Q1HFG0 | NS5A protein | 912.4375 | 202 - 209 | 65 | 0.047 | MLTDPAHI** |
| Q1HFG0 | NS5A protein | 1979.0297 | 204 - 222 | 65 | 0.047 | TDPAHITAETAGRRLARGS |
| Q1HFG0 | NS5A protein | 1450.8117 | 208 - 220 | 65 | 0.047 | HITAETAGRRLAR |
| Q1HFG0 | NS5A protein | 860.4464 | 210 - 217 | 65 | 0.047 | TAETAGRR |
| Q1HFG0 | NS5A protein | 855.5151 | 214 - 221 | 65 | 0.047 | AGRRLARG |
| Q1HFG0 | NS5A protein | 1423.8008 | 215 - 228 | 65 | 0.047 | GRRLARGSPPSLAS |
| Q1HFG0 | NS5A protein | 1514.7689 | 218 - 233 | 65 | 0.047 | LARGSPPSLASSSASQ |
| Q1HFG0 | NS5A protein | 1230.6092 | 223 - 235 | 65 | 0.047 | PPSLASSSASQLS |
| Q1HFG0 | NS5A protein | 2503.1609 | 228 - 251 | 65 | 0.047 | SSSASQLSAPSLKATCTTRHDSPD |
| Q1HFG0 | NS5A protein | 1147.5907 | 234 - 244 | 65 | 0.047 | LSAPSLKATCT |
| Q1HFG0 | NS5A protein | 1498.7780 | 253 - 264 | 65 | 0.047 | DLIEANLLWRQE |
| Q1HFG0 | NS5A protein | 748.3326 | 261 - 265 | 65 | 0.047 | WRQEM |
| Q1HFG0 | NS5A protein | 732.3402 | 271 - 276 | 65 | 0.047 | RVESEN |
| Q1HFG0 | NS5A protein | 1487.7872 | 275 - 287 | 65 | 0.047 | ENKVVILDSFDPL |
| Q1HFG0 | NS5A protein | 1643.8883 | 275 - 288 | 65 | 0.047 | ENKVVILDSFDPLR |
| Q1HFG0 | NS5A protein | 1514.8457 | 276 - 288 | 65 | 0.047 | NKVVILDSFDPLR |
| Q1HFG0 | NS5A protein | 1471.8399 | 277 - 289 | 65 | 0.047 | KVVILDSFDPLRA |
| Q1HFG0 | NS5A protein | 1272.7078 | 278 - 288 | 65 | 0.047 | VVILDSFDPLR |
| Q1HFG0 | NS5A protein | 975.4661 | 284 - 291 | 65 | 0.047 | FDPLRAEE |
| Q1HFG0 | NS5A protein | 829.3818 | 292 - 300 | 65 | 0.047 | DGGEVSVPA |
| Q1HFG0 | NS5A protein | 1454.8721 | 299 - 310 | 65 | 0.047 | PAEILRKTRKFP |
| Q1HFG0 | NS5A protein | 2015.1462 | 297 - 313 | 65 | 0.047 | SVPAEILRKTRKFPRAM** |
| Q1HFG0 | NS5A protein | 2191.1121 | 266 - 285 | 65 | 0.047 | GGNITRVESENKVVILDSFD |
| Q1HFG0 | NS5A protein | 2214.0699 | 230 - 250 | 65 | 0.047 | SASQLSAPSLKATCTTRHDSP |
| Q1HFG0 | NS5A protein | 1628.9660 | 303 - 315 | 65 | 0.047 | LRKTRKFPRAMPI** |
| Q1HFG0 | NS5A protein | 843.4425 | 312 - 318 | 65 | 0.047 | AMPIWAR |
| Q1HFG0 | NS5A protein | 844.4330 | 321 - 327 | 65 | 0.047 | YNPPLLE |
| Q1HFG0 | NS5A protein | 787.4116 | 333 - 339 | 65 | 0.047 | DYVPPVV |
| Q1HFG0 | NS5A protein | 1535.9300 | 344 - 357 | 65 | 0.047 | P.LPPTKAPPIPPPRR.K |
| Q1HFG0 | NS5A protein | 1634.9580 | 353 - 366 | 65 | 0.047 | PPPRRKRTVVLTES |
| Q1HFG0 | NS5A protein | 1789.9785 | 358 - 374 | 65 | 0.047 | KRTVVLTESTVSSALAE |
| Q1HFG0 | NS5A protein | 2230.0601 | 369 - 392 | 65 | 0.047 | SSALAELATKTFGSSGSSAVDSGT |
| Q1HFG0 | NS5A protein | 1956.8549 | 391 - 411 | 65 | 0.047 | GTATAPPGQPSSDGEVGSDVE |
| Q1HFG0 | NS5A protein | 767.3160 | 412 - 418 | 65 | 0.047 | SYSSMPP |
| A4UXV5 | Polyprotein - NS5A protein (1b) | 2191.1823 | 13 - 30 | 65 | 0.052 | CTVLSDFKTWLQSKLLPR |
| A4UXV5 | Polyprotein - NS5A protein (1b) | 1628.9766 | 19 - 31 | 65 | 0.052 | FKTWLQSKLLPRL |
| A4UXV5 | Polyprotein - NS5A protein(1b) | 1163.7390 | 23 - 32 | 65 | 0.052 | LQSKLLPRLP |
| A4UXV5 | Polyprotein - NS5A protein (1b) | 922.5964 | 25 - 32 | 65 | 0.052 | SKLLPRLP |
| A4UXV5 | Polyprotein - NS5A protein(1b) | 1501.8116 | 27 - 39 | 65 | 0.052 | LLPRLPGVPFFSC |
| A4UXV5 | Polyprotein - NS5A protein (1b) | 2210.1208 | 31 - 49 | 65 | 0.052 | LPGVPFFSCQRGYKGVWRG |
| A4UXV5 | Polyprotein - NS5A protein(1b) | 2214.0793 | 34 - 52 | 65 | 0.052 | VPFFSCQRGYKGVWRGDGV |
| A4UXV5 | Polyprotein - NS5A protein (1b) | 1148.6203 | 40 - 48 | 65 | 0.052 | QRGYKGVWR |
| A4UXV5 | Polyprotein - NS5A protein(1b) | 844.4192 | 45 - 52 | 65 | 0.052 | GVWRGDGV |
| A4UXV5 | Polyprotein - NS5A protein (1b) | 787.3977 | 46 - 52 | 65 | 0.052 | VWRGDGV |
| A4UXV5 | Polyprotein - NS5A protein(1b) | 1498.7926 | 61 - 74 | 65 | 0.052 | AQITGHVKNGSMKI** |
| A4UXV5 | Polyprotein - NS5A protein (1b) | 1880.0302 | 61 - 78 | 65 | 0.052 | AQITGHVKNGSMKIVGPK** |
| A4UXV5 | Polyprotein - NS5A protein(1b) | 1423.7606 | 64 - 77 | 65 | 0.052 | TGHVKNGSMKIVGP |
| A4UXV5 | Polyprotein - NS5A protein (1b) | 1450.8078 | 65 - 78 | 65 | 0.052 | GHVKNGSMKIVGPK |
| A4UXV5 | Polyprotein - NS5A protein(1b) | 860.4426 | 74 - 81 | 65 | 0.052 | IVGPKTCS |
| A4UXV5 | Polyprotein - NS5A protein (1b) | 2409.1423 | 90 - 111 | 65 | 0.052 | INAYTTGPCTPSPAPSYSRALW |
| A4UXV5 | Polyprotein - NS5A protein(1b) | 1856.9533 | 100 - 116 | 65 | 0.052 | PSPAPSYSRALWRVAAE |
| A4UXV5 | Polyprotein - NS5A protein (1b) | 794.4075 | 106 - 111 | 65 | 0.052 | YSRALW |
| A4UXV5 | Polyprotein - NS5A protein(1b) | 1956.8710 | 124 - 140 | 65 | 0.052 | VGDFHYVTGMTTDNIKC |
| A4UXV5 | Polyprotein - NS5A protein (1b) | 1142.5767 | 157 - 165 | 65 | 0.052 | RLHRYAPAC |
| A4UXV5 | Polyprotein - NS5A protein(1b) | 1270.6717 | 157 - 166 | 65 | 0.052 | RLHRYAPACK |
| A4UXV5 | Polyprotein - NS5A protein (1b) | 1068.5750 | 163 - 171 | 65 | 0.052 | PACKPLLRD |
| A4UXV5 | Polyprotein - NS5A protein(1b) | 869.4970 | 166 - 172 | 65 | 0.052 | KPLLRDE |
| A4UXV5 | Polyprotein - NS5A protein (1b) | 1435.7096 | 175 - 187 | 65 | 0.052 | FQVGLNQYPVGSQ |
| A4UXV5 | Polyprotein - NS5A protein(1b) | 1514.7651 | 197 - 210 | 65 | 0.052 | TVLTSMLTDPSHIT |
| A4UXV5 | Polyprotein - NS5A protein (1b) | 2015.0245 | 197 - 215 | 65 | 0.052 | TVLTSMLTDPSHITAETAK |
| A4UXV5 | Polyprotein - NS5A protein(1b) | 1213.6013 | 199 - 209 | 65 | 0.052 | LTSMLTDPSHI |
| A4UXV5 | Polyprotein - NS5A protein (1b) | 2214.1426 | 200 - 219 | 65 | 0.052 | TSMLTDPSHITAETAKRRLA** |
| A4UXV5 | Polyprotein - NS5A protein(1b) | 912.4375 | 202 - 209 | 65 | 0.052 | MLTDPSHI |
| A4UXV5 | Polyprotein - NS5A protein (1b) | 1044.6040 | 210 - 218 | 65 | 0.052 | TAETAKRRL |
| A4UXV5 | Polyprotein - NS5A protein(1b) | 1115.6411 | 210 - 219 | 65 | 0.052 | TAETAKRRLA |
| A4UXV5 | Polyprotein - NS5A protein (1b) | 1230.6092 | 223 - 235 | 65 | 0.052 | PPSLASSSASQLS |
| A4UXV5 | Polyprotein - NS5A protein(1b) | 1147.5907 | 234 - 244 | 65 | 0.052 | LSAPSLKATCT |
| A4UXV5 | Polyprotein - NS5A protein (1b) | 2503.1550 | 246 - 267 | 65 | 0.052 | HHDSPDADLIEANLLWRQEMGG |
| A4UXV5 | Polyprotein - NS5A protein(1b) | 748.3326 | 261 - 265 | 65 | 0.052 | WRQEM |
| A4UXV5 | Polyprotein - NS5A protein (1b) | 732.3402 | 271 - 276 | 65 | 0.052 | RVESEN |
| A4UXV5 | Polyprotein - NS5A protein(1b) | 2030.0684 | 272 - 289 | 65 | 0.052 | VESENKVVILDSFDPLRA |
| A4UXV5 | Polyprotein - NS5A protein (1b) | 1487.7872 | 275 - 287 | 65 | 0.052 | ENKVVILDSFDPL |
| A4UXV5 | Polyprotein - NS5A protein(1b) | 1643.8883 | 275 - 288 | 65 | 0.052 | ENKVVILDSFDPLR |
| A4UXV5 | Polyprotein - NS5A protein (1b) | 1514.8457 | 276 - 288 | 65 | 0.052 | NKVVILDSFDPLR |
| A4UXV5 | Polyprotein - NS5A protein(1b) | 1131.6176 | 277 - 286 | 65 | 0.052 | KVVILDSFDP |
| A4UXV5 | Polyprotein - NS5A protein (1b) | 1471.8399 | 277 - 289 | 65 | 0.052 | KVVILDSFDPLRA |
| A4UXV5 | Polyprotein - NS5A protein(1b) | 1272.7078 | 278 - 288 | 65 | 0.052 | VVILDSFDPLR |
| A4UXV5 | Polyprotein - NS5A protein (1b) | 975.4661 | 284 - 291 | 65 | 0.052 | FDPLRAEE |
| A4UXV5 | Polyprotein - NS5A protein(1b) | 2124.2241 | 297 - 315 | 65 | 0.052 | SVAAEILRKTRKFPPAMPI |
| A4UXV5 | Polyprotein - NS5A protein (1b) | 1454.8721 | 301 - 312 | 65 | 0.052 | EILRKTRKFPPA |
| A4UXV5 | Polyprotein - NS5A protein(1b) | 962.5008 | 306 - 313 | 65 | 0.052 | TRKFPPAM** |
| A4UXV5 | Polyprotein - NS5A protein (1b) | 1833.9236 | 310 - 325 | 65 | 0.052 | PPAMPIWARPDYNPPL |
| A4UXV5 | Polyprotein - NS5A protein(1b) | 1978.9975 | 311 - 327 | 65 | 0.052 | PAMPIWARPDYNPPLLE |
| A4UXV5 | Polyprotein - NS5A protein (1b) | 843.4425 | 312 - 318 | 65 | 0.052 | AMPIWAR |
| A4UXV5 | Polyprotein - NS5A protein(1b) | 1972.0968 | 335 - 353 | 65 | 0.052 | VPPVVHGCPLPPTKAPPIP |
| A4UXV5 | Polyprotein - NS5A protein (1b) | 1535.9300 | 344 - 357 | 65 | 0.052 | LPPTKAPPIPPPRR |
| A4UXV5 | Polyprotein - NS5A protein(1b) | 1634.9580 | 353 - 366 | 65 | 0.052 | PPPRRKRTVVLTES |
| A4UXV5 | Polyprotein - NS5A protein (1b) | 1789.9785 | 358 - 374 | 65 | 0.052 | KRTVVLTESTVSSALAE |
| A4UXV5 | Polyprotein - NS5A protein(1b) | 2230.0601 | 369 - 392 | 65 | 0.052 | SSALAELATKTFGSSGSSAVDSGT |
| A4UXV5 | Polyprotein - NS5A protein (1b) | 767.3160 | 412 - 418 | 65 | 0.052 | SYSSMPP |
| I0J2K8 | Polyprotein - NS5B protein | 1270.5902 | 4 – 13 | 76 | 0.0032 | TERDIRTEHD |
| I0J2K8 | Polyprotein - NS5B protein | 1423.6112 | 13 - 23 | 76 | 0.0032 | DIYQCCQLEPV |
| I0J2K8 | Polyprotein - NS5B protein | 2230.1238 | 24 - 42 | 76 | 0.0032 | ARKVITSLTERLYCGGPMY** |
| I0J2K8 | Polyprotein - NS5B protein | 2644.3214 | 24 - 46 | 76 | 0.0032 | ARKVITSLTERLYCGGPMYNSRG** |
| I0J2K8 | Polyprotein - NS5B protein | 2132.9983 | 28 - 45 | 76 | 0.0032 | ITSLTERLYCGGPMYNSR** |
| I0J2K8 | Polyprotein - NS5B protein | 2003.9193 | 29 - 45 | 76 | 0.0032 | TSLTERLYCGGPMYNSR |
| I0J2K8 | Polyprotein - NS5B protein | 1040.4961 | 30 - 37 | 76 | 0.0032 | SLTERLYC |
| I0J2K8 | Polyprotein - NS5B protein | 2074.9135 | 35 - 52 | 76 | 0.0032 | LYCGGPMYNSRGQSCGIR |
| I0J2K8 | Polyprotein - NS5B protein | 2114.8979 | 37 - 54 | 76 | 0.0032 | CGGPMYNSRGQSCGIRRC |
| I0J2K8 | Polyprotein - NS5B protein | 2582.2377 | 39 - 61 | 76 | 0.0032 | GPMYNSRGQSCGIRRCRASGVLT |
| I0J2K8 | Polyprotein - NS5B protein | 1540.6875 | 40 - 52 | 76 | 0.0032 | PMYNSRGQSCGIR** |
| I0J2K8 | Polyprotein - NS5B protein | 2057.9418 | 41 - 57 | 76 | 0.0032 | MYNSRGQSCGIRRCRAS |
| I0J2K8 | Polyprotein - NS5B protein | 2214.0317 | 41 - 59 | 76 | 0.0032 | MYNSRGQSCGIRRCRASGV |
| I0J2K8 | Polyprotein - NS5B protein | 1248.5928 | 45 - 54 | 76 | 0.0032 | RGQSCGIRRC |
| I0J2K8 | Polyprotein - NS5B protein | 2599.2782 | 49 - 71 | 76 | 0.0032 | CGIRRCRASGVLTTSLGNTLTCF |
| I0J2K8 | Polyprotein - NS5B protein | 2569.2992 | 56 - 80 | 76 | 0.0032 | ASGVLTTSLGNTLTCFLKASAACRA |
| I0J2K8 | Polyprotein - NS5B protein | 1953.0241 | 58 - 76 | 76 | 0.0032 | GVLTTSLGNTLTCFLKASA |
| I0J2K8 | Polyprotein - NS5B protein | 2142.0562 | 61 - 80 | 76 | 0.0032 | TTSLGNTLTCFLKASAACRA |
| I0J2K8 | Polyprotein - NS5B protein | 2566.2454 | 66 - 88 | 76 | 0.0032 | NTLTCFLKASAACRAANLKNPDM |
| I0J2K8 | Polyprotein - NS5B protein | 2367.1497 | 68 - 88 | 76 | 0.0032 | LTCFLKASAACRAANLKNPDM** |
| I0J2K8 | Polyprotein - NS5B protein | 2107.0302 | 69 - 87 | 76 | 0.0032 | TCFLKASAACRAANLKNPD |
| I0J2K8 | Polyprotein - NS5B protein | 866.4320 | 71 - 78 | 76 | 0.0032 | FLKASAAC |
| I0J2K8 | Polyprotein - NS5B protein | 1634.8562 | 71 - 85 | 76 | 0.0032 | FLKASAACRAANLKD |
| I0J2K8 | Polyprotein - NS5B protein | 1959.0029 | 72 - 89 | 76 | 0.0032 | LKASAACRAANLKNPDML** |
| I0J2K8 | Polyprotein - NS5B protein | 1457.7045 | 74 - 87 | 76 | 0.0032 | ASAACRAANLKNPD |
| I0J2K8 | Polyprotein - NS5B protein | 1518.6919 | 75 - 88 | 76 | 0.0032 | SAACRAANLKDPDM |
| I0J2K8 | Polyprotein - NS5B protein | 1360.6227 | 77 - 88 | 76 | 0.0032 | ACRAANLKDPDM |
| I0J2K8 | Polyprotein - NS5B protein | 1789.8273 | 77 - 92 | 76 | 0.0032 | ACRAANLKDPDMLVCG |
| I0J2K8 | Polyprotein - NS5B protein | 1904.8543 | 77 - 93 | 76 | 0.0032 | ACRAANLKDPDMLVCGD |
| I0J2K8 | Polyprotein - NS5B protein | 1304.5965 | 78 - 88 | 76 | 0.0032 | CRAANLKNPDM** |
| I0J2K8 | Polyprotein - NS5B protein | 1516.7490 | 78 - 90 | 76 | 0.0032 | CRAANLKNPDMLV** |
| I0J2K8 | Polyprotein - NS5B protein | 1832.8331 | 78 - 93 | 76 | 0.0032 | CRAANLKNPDMLVCGD |
| I0J2K8 | Polyprotein - NS5B protein | 1516.7490 | 79 - 91 | 76 | 0.0032 | RAANLKNPDMLVC** |
| I0J2K8 | Polyprotein - NS5B protein | 2535.1906 | 80 - 102 | 76 | 0.0032 | AANLKDPDMLVCGDDLIIISESM** |
| I0J2K8 | Polyprotein - NS5B protein | 1129.5801 | 81 - 90 | 76 | 0.0032 | ANLKNPDMLV** |
| I0J2K8 | Polyprotein - NS5B protein | 1146.5413 | 83 - 92 | 76 | 0.0032 | LKDPDMLVCG |
| I0J2K8 | Polyprotein - NS5B protein | 1360.6003 | 85 - 96 | 76 | 0.0032 | NPDMLVCGDDLI |
| I0J2K8 | Polyprotein - NS5B protein | 1473.6844 | 85 - 97 | 76 | 0.0032 | NPDMLVCGDDLII |
| I0J2K8 | Polyprotein - NS5B protein | 2077.9370 | 85 - 103 | 76 | 0.0032 | NPDMLVCGDDLIIISESMG |
| I0J2K8 | Polyprotein - NS5B protein | 2409.0750 | 85 - 106 | 76 | 0.0032 | NPDMLVCGDDLIIISESMGVSE** |
| I0J2K8 | Polyprotein - NS5B protein | 2213.9742 | 87 - 106 | 76 | 0.0032 | DMLVCGDDLIIISESMGVSE** |

*Modification – Carbamidomethyl (C), ** Modification - Oxidation (M).

**Supplementary Table S4.** **Data used in phylogenetic analysis**. Host species, virus abbreviations, sequence accession nos. and definition of the hepacivirus used in the phylogenetic analysis.

| **Virus** | **Host** | **GenBank accession no.** | **Definition** |
| --- | --- | --- | --- |
| HCV-1a | *Homo sapiens* | AF011751 | HCV strain H77 pCV-H77C polyprotein gene, complete cds |
| HCV-1b | *Homo sapiens* | AJ238799 | Hepatitis C virus type 1b complete genome, isolate Con1 |
| HCV-1b | *Homo sapiens* | AAL00900 | Polyprotein [Hepatitis C virus subtype 1b] |
| HCV-2a | *Homo sapiens* | D00944 | Hepatitis C virus genomic RNA for polyprotein, complete cds |
| HCV-2b | *Homo sapiens* | HPCJ8G | Hepatitis C virus genome |
| HCV-3 | *Homo sapiens* | JN588558 | Hepatitis C virus genotype 3 strain NCVI/PK1, complete genome |
| HCV-3a | *Homo sapiens* | D17763 | Hepatitis C virus (isolate NZL1) genomic RNA, complete genome |
| HCV-3b | *Homo sapiens* | KJ470617 | Hepatitis C virus isolate QC88, complete genome |
| HCV-3d | *Homo sapiens* | KJ470619 | Hepatitis C virus isolate NE274, complete genome |
| HCV-3e | *Homo sapiens* | AII25855 | Polyprotein [Hepatitis C virus] |
| HCV-3g | *Homo sapiens* | AFD18576 | Polyprotein precursor [Hepatitis C virus] |
| HCV-3k | *Homo sapiens* | D63821 | Hepatitis C virus (isolate JK049) genomic RNA, complete genome |
| HCV-4a | *Homo sapiens* | Y11604 | Hepatitis C virus type 4a RNA for HCV polyprotein |
| HCV-5a | *Homo sapiens* | Y13184 | Hepatitis C virus genotype 5a RNA for HCV polyprotein |
| HCV-6a | *Homo sapiens* | Y12083 | Hepatitis C virus genotype 6a RNA for HCV polyprotein |
| HCV-7a | *Homo sapiens* | EF108306 | Hepatitis C virus QC69 polyprotein gene, complete cds |
| CHV | Dog | JF744991 | Canine hepacivirus AAK-2011 polyprotein gene, complete cds |
| NPHV | Horse | JQ434001 | Hepacivirus AK-2012 isolate NPHV-NZP-1 polyprotein gene, complete cds |
| RHV | *Peromyscus maniculatus* | AGJ71779 | Polyprotein, [Rodent hepacivirus] |
| BHVcladeA | *Hipposideros vittatus* | KC796077 | Bat hepacivirus isolate PDB-112 polyprotein gene, complete cds |
| GHV | black-and-white *Colobus* | KC551800 | Guereza hepacivirus isolate GHV-1 BWC08, partial genome |

**Supplementary Table S5. Rabbit sequences blastp in the UniProt database**. Blastp in the UniProt database (http://www.uniprot.org/blast/) between all identified proteins of rabbit tested sample by MALDI TOF/TOF-MS/MS analysis, from complete sequences, constructed consensus sequences and constructed final consensus sequence as described in methods section.

| **Animal** | **UniProt accession no.** | **Strain (Genotype/isolate)** | **Protein/Polyprotein** | **Matches position (Amino –acid)** | **Identity (%)** | **E-value** |
| --- | --- | --- | --- | --- | --- | --- |
| DR | F4YQ96 | HCV | Core | 1 - 191 | 100.0 | 1.0 ×10 ^-139^ |
| DR | Q8V812 | HCV | Core | 1 - 191 | 99.0 | 1.0 ×10 ^-139^ |
| DR | Q8JWL3 | HCV | Core | 1 - 191 | 99.0 | 1.0 ×10 ^-139^ |
| DR | Q98V90 | HCV | E1 | 16 - 207 | 100.0 | 4.0 ×10 ^-139^ |
| DR | E9LL08 | HCV (1a) | E1 | 192 - 383 | 95.0 | 3.0 ×10 ^-132^ |
| DR | P27955 | HCV (isolate HCT27) | E1 | 64 - 255 | 95.0 | 2.0 ×10 ^-133^ |
| DR | D1KSI0 | HCV (3a) | E2 | 1 - 369 | 99.0 | 0.0 |
| DR | Q4KP55 | HCV | E2 | 214 - 582 | 91.0 | 0.0 |
| DR | D3W7L4 | HCV (1a) | p7 | 179 - 241 | 100.0 | 2.0 ×10 ^-41^ |
| DR | P26664 | HCV (1a) (isolate 1) | p7 | 747 - 809 | 95.0 | 1.0 ×10 ^-38^ |
| DR | Q5QRC3 | HCV (1b) | p7 | 1 - 63 | 87.0 | 3.0 ×10 ^-40^ |
| DR | F4YQP9 | HCV | NS2 | 4 - 216 | 94.0 | 1.0 ×10 ^-137^ |
| DR | Q5R2C5 | HCV (1b) | NS2 | 813 - 1025 | 91.0 | 2.0 ×10 ^-118^ |
| DR | K7Y470 | HCV | NS3 | 1 - 631 | 96.0 | 0.0 |
| DR | **Q9WMX2** | HCV (1b) (isolate Con1) | NS3 | 1027 - 1657 | 93.0 | 0.0 |
| DR | Q68240 | HCV | NS4A | 32 - 80 | 92.0 | 3.0 ×10 ^-26^ |
| DR | Q68586 | HCV | NS4B | 1 - 196 | 100.0 | 6.0 ×10 ^-133^ |
| DR | **Q81258** | HCV (3a) (isolate NZL1) | NS4B | 1769 - 1964 | 96.0 | 5.0 ×10 ^-115^ |
| DR | I6S4Q1 | HCV (1a) | NS4B | 52 - 247 | 87.0 | 1.0 ×10 ^-117^ |
| DR | A9JKN5 | HCV | NS5A | 3 - 439 | 97.0 | 0.0 |
| DR | R4IH56 | HCV (3a) | NS5A | 3 - 439 | 93.0 | 0.0 |
| DR | M9UX90 | HCV (3b) | NS5B | 464 - 1041 | 92.0 | 0.0 |
| DR | **Q81487** | HCV (3b) (isolate Tr-Kj) | NS5B | 2444 - 3021 | 89.0 | 0.0 |
| DR | K7PM37 | HCV (3g) | NS5B | 2443 - 3020 | 85.0 | 0.0 |
| DR | K7PPJ5 | HCV (3i) | NS5B | 2442 - 3019 | 85.0 | 0.0 |
| DR | **Q68801** | HCV (3k) (isolate JK049) | NS5B | 2440 - 3017 | 81.0 | 0.0 |
| DR | Q1ZZ55 | HCV (4a) | NS5B | 2429 - 3006 | 77.0 | 0.0 |
| DR* | C4MR63 | HCV (1a) | Genome polyprotein | 1 - 3009 | 78.0 | 0.0 |
| DR* | **P26664** | HCV (1a) (isolate 1) | Genome polyprotein | 1 - 3009 | 77.0 | 0.0 |
| DR* | **Q9WMX2** | HCV (1b) (isolate Con1) | Genome polyprotein | 1 - 3008 | 78.0 | 0.0 |
| DR* | **P26662** | HCV (1b) (isolate Japanese) | Genome polyprotein | 1 - 3008 | 77.0 | 0.0 |

DR - Domestic rabbit *(Oryctolagus cuniculus)*, *Final consensus sequence - RHCV.

**Supplementary Table S6. Haret sequences blastp in the UniProt database. Blastp in the UniProt database (http://www.uniprot.org/blast/) between all identified proteins for hare tested sample by MALDI TOF/TOF-MS/MS analysis, from complete sequences, constructed consensus sequences and constructed final consensus sequence as described in methods section.**

| **Animal** | **UniProt accession no.** | **Strain (Genotype/isolate)** | **Protein/Polyprotein** | **Matches position (amino-acid)** | **Identity (%)** | **E-value** |
| --- | --- | --- | --- | --- | --- | --- |
| H | Q8V7P2 | HCV | Core | 6 - 191 | 80.0 | 7.0 ×10 ^-98^ |
| H | Q8JWM5 | HCV (1b) | Core | 6 - 191 | 80.0 | 2.0 ×10 ^-97^ |
| H | Q8V812 | HCV | Core | 6 - 191 | 80.0 | 3.0 ×10 ^-97^ |
| H | Q8JWL3 | HCV (1b) | Core | 6 - 191 | 80.0 | 3.0 ×10 ^-97^ |
| H | B6USQ0 | HCV | E1 | 11 - 189 | 91.0 | 6.0 ×10 ^-108^ |
| H | B6USQ9 | HCV | E1 | 11 - 189 | 91.0 | 6.0 ×10 ^-108^ |
| H | B6USP8 | HCV | E1 | 11 - 189 | 90.0 | 7.0 ×10 -107 |
| H | E9LLA5 | HCV (1b) | E2 | 1 - 313 | 78.0 | 8.0 ×10 ^-166^ |
| H | J7JNP9 | HCV | P7 | 6 - 57 | 100.0 | 3.0 ×10 ^-31^ |
| H | Q0JXL6 | HCV | p7 | 1 - 52 | 98.0 | 2.0 ×10 ^-34^ |
| H | Q5QRR7 | HCV (1b) | p7 | 1 - 52 | 98.0 | 2.0 ×10 ^-34^ |
| H | E9LLA5 | HCV (1b) | NS2 | 382 - 559 | 96.0 | 6.0 ×10 ^-109^ |
| H | P2984& | HCV (1b) (isolate Taiwan) | NS2 | 815 - 992 | 86.0 | 4.0 ×10 ^-92^ |
| H | **Q9WMX2** | HCV (1b) (isolate Con1) | NS2 | 815 - 992 | 85.0 | 5.0 ×10 ^-91^ |
| H | K7YE43 | HCV | NS3 | 1 - 630 | 82.0 | 0.0 |
| H | A8DFW2 | HCV (1a) | NS3 | 1027 - 1656 | 82.0 | 0.0 |
| H | **P27958** | HCV (1a) (isolate H) | NS3 | 1027 - 1656 | 81.0 | 0.0 |
| H | Q68236 | HCV | NS4A | 31 - 85 | 78.0 | 1.0 ×10 ^-25^ |
| H | E9LLF1 | HCV | NS4A | 142 - 196 | 78.0 | 2.0 ×10 ^-24^ |
| H | Q81592 | HCV | NS4B | 74 - 125 | 100.0 | 2.0 ×10 ^-34^ |
| H | P26660 | HCV (2a)(isolate HC-J6) | NS4B | 1716 - 1767 | 92.0 | 2.0 ×10 ^-29^ |
| H | P26661 | HCV (2b) (isolate HC-J8) | NS4B | 1716 - 1767 | 85.0 | 1.0 ×10 ^-27^ |
| H | Q1HFF3 | HCV | NS5A | 13 - 432 | 95.0 | 0.0 |
| H | Q5L452 | HCV | NS5A | 13 - 432 | 94.0 | 0.0 |
| H | U5I9Z1 | HCV (1b) | NS5A | 13 - 432 | 89.0 | 0.0 |
| H | I0J2K8 | HCV | NS5B | 4 - 106 | 100.0 | 1.0 ×10 ^-68^ |
| H | D8VNF7 | HCV | NS5B | 2 - 104 | 94.0 | 1.0 ×10 ^-65^ |
| H | A4ZU81 | HCV (6q) | NS5B | 2653 - 2755 | 91.0 | 2.0 ×10 ^-56^ |
| H | E7E044 | HCV (6) | NS5B | 113 - 215 | 84.0 | 2.0 ×10 ^-57^ |
| H* | P26662 | HCV (1b) (isolate Japanese | Genome polyprotein | 6 - 1788 | 72.0 | 0.0 |
| H* | Q9DTE7 | HCV | Genome polyprotein | 6 - 1788 | 72.0 | 0.0 |
| H* | A8DFW2 | HCV (1a) | Genome polyprotein | 6 - 1788 | 70.0 | 0.0 |

H - Hare *(Lepus europaeus)*,*Final consensus sequence - HHCV.
